# Room‐Temperature Helix‐to‐Disc Conversion of Thia[6]helicene *S*,*S*‐Dioxides to Coronene via In Situ Phenoxide Anion Generation

**DOI:** 10.1002/chem.70843

**Published:** 2026-03-04

**Authors:** Kaito Seino, Takashi Murase

**Affiliations:** ^1^ Faculty of Science Yamagata University Yamagata Japan

**Keywords:** annulation, cycloaddition, helicene, pericyclic reaction, reaction mechanisms

## Abstract

Intramolecular annulation between the helical termini of helicenes provides a direct route to circulene‐type architectures. In thia[6]helicene, oxidation at the thiophene terminus to the *S*,*S*‐dioxide facilitates the thermal conversion to coronene, but established protocols typically require elevated temperatures. Herein, we demonstrate that forming a phenoxide anion at the benzene terminus of thia[6]helicene *S*,*S*‐dioxides enables this transformation at room temperature, affording coronene in near‐quantitative yield. Phenoxide arises either by deacetylation of the acetate precursor or by deprotonation of the corresponding phenol. Owing to the solubility contrast between the helical precursor and planar coronene, the product precipitates from solution, allowing the conversion to be monitored visually, and can be isolated by simple filtration. This helix‐to‐disc conversion begins with a stepwise intramolecular Diels–Alder reaction, and computational analysis elucidates the complete pathway to coronene. The present strategy delivers a practical room‐temperature route to planar aromatic frameworks via an anion‐triggered Diels–Alder reaction, without oxidative Scholl‐type conditions, and broadens opportunities for the late‐stage planarization of helicenes and related π‐systems.

## Graphical abstract

In situ phenoxide formation at the benzene terminus triggers room‐temperature conversion of thia[6]helicene *S*,*S*‐dioxides to coronene. The solubility contrast drives product precipitation, enabling isolation by simple filtration. This pericyclic annulation between the helical termini provides direct access to coronene‐type planar architectures.

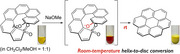

## 1 Introduction

The skeletal transformation of nonplanar π‐conjugated molecules into planar polycyclic aromatic frameworks represents a powerful synthetic strategy for reconfiguring molecular topology and accessing π‐extended architectures [1, 2, 3, 4, 5]. Within this paradigm, transformations into circulene‐type systems have proven particularly effective for constructing circumarenes formed by annulation around a central aromatic core [6, 7, 8, 9, 10]. Beyond structural reconfiguration, soluble precursors are often converted into less‐soluble products upon planarization, unless solubilizing substituents are incorporated into their final frameworks. Although this decrease in solubility may appear disadvantageous at first glance, it becomes a practical advantage when the conversion is clean and efficient. In such cases, precipitation enables the straightforward isolation and purification of otherwise intractable planar polycyclic aromatics.

**SCHEME 1:**
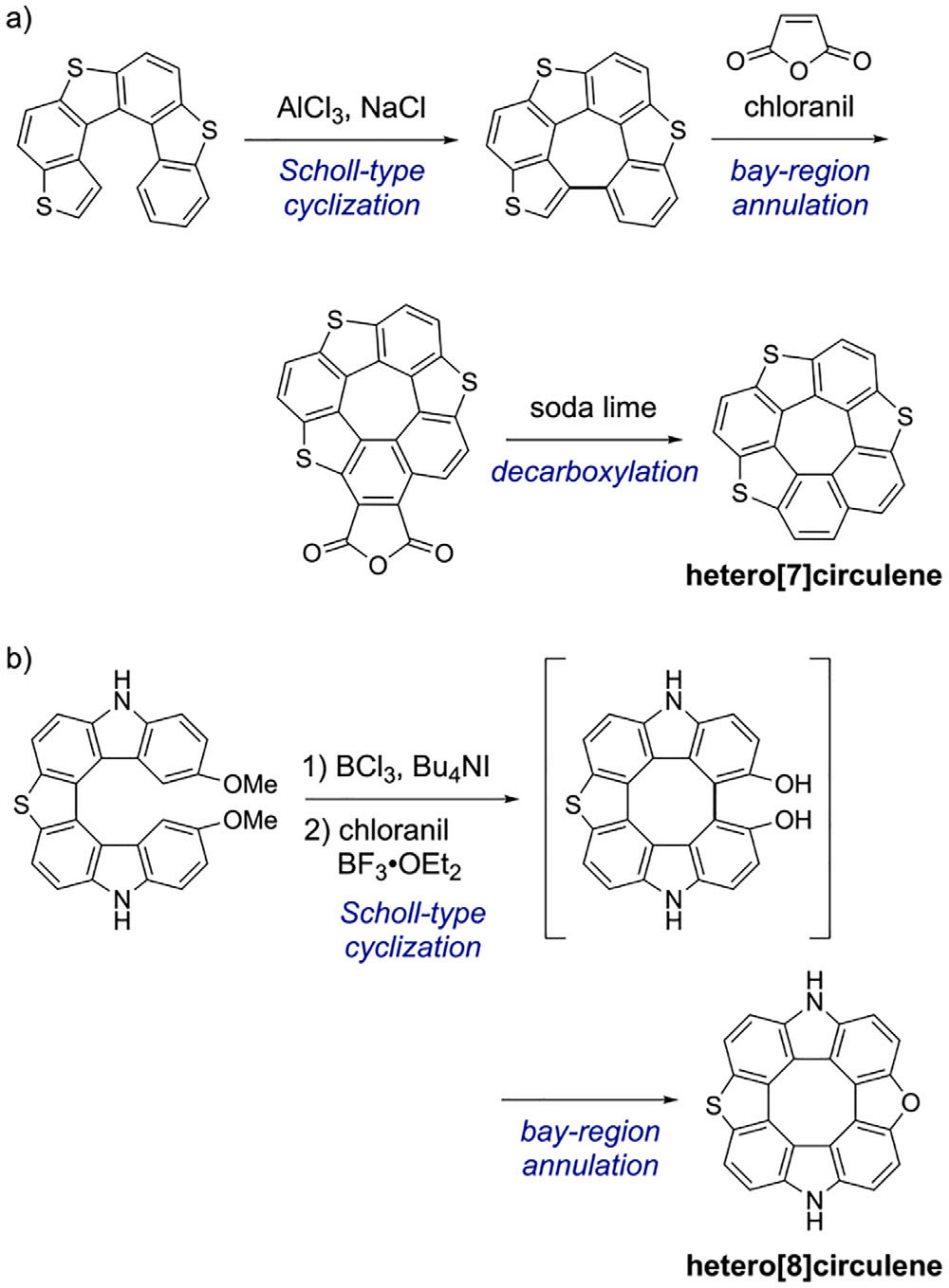
Previously developed strategies for connecting the helical termini of helicenes. Synthesis of a) hetero[7]circulene and b) hetero[8]circulene via intramolecular cyclization followed by bay‐region annulation.

The concept of connecting the helical termini of helicenes to effect planarization dates back to the 1970s, when Wynberg et al. explored the Scholl‐type reactions of sulfur‐containing helicenes (thiahelicenes) (Scheme 1a) [11, 12, 13]. Some thia[5] and thia[6]helicenes, with outward‐oriented sulfur atoms, underwent intramolecular ring closure, whereas the parent carbo[6]helicene did not. The resulting dehydrohelicenes featured newly formed bay regions that underwent Diels–Alder reactions with maleic anhydride, introducing two additional carbon atoms into the frameworks. Subsequent pyrolysis with soda lime induced decarboxylation of the Diels–Alder adducts, yielding heterocirculenes. This stepwise strategy—intramolecular Scholl‐type cyclization followed by bay‐region annulation—laid the foundation for planarizing helicenes and constructing π‐extended architectures.

More recently, alternative strategies have emerged that enable the direct construction of heterocirculenes from helicene precursors without isolating dehydrohelicene intermediates. For example, Pittelkow et al. reported a sequential annulation strategy in which 2,7‐dimethoxy‐substituted hetero[7]helicenes underwent oxidative cyclodehydrogenation under Scholl‐type conditions to afford fully planarized hetero[8]circulenes (Scheme 1b) [14]. This transformation begins with intramolecular C–C bond formation to close the inner ring, followed by cyclodehydration of the two terminal OH groups to furnish the outer furan ring. Building on this strategy, Ema et al. applied a similar approach to access saddle‐shaped hetero[8]circulenes, [15, 16] highlighting its applicability to a broader range of nonplanar heterocirculenes.

**SCHEME 2:**
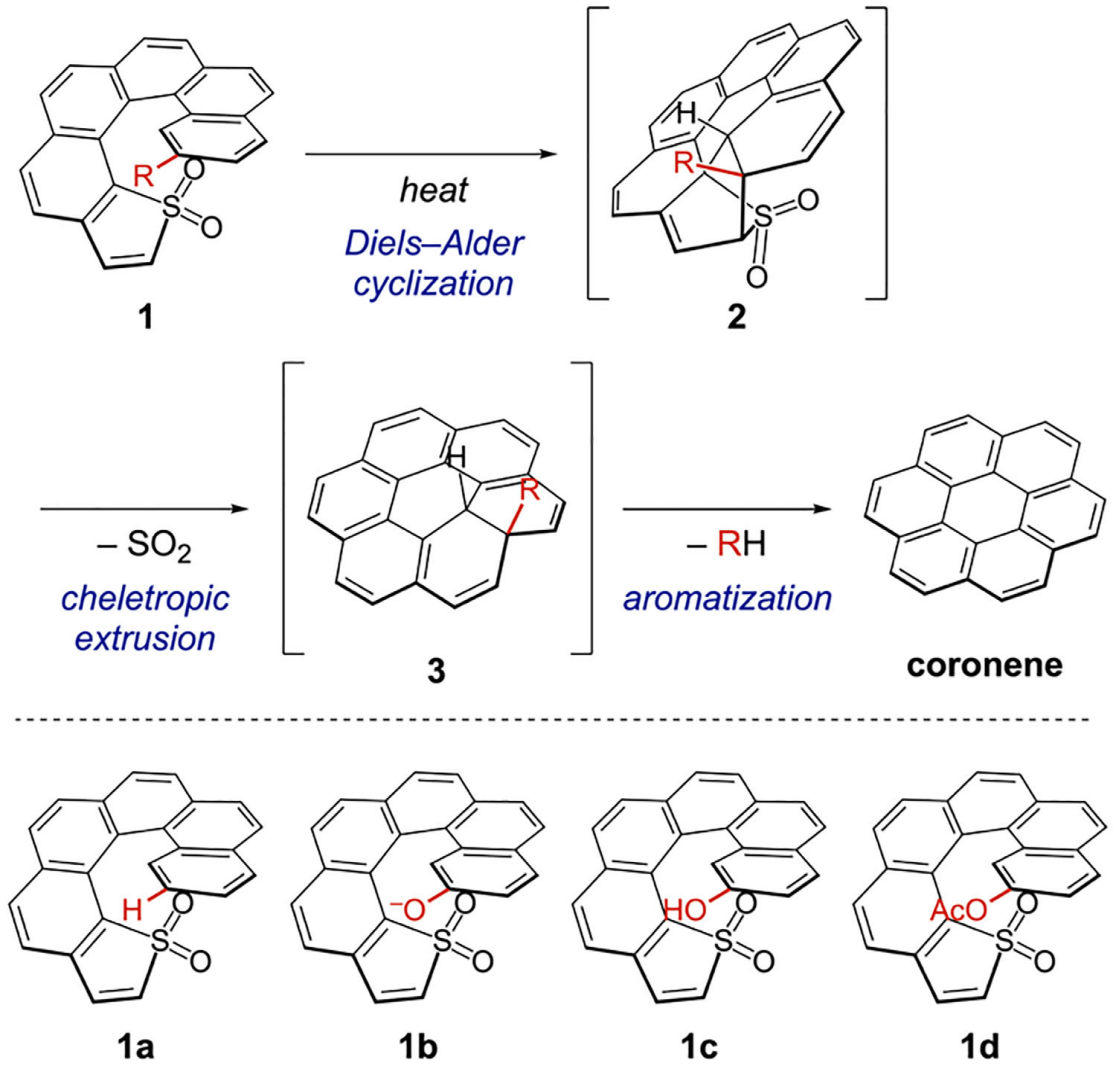
Pericyclic annulation at the helical termini of thia[6]helicene *S*,*S*‐dioxides **1** to give coronene. Helical structures are shown as the *M* enantiomer for clarity, but all samples were used as racemic mixtures.

In contrast to the widely employed Scholl‐type approach, we demonstrated that thia[6]helicene with an inward‐oriented sulfur atom at the terminal thiophene ring transforms into [6]circulene (i.e., coronene) under UV light irradiation [17]. This “helix‐to‐disc” conversion becomes thermally accessible upon *S*‐oxidation of the thiophene ring to the corresponding *S*,*S*‐dioxide **1a** (Scheme 2; R = H). The skeletal transformation of **1a** starts with intramolecular Diels–Alder cyclization (**1a→2a**), followed by cheletropic extrusion of sulfur dioxide (**2a→3a**) and aromatization of the 9,10‐dihydronaphthalene moiety (**3a→coronene**). Our pericyclic strategy links the helical termini to form coronene‐type fused systems, bypassing bay‐region annulation and providing a direct pathway to circulene‐type planarization. Note that the transformation is driven by external stimuli and requires no additional oxidants, underscoring its operational simplicity.

However, the transformation of **1a** still requires relatively harsh conditions, typically heating to 160°C for efficient coronene formation. At lower temperatures, such as 100°C, the reaction becomes sluggish and leads to the deposition of uncharacterized insoluble material, which diminishes efficiency. Seeking a route that would permit lower temperatures, we examined substituent effects and reaction conditions. Herein, we demonstrate that forming aryloxide (ArO^−^) **1b**, with O^−^ directly bound at the benzene terminus, enables clean and near‐quantitative helix‐to‐disc conversion to coronene at room temperature (Scheme 2). Notably, **1b** undergoes smooth conversion, whereas the corresponding conjugate acid (ArOH) **1c** remains unreactive at room temperature. Density functional theory (DFT) calculations provide a mechanistic rationale for this aryloxide‐enabled conversion to coronene, consistent with the observed ambient reactivity.

## 2 Results and Discussion

### 2.1 Synthesis of Functionalized Thia[6]helicene *S*,*S*‐Dioxides

In our previous reports on the synthesis of **1a**, the helical framework was assembled starting from the terminal benzene ring, with the thiophene ring at the opposite terminus being introduced at the final stage [17, 18]. Although this approach was effective for preparing the parent framework, it was unsuitable for the present study, which required functionalization at the terminal benzene ring. In particular, when the optimal substituent has not been predetermined at the outset, this synthetic order offers limited versatility in the late‐stage introduction of functional groups.

**SCHEME 3:**
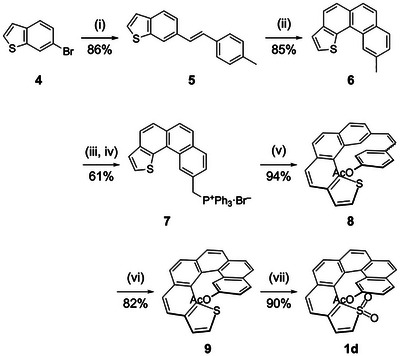
Synthesis of **1d**. Conditions: (i) 4‐methylstylene, NaOAc, Herrmann's catalyst, *N*,*N*‐dimethylacetamide, 130°C, 48 h; (ii) UV light, I_2_, propylene oxide, toluene, rt, 1.5 h; (iii) NBS, AIBN, CCl_4_, reflux, 16 h; (iv) PPh_3_, toluene, reflux, 5 h; (v) 4‐acetoxybenzaldehyde, NaH, THF, 0°C, 16 h; (vi) UV light, I_2_, propylene oxide, cyclohexane/toluene, rt, 20 min; (vii) *m*‐CPBA, CH_2_Cl_2_, rt, 6 h. rt: room temperature.

To address this limitation, we redesigned the synthetic route to enable late‐stage functionalization, as shown in Scheme 3. A Mizoroki–Heck reaction between commercially available 6‐bromobenzothiophene (**4**) and 4‐methylstyrene afforded compound **5** in 86% yield. Subsequent oxidative photocyclization of **5** afforded thia[4]helicene **6** in 85% yield. Benzylic bromination of **6** with *N*‐bromosuccinimide (NBS), followed by treatment with PPh_3_, provided phosphonium salt **7** (61%, two steps). This salt served as a versatile substrate in the subsequent Wittig reaction for preparing functionalized thia[6]helicenes. In this study, the Wittig reaction of **7** with 4‐acetoxybenzaldehyde furnished stilbene **8** (94%), bearing an acetyl‐protected phenol functionality amenable to later deprotection. Subsequent oxidative photocyclization of **8** extended the helical framework to give thia[6]helicene **9** in 82% yield. Finally, the terminal thiophene moiety was oxidized with *m*‐chloroperbenzoic acid (*m*‐CPBA), affording acetoxy‐functionalized thia[6]helicene *S*,*S*‐dioxide **1d** in 90% yield.

### 2.2 Conversion to Coronene via In Situ Phenoxide Anion Generation

**SCHEME 4:**
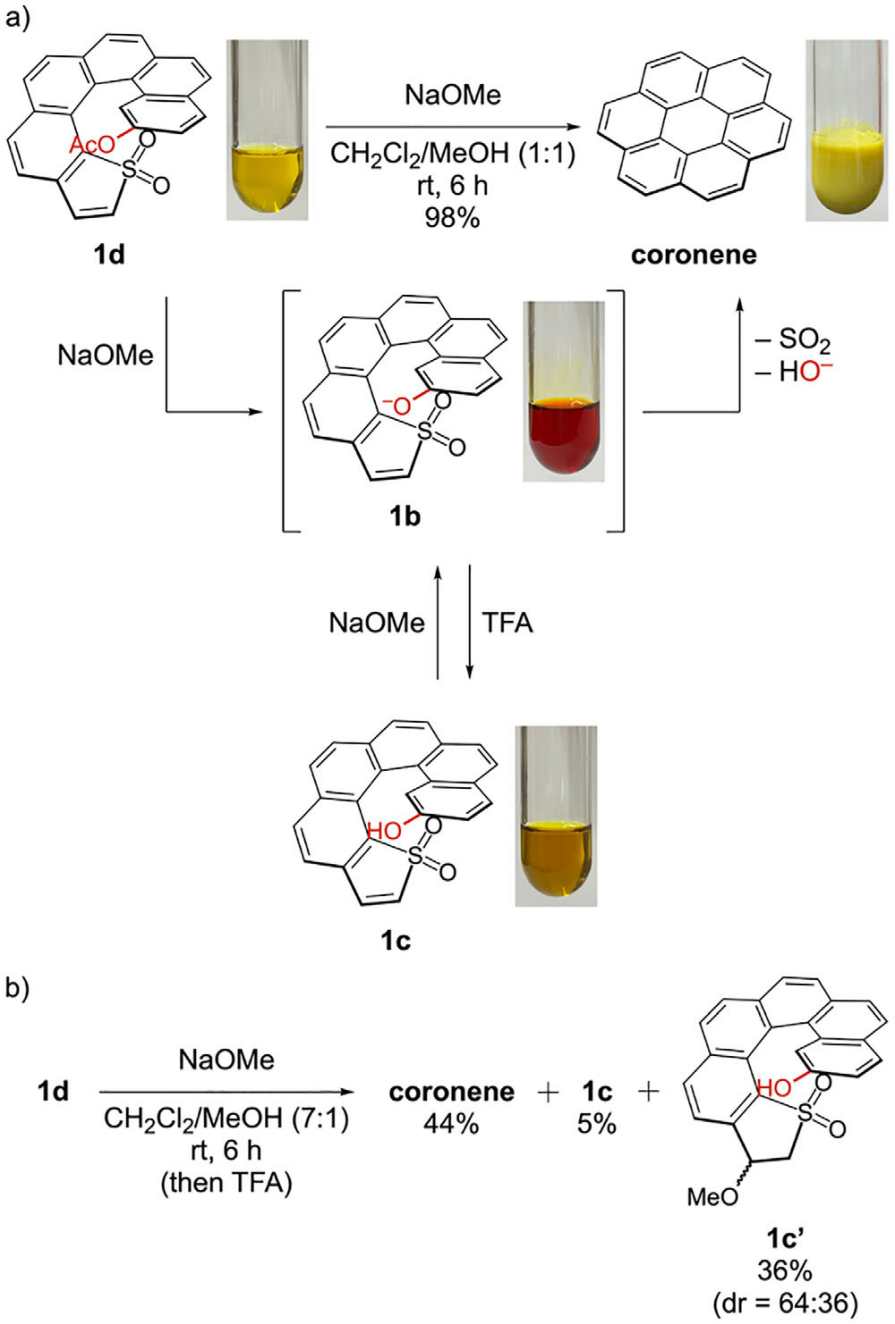
a) Photographs illustrating the color and phase changes during the helix‐to‐disc conversion of **1d** in CH_2_Cl_2_/MeOH (1:1) at room temperature. The lower panel shows TFA quenching of the NaOMe‐treated solution, affording **1c**. b) Treatment of **1d** with NaOMe in CH_2_Cl_2_/MeOH (7:1) at room temperature, which suppresses coronene formation and gives the deacetylated oxa‐Michael adduct **1c’**, as a mixture of diastereomers (dr = 64:36). rt: room temperature; dr: diastereomeric ratio.

When a CH_2_Cl_2_/MeOH (1:1) solution of **1d** (49 mM) was treated with NaOMe (3.0 equiv.) at room temperature for deacetylation, the yellow solution immediately turned reddish orange (Scheme 4a). Upon continued stirring at room temperature, the solution gradually decolorized to pale yellow, accompanied by the precipitation of a yellow solid. After 6 h, the precipitate was collected by filtration to afford spectroscopically pure coronene in 64% yield (Figure S1). The supernatant contained dissolved coronene, but **1d** was not detected by ^1^H NMR. Subsequent concentration and trituration with MeOH gave additional coronene (34%), bringing the total isolated yield to 98%.

To capture the deacetylated product **1c** before conversion to coronene, we immediately quenched the reaction with trifluoroacetic acid (TFA) after 3 min of NaOMe treatment, keeping other conditions unchanged (Scheme 4a). The reddish‐orange solution turned deep yellow, but no precipitate formed at this stage. After solvent removal, the addition of MeOH induced precipitation, and the mixture was filtered. NMR analysis of the precipitate and the filtrate confirmed complete deacetylation of **1d** to give **1c**, with a trace amount of coronene (Figure S2). The filtrate was then worked up and purified by column chromatography on silica gel to give an additional **1c**‐rich fraction. Overall, **1c** and coronene were obtained in 78% and 3% yields, respectively. Isolated **1c** shows poor solubility in chlorinated solvents (CH_2_Cl_2_ and CHCl_3_) and aromatic solvents (toluene and mesitylene) but readily dissolves in polar aprotic solvents (acetone and dimethyl sulfoxide (DMSO)).

Because **1c** was stable at room temperature and only converted to coronene upon heating in DMSO (160°C, 1.5 h, 54%), we reasoned that the phenolic OH alone did not provide sufficient electron donation to trigger the transformation at room temperature. Accordingly, we hypothesized that deprotonation to aryloxide **1b** would enhance electron donation at the dienophilic benzene ring and promote the transformation. Consistent with this hypothesis, treatment of **1c** with NaOMe turned the yellow solution reddish orange again. The reaction then smoothly proceeded to afford coronene, confirming that **1b** is the reactive species responsible for the room‐temperature helix‐to‐disc conversion.

Moreover, the solvent composition proved crucial for directing the reaction pathway. The transformation of **1d** proceeded cleanly in CH_2_Cl_2_/MeOH (1:1), but this solvent mixture serves not only to facilitate the precipitation of coronene but also to prevent the competing oxa‐Michael addition [19, 20, 21] of MeO^−^ to the electron‐deficient thiophene *S*,*S*‐dioxide unit [22, 23]. A higher MeOH content is expected to attenuate the effective nucleophilicity of MeO^−^ through protic solvation and hydrogen bonding, thereby disfavoring this oxa‐Michael pathway. At the same time, MeOH‐rich conditions promote deacetylation by facilitating the methanolysis of acetate **1d**. When the reaction was carried out in CH_2_Cl_2_/MeOH (7:1), coronene formation was suppressed (44%), and the deacetylated oxa‐Michael adduct **1c’** was obtained in 36% yield, along with **1c** in 5% yield (Scheme 4b). This methoxide addition at the β‐position of the thiophene *S*,*S*‐dioxide unit disrupts the conjugated diene motif required for the intramolecular Diels–Alder step, thereby suppressing the helix‐to‐disc conversion under CH_2_Cl_2_‐rich conditions.

### 2.3 DFT‐Supported Mechanism of Coronene Formation

Historically, intramolecular Diels–Alder reactions of helicenes and subsequent spontaneous transformations have not yielded planar frameworks as final products [24, 25, 26, 27, 28, 29, 30, 31]. Within [7]helicene frameworks, the terminal benzene ring couples with the second distal benzene ring, leaving the opposite terminal ring outside the fused core. Efficient conversion has required strong activation, such as the generation of a benzyne dienophile at the terminal ring, [25] Lewis‐acid mediation, [26, 27] high‐temperature annealing on Ag surfaces, [28] or photochemical excitation [29, 30, 31]. Against this background, it is remarkable that **1b** can undergo a helix‐to‐disc conversion to coronene at room temperature, necessitating further investigation.

To address this, we conducted DFT calculations; specifically, geometry optimization and frequency calculations were performed at the M06‐2X/6‐31+G(d,p) level with the conductor‐like polarizable continuum model (CPCM) (for MeOH). Single‐point energies were refined at the M06‐2X/6‐311+G(2d,p) level with the solvation model based on density (SMD) (for MeOH). The free energies reported at 298 K combine the SMD/6‐311+G(2d,p) electronic energies with the CPCM/6‐31+G(d,p) thermal corrections (for details, see the Supporting Information).

**FIGURE 1:**
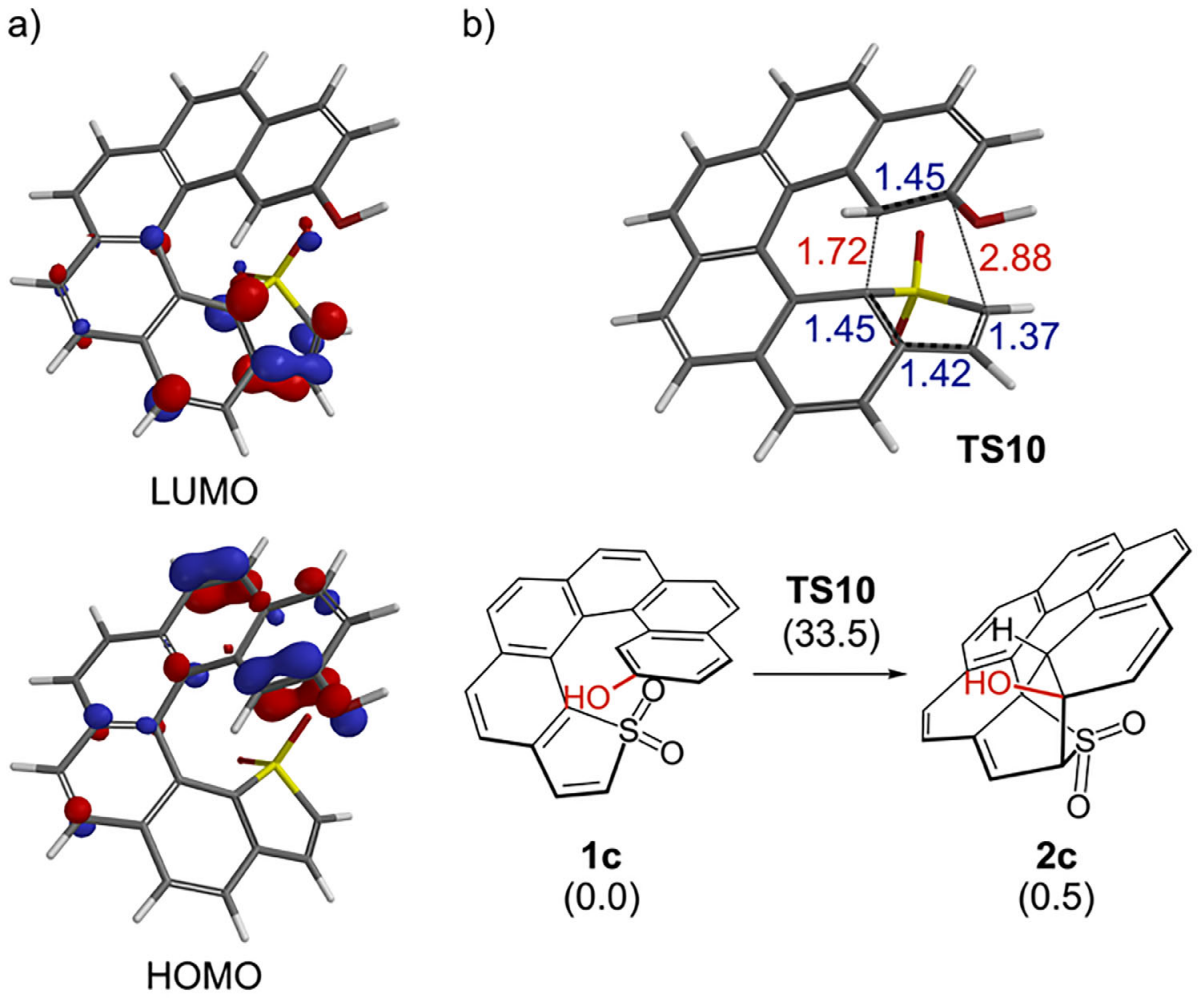
DFT analysis for the intramolecular Diels–Alder reaction of **1c** to **2c**. a) HOMO and LUMO isosurfaces of **1c** at an isovalue of 0.07. b) Concerted asynchronous transition state **TS10** with forming C─C bonds of 1.72 Å on the inner helix and 2.88 Å on the outer helix. The calculated energies (kcal mol^−1^) relative to **1c** are shown in parentheses. Energies were computed at the SMD(MeOH)‐M06‐2X/6‐311+G(2d,p) level on CPCM(MeOH)‐M06‐2X/6‐31+G(d,p) geometries at 298 K.

As a preliminary validation, we first examined the intramolecular Diels–Alder reaction of **1c** at 298 K (Figure 1). The calculations indicate a concerted asynchronous pathway with a single transition state (**TS10**) located at an activation free energy of 33.5 kcal mol^−1^ (34.6 kcal mol^−1^ in DMSO at 433 K reflects the experimental conditions), consistent with the lack of reactivity of **1c** at room temperature (Figure 1b). The forming C─C bond on the inner helix (1.72 Å) is more advanced than that on the outer helix (2.88 Å). The computed reaction free energy for **1c→2c** is 0.5 kcal mol^−1^, indicating an essentially thermoneutral process. Frontier orbital analysis further supports an inverse electron‐demand character in which the highest occupied molecular orbital (HOMO) is localized on the electron‐rich terminal phenol ring (dienophile), whereas the lowest unoccupied molecular orbital (LUMO) is located on the electron‐deficient thiophene *S*,*S*‐dioxide ring (diene) (Figure 1a). Such intramolecular inverse electron‐demand Diels–Alder reactions involving thiophene *S*,*S*‐dioxides have recently attracted considerable attention as powerful tools for the rapid construction of complex molecular architectures [32, 33, 34, 35, 36]. Anticipating that deprotonation would raise the dienophilic HOMO and lower the activation free energy, we next analyzed the anionic species **1b**. However, no concerted pathway was identified for **1b**, suggesting that the reaction proceeded in a stepwise fashion.

**FIGURE 2:**
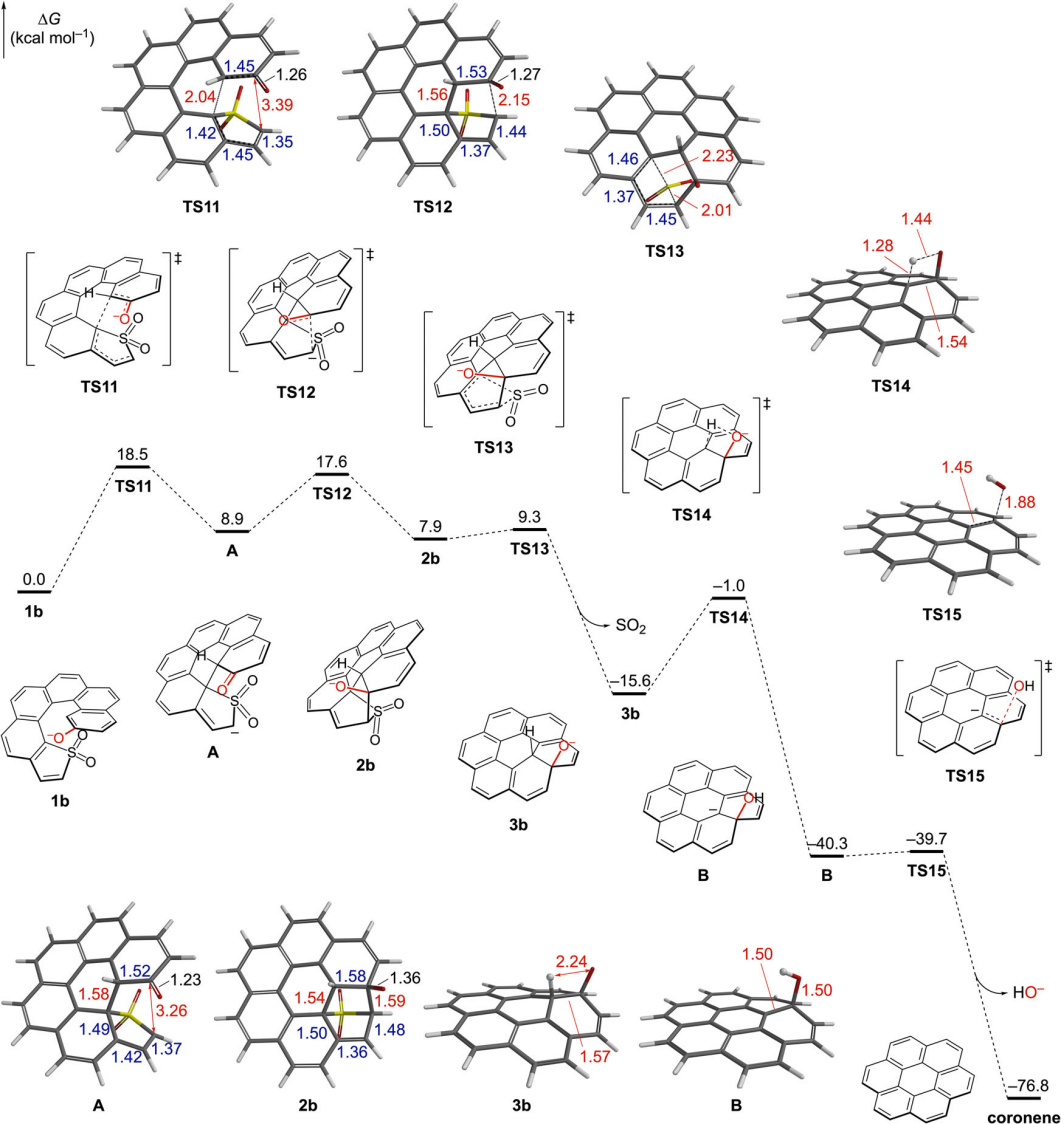
Calculated relative free‐energy profile for the transformation of **1b** into coronene. Relative Gibbs free energies are shown in kcal mol^−1^. Geometric structures of key transition states (**TS11**–**TS15**) and intermediates (**A, 2b**, **3b**, **B**) are shown with selected interatomic distances (Å). Energies were computed at the SMD(MeOH)‐M06‐2X/6‐311+G(2d,p) level on CPCM(MeOH)‐M06‐2X/6‐31+G(d,p) geometries at 298 K.

A stepwise pathway involving an initial Michael‐type addition followed by an intramolecular aldol‐type addition is characteristic of Diels–Alder reactions between highly polarized diene–dienophile pairs [37, 38, 39, 40, 41, 42, 43]. In line with this mechanistic paradigm, the reaction of **1b** was found to proceed in a stepwise manner (Figure 2). The first transition state (**TS11**) corresponds to C–C bond formation within the inner helix, leading to intermediate **A**. This intermediate displays a newly formed C–C bond of 1.58 Å, while the remaining reactive carbons are separated by 3.26 Å, reflecting a partially completed cycloaddition. Intermediate **A** subsequently undergoes the second C–C bond formation via **TS12** to furnish the Diels–Alder product **2b**. The overall barrier for this Diels–Alder process is 18.5 kcal mol^−1^, which is reduced by 15.0 kcal mol^−1^ relative to the neutral species **1c**, sufficient to render the reaction feasible at room temperature.

Although the Diels–Alder reaction of **1b** to **2b** is endergonic and thermodynamically uphill by 7.9 kcal mol^−1^, the subsequent cheletropic extrusion of SO_2_ (**2b→3b**) provides a strong thermodynamic driving force [32, 33, 34, 35, 36]. This step is exergonic (−23.5 kcal mol^−1^) and proceeds with an almost negligible barrier (1.4 kcal mol^−1^) via **TS13**, affording the partially saturated coronene oxide anion **3b**. The substantial decrease in free energy associated with SO_2_ release compensates for the unfavorable thermodynamics of the preceding Diels–Alder step, thereby driving the overall transformation toward the products.

Assuming an anionic pathway up to the elimination step that furnishes coronene, the late‐stage sequence begins with intramolecular proton abstraction; the bridgehead C─O^−^ unit of **3b** deprotonates the adjacent bridgehead C─H. Computations show a modest barrier of 14.6 kcal mol^−1^ for this proton transfer via **TS14**, forming carbanion **B**, which is stabilized by extensive conjugation. As illustrated in Figure 2, the optimized geometries show progressive planarization around the reacting bridgehead, from puckered **3b** through **TS14** to the nearly coplanar **B**. This conjugate base lies 24.7 kcal mol^−1^ below **3b** in free energy, and this stabilization promotes E1cB‐type elimination. The subsequent elimination step proceeds via **TS15**, and the loss of HO^−^ furnishes coronene. This elimination is strongly exergonic (−36.5 kcal mol^−1^) and effectively barrierless, with the recovery of aromatic stabilization providing an additional driving force for the overall transformation.

## 3 Conclusion

We demonstrated that thia[6]helicene *S*,*S*‐dioxides underwent helix‐to‐disc conversion to coronene at room temperature, owing to the in situ generation of aryloxide **1b**, which arises either by direct deprotonation of **1c** or by deacetylation of **1d**. Operationally, NaOMe in CH_2_Cl_2_/MeOH (1:1) gave a clean conversion, and the product precipitated, simplifying isolation. DFT calculations accounted for these observations. For **1c**, a concerted intramolecular Diels–Alder pathway had a high barrier at 298 K, consistent with its lack of reactivity. For **1b**, the Diels–Alder reaction proceeded stepwise and culminated in coronene formation. The helicene skeleton became progressively more planar along the pathway, consistent with the recovery of aromatic stabilization as an additional driving force. The present strategy, which exploits the intrinsic intramolecular Diels–Alder reactivity of helicenes, offers a simple route for constructing planar π‐extended frameworks at room temperature and broadens opportunities for the late‐stage planarization of helicenes and related π‐systems.

## 4 Experimental Section

### 4.1 Transformation of **1d** into Coronene at Room Temperature

Thia[6]helicene *S*,*S*‐dioxide **1d** (33.3 mg, 0.0785 mmol) was dissolved in CH_2_Cl_2_/MeOH (1.6 mL, 1:1 v/v). Then, a solution of NaOMe in MeOH (1.0 M, 0.235 mL, 0.235 mmol) was added dropwise, and the resulting solution (overall CH_2_Cl_2_/MeOH ≈ 1:1.3, v/v) was stirred at room temperature for 6 h. After thin‐layer chromatography confirmed completion of the reaction with a single new spot corresponding to coronene, the resulting precipitate was collected by filtration and washed with MeOH to give coronene (15.1 mg, 0.0503 mmol, 64%). The filtrate was concentrated by rotary evaporation, and MeOH (2 mL) was added. The suspension was heated at 45°C, and the resulting precipitate was collected by filtration and washed with cold MeOH to give coronene (5.1 mg, 0.0170 mmol, 22%). The mother liquor was again concentrated by rotary evaporation, MeOH (0.5 mL) was added, and the suspension was reheated at 45°C. The resulting precipitate was collected by filtration and washed with cold MeOH to give coronene (2.8 mg, 9.3×10^−3^ mmol, 12%). Thus, the total yield of coronene was 98% (23.0 mg, 0.0766 mmol). All isolated samples of coronene were confirmed to be analytically pure by ^1^H NMR spectroscopy in CDCl_3_, showing a single resonance at *δ* = 8.94 ppm (Figure S1).

### 4.2 Computational Methods

All DFT calculations were carried out with Spartan’24 (Wavefunction, Inc.) using the M06‐2X functional. Geometry optimizations and harmonic frequency calculations were performed at the M06‐2X/6‐31+G(d,p) level with the CPCM solvation model (MeOH), and single‐point electronic energies were refined at the M06‐2X/6‐311+G(2d,p) level with the SMD solvation model (MeOH) on the CPCM‐optimized geometries. All stationary points were characterized by frequency analyses (minima: no imaginary frequencies; transition states: one imaginary frequency). Gibbs free energies were obtained by combining single‐point electronic energies with thermal corrections from the frequency calculations.

Low‐frequency vibrational contributions were treated using a quasi‐harmonic approach to avoid the overestimation of entropic terms. A standard state correction from 1 atm to 1 M was applied using the conventional concentration correction (+1.89 kcal mol^−1^ at 298.15 K). Explicit metal counterions (e.g., Na^+^) were not included to avoid model‐dependent contact or solvent‐separated ion pairs. Anionic species were treated as free anions within the continuum solvent model. Hydroxide was modeled using an effective free‐energy approach based on an HO^−^···MeOH cluster (see the Supporting Information for details). The experimental CH_2_Cl_2_/MeOH (1:1) medium was approximated as MeOH because the key elementary steps involve anionic species and specific hydrogen‐bonding/proton‐transfer stabilization dominated by the protic component, and the continuum treatment of mixed solvents is not always reliable.

## Conflicts of Interest

The authors declare no conflicts of interest.

## Supporting information



The authors have cited additional references within the Supporting Information [S1–S3].

## Data Availability

The data that supports the findings of this study are available in the supplementary material of this article

## References

[1] A. Narita , X.‐Y. Wang , X. Feng , and K. Müllen , “New Advances in Nanographene Chemistry,” Chemical Society Reviews 44 (2015): 6616–6643, 10.1039/C5CS00183H. 26186682

[2] Y. Segawa , H. Ito , and K. Itami , “Structurally Uniform and Atomically Precise Carbon Nanostructures,” Nature Reviews Materials 1 (2016): 15002, 10.1038/natrevmats.2015.2.

[3] M. Grzybowski , B. Sadowski , H. Butenschön , and D. T. Gryko , “Synthetic Applications of Oxidative Aromatic Coupling—From Biphenols to Nanographenes,” Angewandte Chemie International Edition 59 (2020): 2998–3027, 10.1002/anie.201904934. 31342599 PMC7027897

[4] A. Borissov , Y. K. Maurya , L. Moshniaha , W.‐S. Wong , M. Żyła‐Karwowska , and M. Stępień , “Recent Advances in Heterocyclic Nanographenes and Other Polycyclic Heteroaromatic Compounds,” Chemical Reviews 122 (2022): 565–788, 10.1021/acs.chemrev.1c00449. 34850633 PMC8759089

[5] Y. Zhang , S. H. Pun , and Q. Miao , “The Scholl Reaction as a Powerful Tool for Synthesis of Curved Polycyclic Aromatics,” Chemical Reviews 122 (2022): 14554–14593, 10.1021/acs.chemrev.2c00186. 35960873

[6] R. D. Broene and F. Diederich , “The Synthesis of Circumanthracene,” Tetrahedron Letters 32 (1991): 5227–5230, 10.1016/S0040-4039(00)92350-5.

[7] M. R. Ajayakumar , Y. Fu , J. Ma , et al., “Toward Full Zigzag‐Edged Nanographenes: *peri*‐Tetracene and Its Corresponding Circumanthracene,” Journal of the American Chemical Society 140 (2018): 6240–6244, 10.1021/jacs.8b03711. 29738244

[8] Q. Jiang , H. Wei , X. Hou , and C. Chi , “Circumpentacene with Open‐Shell Singlet Diradical Character,” Angewandte Chemie International Edition 62 (2023): e202306938, 10.1002/anie.202306938. 37338045

[9] J. Hu , Q. Xiang , X. Tian , et al., “S‐Shaped Helical Singlet Diradicaloid and Its Transformation to Circumchrysene via a Two‐Stage Cyclization,” Journal of the American Chemical Society 146 (2024): 10321–10330, 10.1021/jacs.3c11585. 38567901

[10] Q. Jiang , J. He , F. Gu , et al., “Synthesis and Characterization of Circumtetracene: Unraveling Structure‐Property Relationships of Circumacenes,” Angewandte Chemie International Edition 64 (2025): e202506248, 10.1002/anie.202506248. 40194951

[11] M. B. Groen , H. Schadenberg , and H. Wynberg , “Synthesis and Resolution of Some Heterohelicenes,” Journal of Organic Chemistry 36 (1971): 2797–2809, 10.1021/jo00818a016.

[12] J. H. Dopper and H. Wynberg , “Synthesis and Properties of Some Heterocirculenes,” Journal of Organic Chemistry 40 (1975): 1957–1966, 10.1021/jo00901a019.

[13] J. H. Dopper , D. Oudman , and H. Wynberg , “Dehydrogenation of Heterohelicenes by a Scholl Type Reaction. Dehydrohelicenes,” Journal of Organic Chemistry 40 (1975): 3398–3401, 10.1021/jo00911a020.

[14] B. Lousen , S. K. Pedersen , P. Bols , et al., “Compressing a Non‐Planar Aromatic Heterocyclic [7]Helicene to a Planar Hetero[8]Circulene,” Chemistry—A European Journal 26 (2020): 4935–4940, 10.1002/chem.201905339. 32052498

[15] C. Maeda , S. Nomoto , K. Akiyama , T. Tanaka , and T. Ema , “Facile Synthesis of Azahelicenes and Diaza[8]circulenes Through the Intramolecular Scholl Reaction,” Chemistry—A European Journal 27 (2021): 15699–15705, 10.1002/chem.202102269. 34449114

[16] C. Maeda , K. Akiyama , and T. Ema , “Synthesis and Photophysical Properties of Dihetero[8]circulenes,” Organic Letters 25 (2023): 3932–3935, 10.1021/acs.orglett.3c01304. 37222493

[17] K. Seino , T. Okano , K. Oya , H. Katagiri , and T. Murase , “Helix‐to‐Disc Conversion of Thia[6]helicenes into Coronenes Facilitated by Sulfur Oxidation and Fluorination,” Chemistry—A European Journal 30 (2024): e202402445, 10.1002/chem.202402445. 39051923

[18] A. Nakao , H. Katagiri , and T. Murase , “Transformation of Silyl‐Protected Tetrafluorinated Thia[6]helicene S‐Oxide into a Difluorinated Coronene via Induced Desilylation,” Chemistry—A European Journal 31 (2025): e02242, 10.1002/chem.202502242. 40855772 PMC12520054

[19] C. F. Nising and S. Bräse , “The Oxa‐Michael Reaction: From Recent Developments to Applications in Natural Product Synthesis,” Chemical Society Reviews 37 (2008): 1218–1228, 10.1039/b718357g. 18497934

[20] C. F. Nising and S. Bräse , “Recent Developments in the Field of Oxa‐Michael Reactions,” Chemical Society Reviews 41 (2012): 988–999, 10.1039/C1CS15167C. 21796323

[21] Y. Wang and D.‐M. Du , “Recent Advances in Organocatalytic Asymmetric Oxa‐Michael Addition Triggered Cascade Reactions,” Organic Chemistry Frontiers 7 (2020): 3266–3283, 10.1039/D0QO00631A.

[22] H. Chen , Z,Yang, C. Ding , et al., “Discovery of Potent Anticancer Agent HJC0416, an Orally Bioavailable Small Molecule Inhibitor of Signal Transducer and Activator of Transcription 3 (STAT3),” European Journal of Medicinal Chemistry 82 (2014): 195–203, 10.1016/j.ejmech.2014.05.049. 24904966 PMC4096847

[23] P. Oleksak , D. Rysanek , M. Vancurova , et al., “Discovery of a 6‐Aminobenzo[*b*]thiophene 1,1‐Dioxide Derivative (K2071) with a Signal Transducer and Activator of Transcription 3 Inhibitory, Antimitotic, and Senotherapeutic Activities,” ACS Pharmacology & Translational Science 7 (2024): 2755–2783, 10.1021/acsptsci.4c00190. 39296273 PMC11406704

[24] R. H. Martin , J. Jespers , and N. Defay , “Helicenes: Thermally Induced Intramolecular 4 + 2 Cycloadditions Involving the [6]Helicene Skeleton,” Helvetica Chimica Acta 58 (1975): 776–779, 10.1002/hlca.19750580314.

[25] D. Z. Wang , T. J. Katz , J. Golen , and A. L. Rheingold , “Diels−Alder Additions of Benzynes Within Helicene Skeletons,” Journal of Organic Chemistry 69 (2004): 7769–7771, 10.1021/jo048707h. 15498014

[26] M. J. Fuchter , M. Weimar , X. Yang , D. K. Judge , and A. J. P. White , “An Unusual Oxidative Rearrangement of [7]‐Helicene,” Tetrahedron Letters 53 (2012): 1108–1111, 10.1016/j.tetlet.2011.12.082.

[27] R. J. F. Berger , M. J. Fuchter , I. Krossing , H. S. Rzepa , J. Schaefer , and H. Scherer , “Gold(I) Mediated Rearrangement of [7]‐Helicene to Give a Benzo[*cd*]pyrenium Cation Embedded in a Chiral Framework,” Chemical Communications 50 (2014): 5251–5253, 10.1039/C3CC46986G. 24266024

[28] O. Stetsovych , M. Švec , J. Vacek , et al., “From Helical to Planar Chirality by on‐Surface Chemistry,” Nature Chemistry 9 (2017): 213–218, 10.1038/nchem.2662. 28221353

[29] T. Murase , C. Matsuda , K. Adachi , T. Sawada , and M. Fujita , “Triple Photochemical Domino Reaction of a Tetrafluorostilbene Terminating in Double Fluorine Atom Transfer,” Communications Chemistry 1 (2018): 97, 10.1038/s42004-018-0099-7.

[30] C. Matsuda , Y. Suzuki , H. Katagiri , and T. Murase , “Synthesis of Terminally Fluorinated [7]Helicenes and Their Application to Photochemical Domino Reactions,” Chemistry—An Asian Journal 16 (2021): 538–547, 10.1002/asia.202001295. 33471402

[31] C. Matsuda , R. Igarashi , H. Katagiri , and T. Murase , “Skeletal Transformation Triggered by C−F Bond Activation after Photochemical Rearrangement of Fluorinated [7]Helicenes,” Chemistry—A European Journal 28 (2022): e202200132, 10.1002/chem.202200132. 35174555

[32] S. Suzuki , T. Asako , K. Itami , and J. Yamaguchi , “Modular Synthesis of Heptaarylindole,” Organic & Biomolecular Chemistry 16 (2018): 3771–3776, 10.1039/C8OB00993G. 29736544

[33] A. Kabuki and J. Yamaguchi , “Formal Syntheses of Dictyodendrins B, C, and E by a Multi‐Substituted Indole Synthesis,” Synthesis 54 (2022): 4963–4970, 10.1055/a-1786-9881.

[34] K. H. K. Park , N. Frank , F. Duarte , and E. A. Anderson , “Collective Synthesis of Illudalane Sesquiterpenes via Cascade Inverse Electron Demand (4 + 2) Cycloadditions of Thiophene *S*,*S*‐Dioxides,” Journal of the American Chemical Society 144 (2022): 10017–10024, 10.1021/jacs.2c03304. 35609003 PMC9185749

[35] Z.‐S. Wang , S. H. Bennett , B. Kicin , et al., “De Novo Synthesis of Dihydrobenzofurans and Indolines and Its Application to a Modular, Asymmetric Synthesis of Beraprost,” Journal of the American Chemical Society 145 (2023): 14124–14132, 10.1021/jacs.3c04582. 37326516 PMC10311537

[36] S.‐X. Feng , Q.‐T. Lu , Y. Lu , et al., “Iron‐Catalyzed Asymmetric [4+2]/Cheletropic Retro‐[4+1] Cycloadditions of Thiophene *S*,*S*‐Dioxides With 3‐Substituted Indoles,” Angewandte Chemie International Edition 64 (2025): e202512345, 10.1002/anie.202512345. 40891673

[37] E. Goldstein , B. Beno , and K. N. Houk , “Density Functional Theory Prediction of the Relative Energies and Isotope Effects for the Concerted and Stepwise Mechanisms of the Diels−Alder Reaction of Butadiene and Ethylene,” Journal of the American Chemical Society 118 (1996): 6036–6043, 10.1021/ja9601494.

[38] C. R. W. Guimarães , M. Udier‐Blagović , and W. L. Jorgensen , “Macrophomate Synthase: QM/MM Simulations Address the Diels−Alder versus Michael−Aldol Reaction Mechanism,” Journal of the American Chemical Society 127 (2005): 3577–3588, 10.1021/ja043905b. 15755179

[39] L. R. Domingo and J. A. Sáez , “Understanding the Mechanism of Polar Diels–Alder Reactions,” Organic & Biomolecular Chemistry 7 (2009): 3576–3583, 10.1039/b909611f. 19675915

[40] H. V. Pham , D. B. C. Martin , C. D. Vanderwal , and K. N. Houk , “The Intramolecular Diels–Alder Reaction of Tryptamine‐Derived Zincke Aldehydes Is a Stepwise Process,” Chemical Science 3 (2012): 1650–1655, 10.1039/c2sc01072k. PMC3354770 22611483

[41] M. Linder and T. Brinck , “Stepwise Diels–Alder: More than Just an Oddity? A Computational Mechanistic Study,” Journal of Organic Chemistry 77 (2012): 6563–6573, 10.1021/jo301176t. 22780581

[42] E. M. Stocking and R. M. Williams , “Chemistry and Biology of Biosynthetic Diels–Alder Reactions,” Angewandte Chemie International Edition 42 (2003): 3078–3115, 10.1002/anie.200200534. 12866094

[43] B. R. Lichman , S. E. O'Connor , and H. Kries , “Biocatalytic Strategies towards [4+2] Cycloadditions,” Chemistry—A European Journal 25 (2019): 6864–6877, 10.1002/chem.201805412. 30664302

