# Supplementary material for: Room‐Temperature Helix‐to‐Disc Conversion of Thia[6]helicene S,S‐Dioxides to Coronene via In Situ Phenoxide Anion Generation
Source: Chemistry. 2026 Mar 4;32(19):e70843. doi: 10.1002/chem.70843 (PMC13206396; doi:10.1002/chem.70843)
Supplement: Supplementary file 1 — The authors have cited additional references within the Supporting Information [S1–S3]. [file CHEM-32-e70843-s001.pdf]

Supporting Information

Room-Temperature Helix-to-Disc Conversion of Thia[6]helicene *S,S*-Dioxides to Coronene via In Situ Phenoxide Anion Generation

Kaito Seino and Takashi Murase*

Faculty of Science, Yamagata University,
1-4-12 Kojirakawa-machi, Yamagata, Yamagata 990-8560, Japan

*E-mail: tmurase@sci.kj.yamagata-u.ac.jp

Contents

1. Materials and General Methods **3**

2. Synthesis and Physical Properties **4**

2-1. Synthesis of **3** 4

2-2. Synthesis of **4** 4

2-3. Synthesis of **5** 5

2-4. Synthesis of **6** 6

2-5. Synthesis of thia[6]helicene **7** 7

2-6. Synthesis of thia[6]helicene *S*,*S*-dioxide **1d** 7

2-7. Transformation of thia[6]helicene *S*,*S*-dioxide **1d** into **coronene** (Figure S1) 9

2-8. Synthesis of thia[6]helicene *S*,*S*-dioxide **1c** (Figure S2) 10

2-9. Heating of thia[6]helicene *S*,*S*-dioxide **1c** 11

2-10. Synthesis of oxa-Michael adduct **1c'** 12

3. DFT Calculations **14**

Computational details 14

Frontier molecular orbitals and their energies for **1b** and **1c** (Figure S3) 17

Optimized Cartesian coordinates 18

Absolute energy contributions for key transformations (Tables S1–S3) 27

4. NMR Spectra **29**

Compound **4** (Figures S4–S7) 29

Compound **5** (Figures S8–S11) 31

Compound (*Z*)-**6** (Figures S12–S15) 33

Compound (*E*)-**6** (Figures S16–S19) 35

Thia[6]helicene **7** (Figures S20–S23) 37

Thia[6]helicene *S*,*S*-dioxide **1d** (Figures S24–S27) 39

Thia[6]helicene *S*,*S*-dioxide **1c** (Figures S28–S37) 41

Oxa-Michael adduct **1c'**(α) (Figures S38–S50) 46

Oxa-Michael adduct **1c'**(β) (Figures S51–S53) 53

Assignment of the relative stereochemistry of **1c'**(α) and **1c'**(β) (Figure S54) 54

5. MS Spectra **55**

6. References **64**

1. Materials and General Methods

^{1}H (500 MHz), ^{13}C (125 MHz), and other 2D NMR spectra were recorded at 298 K on a JEOL JNM-ECZ-500R spectrometer, with chemical shifts referenced to tetramethylsilane (TMS, $\delta = 0$ ppm) in $CDCl_3$ and to the residual DMSO-d_6 signals ($\delta_H = 2.50$ ppm, $\delta_C = 39.52$ ppm) in DMSO-d_6. ESI-TOF-MS spectra were recorded on a Bruker micrOTOF II spectrometer. Melting points were determined with a Barnstead MEL-TEMP 1001D melting point apparatus. Photoirradiation was carried out with a high-pressure mercury lamp (HB400X-15 400 W, SEN LIGHTS CORP) in a quartz water-cooling jacket, which was placed at the center of a reaction vessel (500 or 1000 mL) for internal irradiation. TLC analyses were performed on glass plates precoated with silica gel 60 F_{254} (AS ONE, G2020). Solvents and reagents were purchased from commercial suppliers (TCI, FUJIFILM Wako Pure Chemical, Kanto Chemical, Nacalai Tesque, Sigma-Aldrich, Angene) and used without further purification.

2. Synthesis and Physical Properties

2-1. Synthesis of **3**

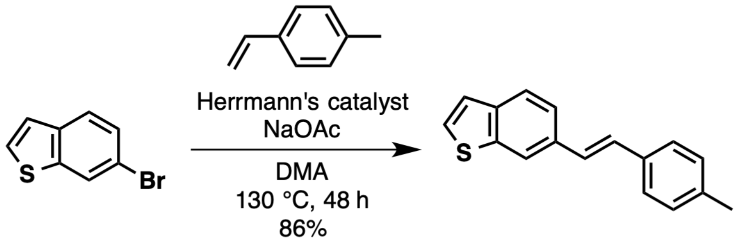

2 **3**

6-Bromobenzothiophene (**2**; 3.24 g, 15.2 mmol), sodium acetate (1.50 g, 18.3 mmol), and *trans*-bis(acetato)bis[*o*-(di-*o*-tolylphosphino)benzyl]dipalladium(II) (Herrmann's catalyst; 285 mg, 0.304 mmol) were dissolved in dry DMA (48 mL), which had been purged with nitrogen for 15 min. After the solution was heated to 100 °C, 4-methylstylene (6.0 mL, 46 mmol) was added. The resulting mixture was stirred at 130 °C for 48 h under a nitrogen atmosphere. The reaction mixture was cooled to room temperature, poured into water, and the resulting precipitate was collected by filtration. The crude product was triturated with EtOH (30 mL), purified by column chromatography on silica gel (hexane/toluene = 10:1 to toluene only), and then triturated again with EtOH (2 × 20 mL) to give compound **3** as a pale yellow solid (3.26 g, 13.0 mmol, 86%). The ^{1}H NMR data were identical to those reported in the literature.[S1] ^{1}H NMR (500 MHz, $CDCl_3$, 298 K) δ = 7.97 (s, 1H), 7.79 (d, 1H, *J* = 8.1 Hz), 7.57 (dd, 1H, *J* = 8.4 Hz, 1.5 Hz), 7.44 (d, 2H, *J* = 8.1 Hz), 7.42 (d, 1H, *J* = 5.4 Hz), 7.31 (dd, 1H, *J* = 5.4 Hz, 0.6 Hz), 7.18 (d, 2H, *J* = 7.8 Hz), 7.16 (s, 2H; *appeared as singlet due to overlapped coupled doublets*), 2.37 (s, 3H).

2-2. Synthesis of **4**

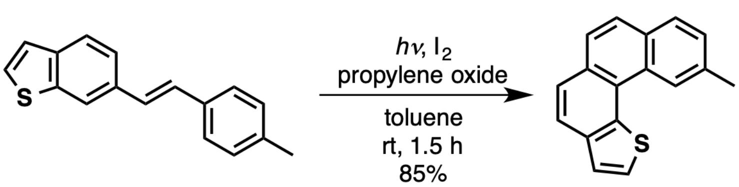

3 **4**

A mixture of compound **3** (641 mg, 2.56 mmol), I_2 (780 mg, 3.07 mmol), and propylene oxide (3.6 mL, 51 mmol) in toluene (1 L) was purged with nitrogen gas for 15 min. The resulting solution was irradiated with a high-pressure Hg lamp at room temperature for 1.5 h. The reaction mixture was concentrated *in vacuo*, and the residue was dissolved in $CHCl_3$. The organic layer was washed with aqueous $Na_2S_2O_3$ solution, dried over anhydrous Na_2SO_4, filtered, and concentrated *in vacuo*. The residue was purified by column chromatography on silica gel (hexane) to give compound **4** (538 mg, 2.16 mmol, 85%) as a white solid.

4: TLC (hexane) R_f = 0.31; M.p. 74.0–76.0 °C; ^{1}H NMR (500 MHz, $CDCl_3$, 298 K) δ = 9.04 (s, 1H), 8.04 (d, 1H, *J* = 8.4 Hz), 7.92 (d, 1H, *J* = 8.4 Hz), 7.89 (d, 1H, *J* = 8.1 Hz), 7.85 (d, 1H, *J* = 8.7 Hz), 7.81 (d, 1H, *J* = 8.7 Hz), 7.68 (d, 1H, *J* = 5.4 Hz), 7.61 (d, 1H, *J* = 5.4 Hz), 7.52 (dd, 1H, *J* = 8.0 Hz, 1.1 Hz), 2.75 (s, 3H); $^{13}C\{^1H\}$ NMR (125 MHz, $CDCl_3$, 298 K) δ = 139.2 (C), 136.7 (C), 134.8 (C), 130.84 (C), 130.80 (C), 129.5 (C), 128.8 (CH), 127.9 (CH), 126.8 (CH), 126.5 (CH), 126.2 (CH), 126.1 (C), 125.8 (CH), 125.7 (CH), 124.6 (CH), 122.7 (CH), 22.5 (CH_3); HR-MS (ESI) *m/z* calcd for $[M+Na]^+$: 271.0552, found: 271.0545.

2-3. Synthesis of **5**

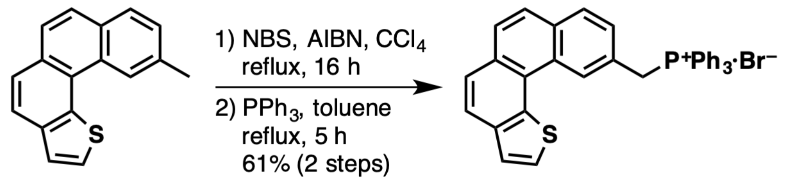

A mixture of compound **4** (0.343 g, 1.38 mmol), NBS (0.248 g, 1.39 mmol), and AIBN (23.3 mg, 0.142 mmol) in CCl_4 (21 mL) was refluxed for 16 h. The resulting precipitate was removed by filtration and washed with CCl_4. The combined filtrate was then concentrated *in vacuo*. The residue was dissolved in $CHCl_3$, and the organic layer was washed with aqueous $NaHCO_3$ and $Na_2S_2O_3$ solutions, dried over Na_2SO_4, filtered, and concentrated *in vacuo*. The resulting residue was passed through a short silica gel column (hexane/$CHCl_3$ = 10:1) to give a crude monobrominated compound (0.371 g, estimated purity: 70%). This material, which contained small amounts of unreacted **4** and a dibrominated byproduct, was used in the next step without further purification.

The crude monobrominated product was redissolved in toluene (10 mL), followed by the addition of PPh_3 (0.306 g, 1.17 mmol), and the mixture was stirred at reflux for 5 h. After cooling to room temperature, the resulting precipitate was collected by filtration and washed with cold toluene to give **5** as a pale orange solid (0.496 g, 0.842 mmol, 61% over two steps).

5: M.p. > 292 °C (decomp., brown solid); ^{1}H NMR (500 MHz, $CDCl_3$, 298 K) δ = 8.57 (d, 1H, $^4J_{HP}$ = 1.8 Hz), 7.97 (d, 1H, *J* = 8.4 Hz), 7.79–7.75 (m, 8H), 7.71 (td, 3H, *J* = 7.5 Hz, 1.8 Hz), 7.66 (d, 1H, *J* = 8.1 Hz), 7.60–7.55 (m, 9H), 7.50 (d, 1H, *J* = 5.7 Hz), 5.71 (d, 2H, $^2J_{HP}$ = 14.4 Hz); $^{13}C\{^1H\}$ NMR (125 MHz, $CDCl_3$, 298 K) δ = 139.3 (C), 134.9 (3CH, d, J_{CP} = 3.0 Hz), 134.5 (6CH, d, J_{CP} = 9.6 Hz), 134.4 (C), 132.2 (C, d, J_{CP} = 3.6 Hz), 130.6 (C), 130.3 (6CH, d, J_{CP} = 12.6 Hz), 129.7 (CH, d, J_{CP} = 4.8 Hz), 129.6 (CH, d, J_{CP} = 3.0 Hz), 128.9 (C, d, J_{CP} = 3.0 Hz), 128.5 (CH), 128.1 (CH, d, J_{CP} = 6.6 Hz), 126.2 (CH, d, J_{CP} = 1.8 Hz), 126.1 (CH), 126.0 (CH), 125.7 (C, d, J_{CP} = 9.0 Hz), 125.5 (C), 124.3 (CH), 123.1 (CH), 117.8 (3C, d, J_{CP} = 85.8 Hz), 31.8 (CH_2, d, J_{CP} = 46.2 Hz); HR-MS (ESI) *m/z* calcd for $[M–Br]^+$: 509.1487, found: 509.1475.

2-4. Synthesis of **6**

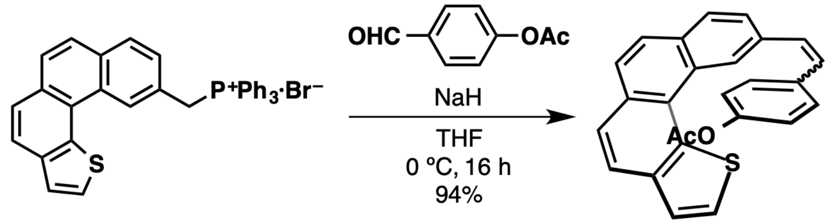

5 **6**

A mixture of compound **5** (102 mg, 0.172 mmol) and NaH (6.4 mg, 2.66 mmol; 55% dispersion in paraffin liquid) in dry THF (2.6 mL) was stirred at 0 °C for 1 h under a nitrogen atmosphere. 4-Acetoxybenzaldehyde (72.5 μL, 0.521 mmol) was added dropwise, and the resulting solution was stirred at 0 °C for 16 h under a nitrogen atmosphere. The reaction mixture was quenched with saturated aqueous NH_4Cl solution, concentrated *in vacuo*, and the residue was dissolved in $CHCl_3$. The organic layer was washed with water, dried over anhydrous Na_2SO_4, filtered, and concentrated *in vacuo*. The crude product was purified by column chromatography on silica gel (hexane/$CHCl_3$ = 1:1) to give compound **6** (*E*/*Z* = 71:29; 64.0 mg, 0.162 mmol, 94%) as a pale yellow solid.

(*Z*)-**6**: yellow oil; TLC (hexane/$CHCl_3$ = 1:2) R_f = 0.47; ^{1}H NMR (500 MHz, $CDCl_3$, 298 K) δ = 9.23 (s, 1H), 8.03 (d, 1H, *J* = 8.1 Hz), 7.872 (d, 1H, *J* = 8.7 Hz), 7.866 (d, 2H, *J* = 8.4 Hz), 7.78 (d, 1H, *J* = 8.7 Hz), 7.62 (d, 1H, *J* = 5.4 Hz), 7.57 (dd, 1H, *J* = 8.5 Hz, 1.1 Hz), 7.56 (d, 1H, *J* = 5.7 Hz), 7.40 (d, 2H, *J* = 8.4 Hz), 6.96 (d, 2H, *J* = 8.4 Hz), 6.95 (d, 1H, *J* = 12.2 Hz), 6.76 (d, 1H, *J* = 12.1 Hz), 2.25 (s, 3H); ^{13}C{^{1}H} NMR (125 MHz, $CDCl_3$, 298 K) δ = 169.3 (CO), 149.8 (C), 139.4 (C), 135.6 (C), 134.92 (C), 134.89 (C), 132.0 (C), 130.9 (CH), 130.7 (C), 130.2 (2CH), 129.9 (CH), 129.4 (C), 128.9 (CH), 127.9 (CH), 127.1 (CH), 126.5 (CH), 126.34 (2CH), 126.32 (C), 126.2 (CH), 124.3 (CH), 122.9 (CH), 121.6 (2CH), 21.2 (CH_3); HR-MS (ESI) *m/z* calcd for $[M+Na]^+$: 417.0920, found: 417.0924.

(*E*)-**6**: pale yellow solid; TLC (hexane/$CHCl_3$ = 1:2) R_f = 0.40; M.p. 173.0–174.5 °C; ^{1}H NMR (500 MHz, $CDCl_3$, 298 K) δ = 9.30 (s, 1H), 8.08 (d, 1H, *J* = 8.4 Hz), 8.00 (d, 1H, *J* = 8.1 Hz), 7.91 (d, 1H, *J* = 8.1 Hz), 7.90 (d, 2H, *J* = 8.4 Hz; *overlapped doublets*), 7.83 (d, 1H, *J* = 8.4 Hz), 7.73 (d, 1H, *J* = 5.4 Hz), 7.66 (d, 2H, *J* = 8.4 Hz), 7.64 (d, 1H, *J* = 5.4 Hz), 7.44 (d, 1H, *J* = 16.2 Hz), 7.39 (d, 1H, *J* = 16.2 Hz), 7.16 (d, 2H, *J* = 8.4 Hz), 2.34 (s, 3H); ^{13}C{^{1}H} NMR (125 MHz, $CDCl_3$, 298 K) δ = 169.5 (CO), 150.2 (C), 139.5 (C), 135.9 (C), 135.2 (C), 134.8 (C), 132.5 (C), 131.0 (C), 129.8 (C), 129.6 (CH), 129.4 (CH), 128.5 (CH), 127.9 (CH), 127.6 (2CH), 126.40 (CH), 126.38 (C), 126.3 (CH), 126.0 (CH), 124.8 (CH), 124.7 (CH), 124.0 (CH), 123.0 (CH), 121.9 (2CH), 21.2 (CH_3); HR-MS (ESI) *m/z* calcd for $[M+Na]^+$: 417.0920, found: 417.0913.

2-5. Synthesis of thia[6]helicene **7**

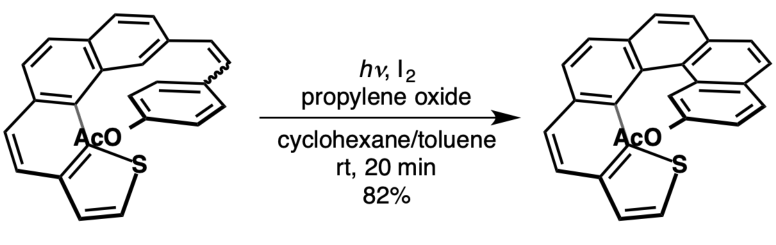

6 7

A mixture of compound **6** (237 mg, 0.601 mmol), I_2 (168 mg, 0.663 mmol), and propylene oxide (1.3 mL, 19 mmol) in cyclohexane/toluene (1,000 mL, 9:1 v/v) was purged with nitrogen gas for 15 min. The resulting solution was irradiated with a high-pressure Hg lamp at room temperature for 20 min. The reaction mixture was concentrated *in vacuo*, and the residue was dissolved in $CHCl_3$. The organic layer was washed with aqueous $Na_2S_2O_3$ solution, dried over anhydrous Na_2SO_4, filtered, and concentrated *in vacuo*. The residue was purified by column chromatography on silica gel (hexane/$CHCl_3$ = 1:1) to give thia[6]helicene **7** (192 mg, 0.490 mmol, 82%) as a yellow solid.

7: TLC (hexane/$CHCl_3$ = 1:2) R_f = 0.36; M.p. 85.0–87.0 °C; ^{1}H NMR (500 MHz, $CDCl_3$, 298 K) δ = 8.06 (d, 1H, J = 8.4 Hz), 8.02 (d, 1H, J = 8.4 Hz), 8.00 (d, 1H, J = 8.7 Hz), 7.99 (d, 1H, J = 9.0 Hz), 7.98 (d, 1H, J = 8.1 Hz), 7.973 (d, 1H, J = 8.4 Hz), 7.967 (d, 1H, J = 8.4 Hz), 7.96 (d, 1H, J = 9.2 Hz), 7.90 (d, 1H, J = 8.4 Hz), 7.61 (d, 1H, J = 2.4 Hz), 7.29 (d, 1H, J = 5.4 Hz), 7.140 (dd, 1H, J = 8.4 Hz, 2.1 Hz), 7.135 (d, 1H, J = 5.4 Hz), 2.03 (s, 3H); ^{13}C{^{1}H} NMR (125 MHz, $CDCl_3$, 298 K) δ = 169.3 (CO), 148.4 (C), 138.5 (C), 137.1 (C), 132.9 (C), 132.0 (C), 131.6 (C), 130.8 (C), 129.9 (C), 129.0 (CH), 128.2 (CH), 127.5 (CH), 127.2 (CH), 127.1 (C), 127.0 (CH), 126.4 (CH), 125.83 (CH), 125.79 (C), 125.2 (CH), 124.8 (CH), 124.0 (C), 122.93 (CH), 122.88 (CH), 120.8 (CH), 118.6 (CH), 20.9 (CH_3); HR-MS (ESI) m/z calcd for $[M+Na]^+$: 415.0763, found: 415.0759.

2-6. Synthesis of thia[6]helicene *S*,*S*-dioxide **1d**

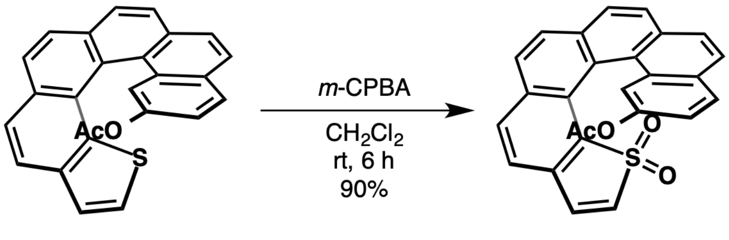

7 1d

Thia[6]helicene **7** (82.3 mg, 0.210 mmol) was dissolved in CH_2Cl_2 (15 mL). To this solution, *m*-CPBA (145 mg, 70% wt, 0.841 mmol) was slowly added, and the resulting solution was stirred at room temperature for 6 h. The reaction mixture was washed with triethylamine (2 × 1 mL) and water and then extracted with CH_2Cl_2. The combined organic layers were washed with water, dried over anhydrous Na_2SO_4, filtered, and concentrated *in vacuo*. The crude residue was purified by

column chromatography on silica gel ($CHCl_3$/MeOH = 500:1) to give thia[6]helicene *S*,*S*-dioxide **1d** (79.7 mg, 0.188 mmol, 90%) as an orange-yellow solid.

1d: TLC (hexane/EtOAc = 1:2) R_f = 0.54; M.p. > 231 °C (decomp., yellowish-brown solid); ^{1}H NMR (500 MHz, $CDCl_3$, 298 K) δ = 8.18 (d, 1H, *J* = 8.1 Hz), 8.05 (d, 1H, *J* = 8.1 Hz), 7.99 (d, 1H, *J* = 8.4 Hz), 7.98 (d, 1H, *J* = 8.4 Hz), 7.91 (d, 1H, *J* = 8.3 Hz), 7.90 (s, 2H; *appeared as singlet due to overlapped coupled doublets*), 7.86 (d, 1H, *J* = 8.1 Hz), 7.71 (d, 1H, *J* = 2.1 Hz), 7.51 (d, 1H, *J* = 8.1 Hz), 7.20 (dd, 1H, *J* = 8.7 Hz, 2.4 Hz), 7.17 (d, 1H, *J* = 6.9 Hz), 6.35 (d, 1H, *J* = 6.9 Hz), 2.14 (s, 3H); ^{13}C{^{1}H} NMR (125 MHz, $CDCl_3$, 298 K) δ = 169.3 (CO), 148.5 (C), 135.1 (C), 134.9 (C), 133.4 (CH), 133.3 (C), 132.7 (C), 132.4 (C), 131.6 (C), 131.5 (CH), 131.3 (CH), 131.0 (C), 129.9 (CH), 129.7 (CH), 128.3 (CH), 128.1 (CH), 127.9 (C), 126.8 (CH), 126.4 (C), 125.9 (CH), 124.7 (CH), 123.7 (C), 123.0 (CH), 120.5 (CH), 118.1 (CH), 21.1 (CH_3); HR-MS (ESI) *m/z* calcd for $[M+Na]^+$: 447.0662, found: 447.0666.

2-7. Transformation of thia[6]helicene *S*,*S*-dioxide **1d** into **coronene**

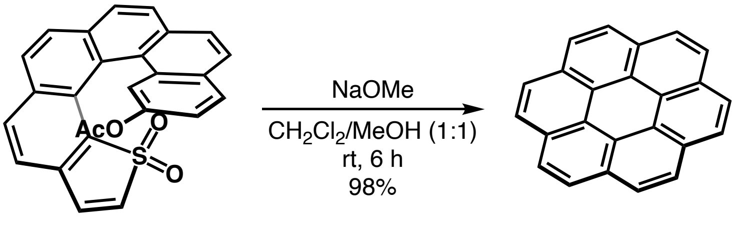

Thia[6]helicene *S*,*S*-dioxide **1d** (33.3 mg, 0.0785 mmol) was dissolved in CH_2Cl_2/MeOH (1.6 mL, 1:1 v/v). Then, a solution of NaOMe in MeOH (1.0 M, 0.235 mL, 0.235 mmol) was added dropwise, and the resulting solution (overall CH_2Cl_2/MeOH ≈ 1:1.3, v/v) was stirred at room temperature for 6 h. After TLC analysis confirmed completion of the reaction with a single new spot corresponding to **coronene**, the resulting precipitate was collected by filtration and washed with MeOH to give **coronene** (15.1 mg, 0.0503 mmol, 64%). The filtrate was concentrated by rotary evaporation, and MeOH (2 mL) was added. The suspension was heated at 45 °C, and the resulting precipitate was collected by filtration and washed with cold MeOH to give **coronene** (5.1 mg, 0.0170 mmol, 22%). The mother liquor was again concentrated by rotary evaporation, MeOH (0.5 mL) was added, and the suspension was reheated at 45 °C. The resulting precipitate was collected by filtration and washed with cold MeOH to give **coronene** (2.8 mg, 9.3×10^{-3} mmol, 12%). Thus, the total yield of coronene was 98% (23.0 mg, 0.0766 mmol). All isolated samples of coronene were confirmed to be analytically pure by ^{1}H NMR spectroscopy in $CDCl_3$, showing a single resonance at δ 8.94 ppm (Figure S1).

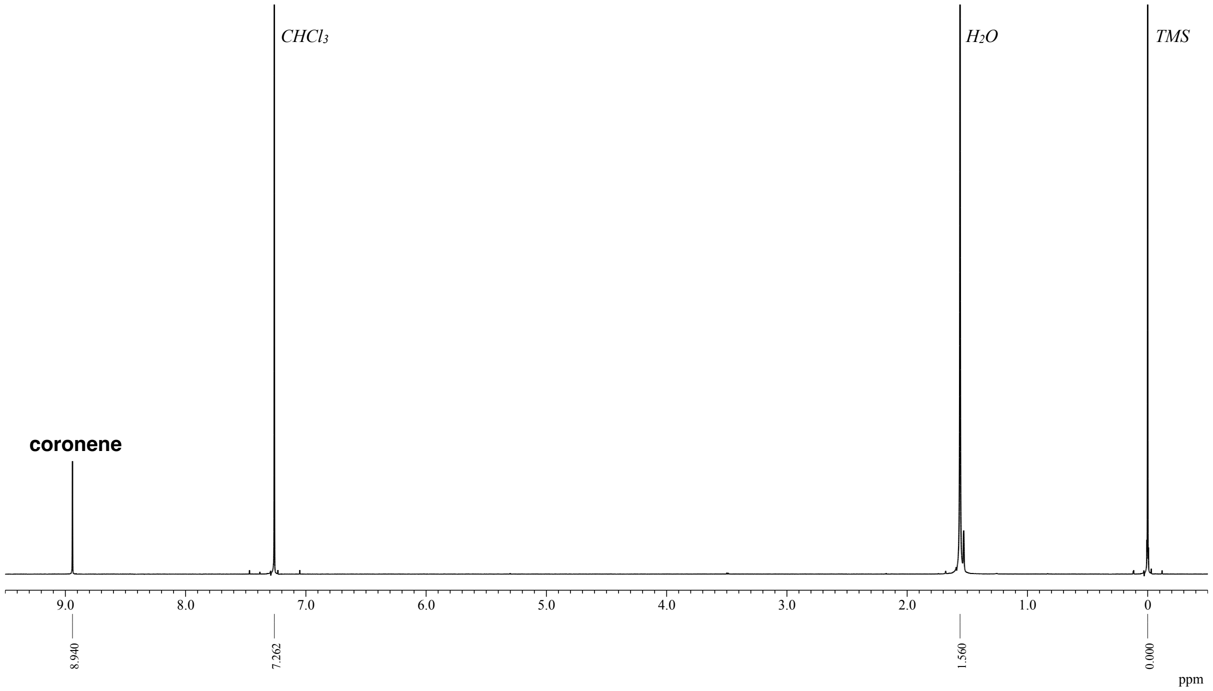

Figure S1. ^{1}H NMR spectrum (500 MHz, $CDCl_3$, 298 K) of coronene isolated as the precipitate after the helix-to-disc conversion of **1d** to **coronene** in CH_2Cl_2/MeOH (1:1).

2-8. Synthesis of thia[6]helicene *S*,*S*-dioxide **1c**

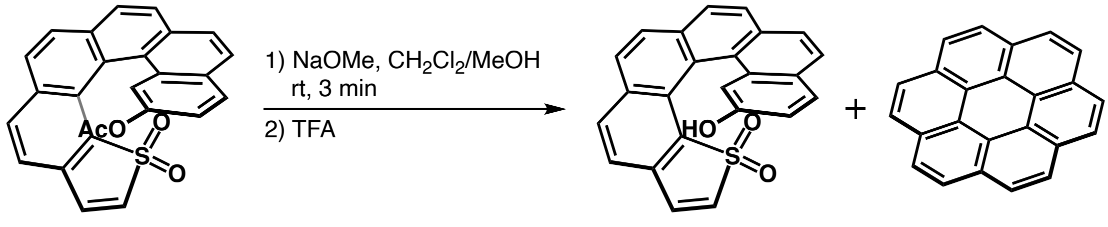

Thia[6]helicene *S*,*S*-dioxide **1d** (70.7 mg, 0.167 mmol) was dissolved in CH_2Cl_2/MeOH (3.4 mL, 1:1 v/v). To this solution, a solution of NaOMe in MeOH (1.0 M, 0.50 mL, 0.50 mmol) was added dropwise, and the resulting solution (overall CH_2Cl_2/MeOH ≈ 1:1.3, v/v) was stirred at room temperature for 3 min. TFA (64 μL, 0.83 mmol) was then added to rapidly quench the reaction. The solvent was removed under reduced pressure, and MeOH (0.50 mL) was added. The resulting precipitate was collected by filtration and washed with cold MeOH to give a mixture of thia[6]helicene *S*,*S*-dioxide **1c** and **coronene** (45.9 mg, **1c**:**coronene** = 1:0.038, see Figure S2a for the ^{1}H NMR spectrum). The filtrate was concentrated by rotary evaporation to give a crude residue (**1c**:**coronene** = 1:0.020, see Figure S2b for the ^{1}H NMR spectrum), which was then extracted with EtOAc. The combined organic layers were washed with water, dried over anhydrous Na_2SO_4, filtered, and concentrated *in vacuo*. The residue (9.4 mg) was purified by column chromatography on silica gel (hexane/EtOAc = 2:3) to give a mixture of **1c** and **coronene** (5.1 mg, **1c**:**coronene** = 1:0.0034, see Figure S2c for the ^{1}H NMR spectrum). Total yields: 78% for **1c** (49.7 mg, 0.130 mmol) and 3% for **coronene** (1.3 mg, 4.3×10^{-3} mmol).

Pure **1c**, free of **coronene**, could finally be obtained when the reaction was performed at 0 °C, albeit with difficulty. Isolated **1c** shows poor solubility in chlorinated solvents (CH_2Cl_2 and $CHCl_3$) and aromatic solvents (toluene and mesitylene) but readily dissolves in polar aprotic solvents (acetone and DMSO). All ^{1}H and ^{13}C NMR signals of **1c** were fully assigned using a combination of 1D and 2D NMR experiments (COSY, HMQC, and HMBC; see Figures S28–S37).

1c: deep yellow solid; TLC (hexane/EtOAc = 1:2) R_f = 0.43; M.p. > 195 °C (decomp., yellowish-brown solid); ^{1}H NMR (500 MHz, DMSO-d_6, 298 K) δ = 9.29 (s, 1H), 8.43 (d, 1H, J = 8.2 Hz), 8.10 (d, 2H, J = 8.2 Hz; *appeared as a single doublet due to two overlapped doublets*), 8.05 (d, 1H, J = 8.3 Hz), 7.96 (d, 1H, J = 8.2 Hz), 7.89 (d, 1H, J = 8.5 Hz), 7.80 (d, 1H, J = 8.2 Hz), 7.78 (d, 1H, J = 8.6 Hz), 7.75 (d, 1H, J = 8.6 Hz), 7.58 (d, 1H, J = 6.7 Hz), 7.21 (t, 1H, J = 2.2 Hz), 6.95 (td, 1H, J = 8.6 Hz, 2.2 Hz), 6.92 (dd, 1H, J = 6.7 Hz, 1.0 Hz); ^{13}C{^{1}H} NMR (125 MHz, DMSO-d_6, 298 K) δ = 155.5 (C), 134.9 (C), 134.4 (C), 133.7 (CH), 133.2 (C), 132.6 (C), 132.4 (CH), 132.1 (C), 131.7 (CH), 131.4 (C), 129.8 (CH), 129.7 (CH), 128.5 (CH), 127.7 (CH), 126.8 (C+C), 126.7 (CH), 125.43 (C), 125.37 (CH), 124.1 (CH), 123.3 (C), 121.3 (CH), 117.2 (CH), 109.0 (CH); HR-MS (ESI) *m/z* calcd for $[M+Na]^+$: 405.0556, found: 405.0554.

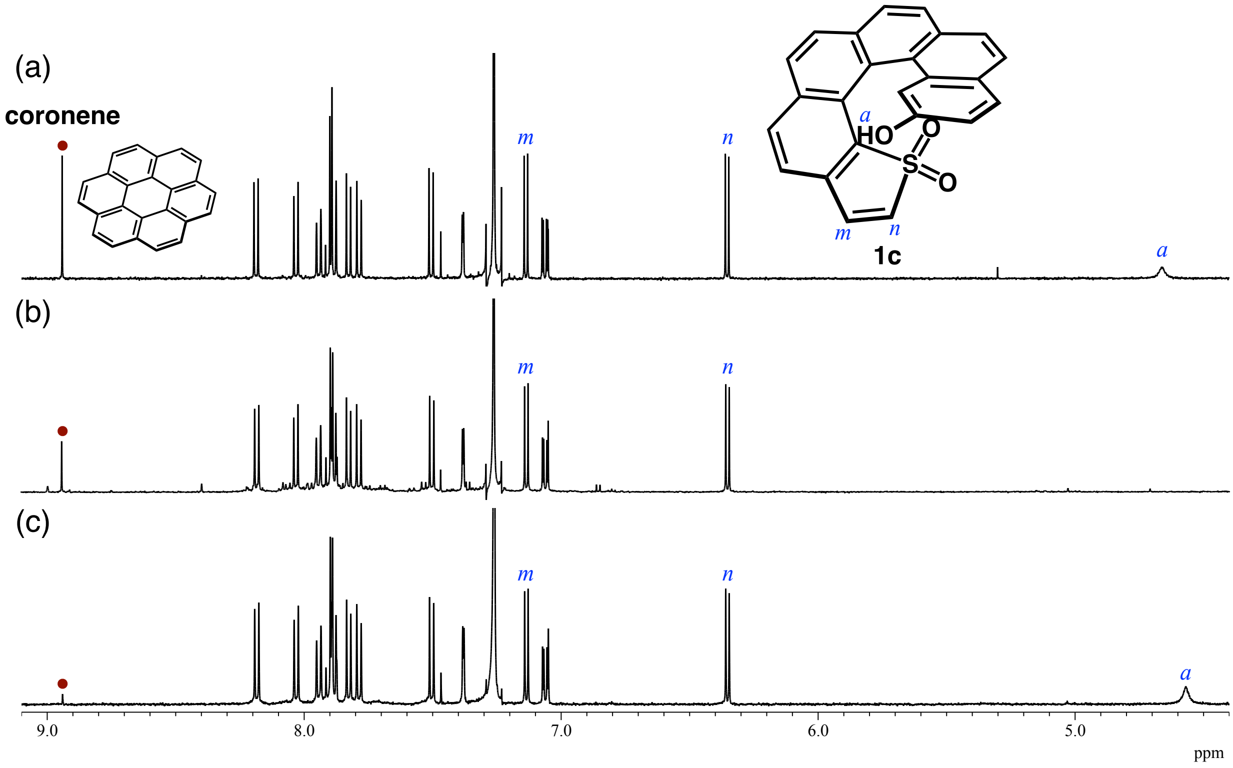

Figure S2. ^1H NMR spectra (500 MHz, $CDCl_3$, 298 K) of (a) the precipitate obtained after addition of MeOH (**1c**:**coronene** = 1:0.038), (b) the crude residue obtained after concentrating the filtrate (**1c**:**coronene** = 1:0.020), and (c) the chromatographic fraction (**1c**:**coronene** = 1:0.0034). Note that, because coronene is highly symmetric (12 equivalent aromatic protons), its signal appears relatively intense even though it is present only in small amounts in these samples.

2-9. Heating of thia[6]helicene *S*,*S*-dioxide **1c**

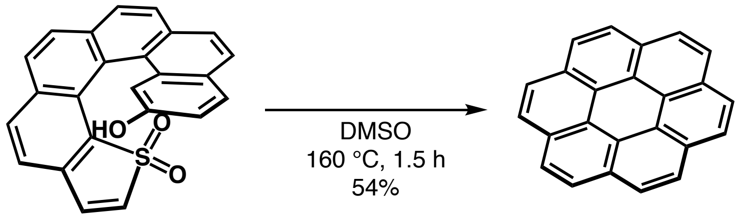

1c **coronene**

Thia[6]helicene *S*,*S*-dioxide **1c** (19.8 mg, 0.0518 mmol) was dissolved in dry DMSO (26 mL) and heated at 160 °C for 1.5 h. After TLC analysis confirmed completion of the reaction, water (60 mL) was added, and the resulting precipitate was collected by filtration and washed with water to give **coronene** (8.5 mg, 0.028 mmol, 54%).

2-10. Synthesis of oxa-Michael adduct **1c'**

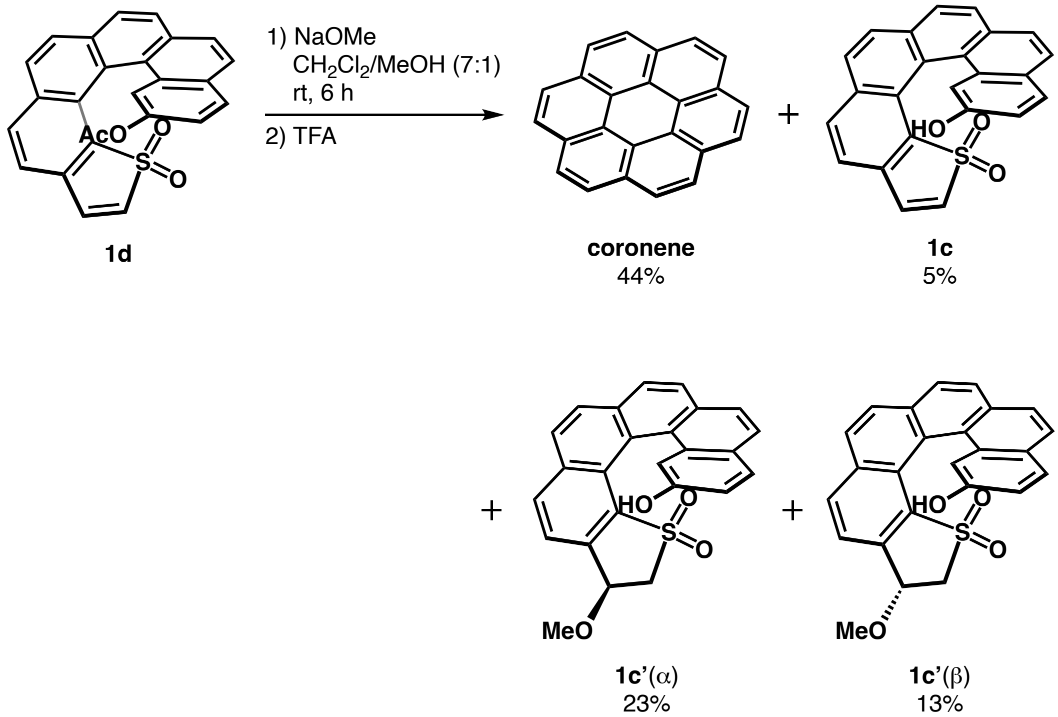

Thia[6]helicene *S*,*S*-dioxide **1d** (33.4 mg, 0.0787 mmol) was dissolved in CH_2Cl_2 (1.6 mL). To this solution, a solution of NaOMe in MeOH (1.0 M, 0.235 mL, 0.235 mmol) was added dropwise, and the resulting solution (overall CH_2Cl_2/MeOH = 7:1, v/v) was stirred at room temperature for 6 h. The reaction was quenched by addition of TFA (30 μL, 0.39 mmol), and the resulting precipitate was collected by filtration, washed with cold CH_2Cl_2, and triturated with MeOH to give **coronene** (6.1 mg, 0.0203 mmol, 26%). The combined CH_2Cl_2 filtrates were washed with water, dried over anhydrous Na_2SO_4, filtered, and concentrated *in vacuo*. The residue was purified by column chromatography on silica gel (hexane/EtOAc = 2:3) to give **coronene** (4.2 mg, 0.0140 mmol, 18%), **1c'**(α) (7.5 mg, 0.0181 mmol, 23%), and a mixture of **1c** and **1c'**(β) (5.7 mg, **1c**:**1c'**(β) = 0.423:1). Overall, **coronene** was obtained in 44% yield (10.3 mg, 0.0343 mmol). The yields of **1c** (5%) and **1c'**(β) (13%) were estimated from the isolated mixture using the above ratio. All ^1H and ^{13}C NMR signals of **1c'**(α) were fully assigned using a combination of 1D and 2D NMR experiments (COSY, HMQC, and HMBC; see Figures S38–S50).

Because chromatographic separation of **1c** from **1c'**(β) was difficult, the isolated mixture was treated with NaOMe/MeOH (1.0 M) in CH_2Cl_2/MeOH (1:1) to convert **1c** into **coronene**. This operation enabled enrichment of **1c'**(β); however, the recovered fraction still contained a small amount of **coronene** as well as minor impurities.

1c'(α): pale brown solid; TLC (hexane/EtOAc = 1:2) R_f = 0.20; M.p. > 168 °C (decomp., black solid); ^1H NMR (500 MHz, $CDCl_3$, 298 K) δ = 8.21 (d, 1H, *J* = 8.0 Hz), 8.02 (d, 1H, *J* = 8.2 Hz), 7.95 (d, 1H, *J* = 8.5 Hz), 7.93 (d, 1H, *J* = 9.2 Hz), 7.89 (d, 1H, *J* = 8.3 Hz), 7.87 (d, 1H, *J* = 8.6 Hz), 7.84 (d, 1H, *J* = 8.2 Hz), 7.77 (d, 1H, *J* = 8.6 Hz), 7.63 (d, 1H, *J* = 8.2 Hz), 7.32 (d, 1H, *J* = 2.4 Hz),

7.09 (dd, 1H, J = 8.6 Hz, 2.4 Hz), 4.98 (br s, 1H), 4.88 (dd, 1H, J = 6.0 Hz, 1.7 Hz), 3.57 (s, 3H), 3.28 (dd, 1H, J = 13.2 Hz, 1.7 Hz), 3.04 (dd, 1H, J = 13.2 Hz, 6.0 Hz); $^{13}C\{^1H\}$ NMR (125 MHz, $CDCl_3$, 298 K) δ = 153.8 (C), 138.4 (C), 136.7 (C), 133.6 (C), 133.4 (CH), 133.1 (C), 132.9 (C), 132.2 (C), 130.2 (CH), 129.9 (CH), 128.60 (CH), 128.56 (CH), 128.3 (C), 127.6 (C), 126.2 (CH), 125.6 (C), 125.5 (CH), 125.0 (CH), 123.6 (C), 122.7 (CH), 117.1 (CH), 110.6 (CH), 74.3 (CH), 57.6 (CH_2), 56.9 (OCH_3); HR-MS (ESI) *m/z* calcd for $[M+Na]^+$: 437.0818, found: 437.0827.

1c'(β): pale brown solid; TLC (hexane/EtOAc = 1:2) R_f = 0.43; ^1H NMR (500 MHz, $CDCl_3$, 298 K) δ = 8.22 (d, 1H, J = 8.2 Hz), 8.00 (d, 1H, J = 8.2 Hz), 7.93 (d, 2H, J = 8.3 Hz; *overlapped doublets*), 7.91 (d, 1H, J = 8.6 Hz), 7.88 (d, 1H, J = 8.6 Hz), 7.83 (d, 1H, J = 7.9 Hz), 7.82 (d, 1H, J = 8.0 Hz), 7.78 (d, 1H, J = 8.5 Hz), 7.29 (d, 1H, J = 2.4 Hz), 7.08 (dd, 1H, J = 8.5 Hz, 2.5 Hz), 5.38 (dd, 1H, J = 8.7 Hz, 5.9 Hz), 4.59 (br s, 1H), 3.57 (dd, 1H, J = 11.3 Hz, 5.9 Hz), 3.49 (s, 3H), 2.68 (dd, 1H, J = 11.3 Hz, 8.7 Hz); HR-MS (ESI) *m/z* calcd for $[M+Na]^+$: 437.0818, found: 437.0821.

3. DFT Calculations

Computational details

- **Software and levels**

All DFT calculations were performed with Spartan'24 (Wavefunction, Inc., Irvine, CA, USA). The M06-2X density functional was employed throughout, using 6-31+G(d,p) for geometry optimizations and harmonic frequency analyses, and 6-311+G(2d,p) for single-point electronic energies. Frontier molecular orbitals (HOMO and LUMO) were evaluated at the same level as the geometry optimizations; orbital isosurfaces were visualized at an isovalue of 0.07 a.u. The M06-2X density functional has proved reliable for the thermochemistry of cycloaddition processes, including barrier heights and reaction free energies.[S2]

- **Numerical settings and integration grid**

Self-consistent field (SCF) calculations used a maximum of 200 cycles and an energy convergence threshold of 1×10^{-8} hartree (SCFCYCLE = 200 and SCFTOLERANCE = 8). Numerical integration used 100 radial shells and 434 angular Lebedev points (VERYBIGGRID).

- **Solvation models and workflow**

Geometry optimizations and frequency analyses were performed in a continuum dielectric, CPCM(solvent). Single-point electronic energies were then refined with SMD(solvent) on the CPCM-optimized geometries. Frequencies were evaluated with CPCM rather than SMD because thermal corrections depend mainly on the vibrational spectrum and are only weakly sensitive to the specific continuum model. CPCM frequencies are also more stable and efficient in this program. SMD was used at the single-point stage to capture bulk-solvation contributions to the electronic energy with higher fidelity. Minima were verified by frequency analysis to have no imaginary frequencies. Transition states exhibited exactly one imaginary frequency along the reaction coordinate. For **TS15**, a small residual imaginary mode (ν = −32.90 cm^{-1}) orthogonal to the reaction coordinate was observed and inverted to the corresponding positive value (see **Thermochemistry conventions**).

- **Assembly of Gibbs free energies and temperatures**

Final Gibbs free energies were assembled as:

$$G_{\text{final}} = E_{\text{elec, SMD}} + G_{\text{corr, CPCM}}$$

where $E_{\text{elec, SMD}}$ is the single-point electronic energy computed with SMD on the CPCM-optimized geometry. $G_{\text{corr, CPCM}}$ is the Gibbs free energy correction taken from the CPCM frequency analysis. This assembly separates the bulk-solvation effect in $E_{\text{elec, SMD}}$ from the thermal correction obtained

at the level where the Hessian was computed. It avoids mixing frequency information across models and yields a consistent definition of G for all species.

The thermal contribution is defined as:

$$G_{\mathrm{corr,\ CPCM}} = H_{\mathrm{corr,\ CPCM}} - T{\cdot}S_{\mathrm{CPCM}}$$

where

$$H_{\mathrm{corr,\ CPCM}} = ZPE + H_{\mathrm{thermal,\ CPCM}}$$

ZPE is the zero-point vibrational energy. $H_{\mathrm{thermal,\ CPCM}}$ is the temperature-dependent enthalpy increment (translational + rotational + vibrational + PV) excluding ZPE.

In the CPCM model, the Gibbs free energy is defined as:

$$G_{\mathrm{CPCM}} = H_{\mathrm{CPCM}} - T{\cdot}S_{\mathrm{CPCM}} = (E_{\mathrm{elec,\ CPCM}} + H_{\mathrm{corr,\ CPCM}}) - T{\cdot}S_{\mathrm{CPCM}}$$

$$= E_{\mathrm{elec,\ CPCM}} + G_{\mathrm{corr,\ CPCM}}$$

Accordingly, the correction can also be written as:

$$G_{\mathrm{corr,\ CPCM}} = G_{\mathrm{CPCM}} - E_{\mathrm{elec,\ CPCM}}$$

Unless stated otherwise, thermochemistry is reported at 298.15 K and 1 atm. To compare with the experiment on **1c** conducted at 160 °C (T = 433.15 K), Gibbs free energies are additionally reported for the DMSO case using the same procedure.

- **Thermochemistry conventions (low-frequency handling and TS treatment)**

Low-frequency thermochemistry used Spartan's default clamping (CLAMPTHERMO, $x = 1/2$), i.e., per vibrational mode $H_{\mathrm{vib}} \leq 1/2RT$ and $S_{\mathrm{vib}} \leq R$ (break near 260 $\mathrm{cm^{-1}}$ at 298 K). For transition states, the reaction-coordinate imaginary mode was excluded from the partition function (zero contribution). Spartan treats each imaginary vibrational mode as contributing $-1/2RT$ to G_{corr} (implemented as $ZPE = 0$, $H_{\mathrm{vib}} = 1/2RT$, $S_{\mathrm{vib}} = R$). Accordingly, we added $+1/2RT$ to $G_{\mathrm{corr,\ CPCM}}$ for each TS to restore the conventional zero contribution (298.15 K: +0.296 kcal $\mathrm{mol^{-1}}$; 433.15 K: +0.430 kcal $\mathrm{mol^{-1}}$). Residual small imaginary frequencies orthogonal to the reaction coordinate ($|\nu| \leq 50\ \mathrm{cm^{-1}}$) were inverted to the corresponding positive values prior to evaluating thermal contributions.[S3] In practice, the residual imaginary mode ($\nu = -32.90\ \mathrm{cm^{-1}}$) for **TS15** was inverted to +32.90 $\mathrm{cm^{-1}}$ and evaluated under the same clamping. The inversion increased $G_{\mathrm{corr,\ CPCM}}$ by 0.047 kcal $\mathrm{mol^{-1}}$ for that mode, consistent with $ZPE = 1/2R\theta_{\nu}$, where the vibrational temperature is $\theta_{\nu} = 1.43878\nu$ K (ν in $\mathrm{cm^{-1}}$).

- **Rationale for modeling the CH_2Cl_2/MeOH (1:1) medium as MeOH**

The transformation of **1b** into **coronene** was conducted experimentally in a 1:1 CH_2Cl_2/MeOH mixture, but computations modeled the medium as MeOH only. Because the key elementary steps

involve anionic species and specific hydrogen bonding, the protic component (MeOH) governs stabilization and proton-transfer thermodynamics. Standard continuum models do not robustly represent specific solvation in mixed solvents. Therefore, MeOH was adopted as a physically motivated proxy. Given the lower polarity and non-protic character of CH_2Cl_2, its contribution to local anion stabilization is expected to be secondary to that of MeOH.

- **Hydroxide treatment (effective free energy)**

To capture specific hydrogen bonding to hydroxide, an effective free energy was defined as:

$$G_{\mathrm{eff}}(\mathrm{HO}^-) = G(\mathrm{HO}^-\cdots\mathrm{MeOH}) - G(\mathrm{MeOH})$$

We did not model hydroxide as a bare anion because, in protic media, it is strongly and specifically solvated. The one-methanol cluster provides a physically motivated reference for the free energy of HO^- and avoids arbitrary counterion placement (see "**Ions and counterions**"). The $HO^-\cdots$MeOH cluster was optimized and analyzed under CPCM(MeOH), then refined with SMD(MeOH) single-point electronic energies. A representative optimized structure is provided in the **Optimized Cartesian coordinates** section.

- **Ions and counterions**

To avoid model-dependent contact or solvent-separated ion pairs, explicit metal cations (e.g., Na^+) were not included. Anionic intermediates and transition states were treated as bare anions in the continuum solvent, and hydroxide was treated using $G_{\mathrm{eff}}(\mathrm{HO}^-)$.

- **Standard state correction**

Reaction free energies assembled from individual G values were corrected to the 1 M solution standard state by adding +1.89 kcal mol^{-1} per net increase of one molecule (Δn = number of product molecules – number of reactant molecules). This standard-state correction follows $\Delta G^\circ_{1\mathrm{M}} - \Delta G^\circ_{1\mathrm{atm}} = RT\ln[(RT/p^\circ) \times (1\ \mathrm{mol\ L}^{-1})]$ and equals 1.89 kcal mol^{-1} per Δn at 298.15 K. Unless noted otherwise, reported ΔG values include this correction and the thermochemistry conventions above.

Frontier molecular orbitals

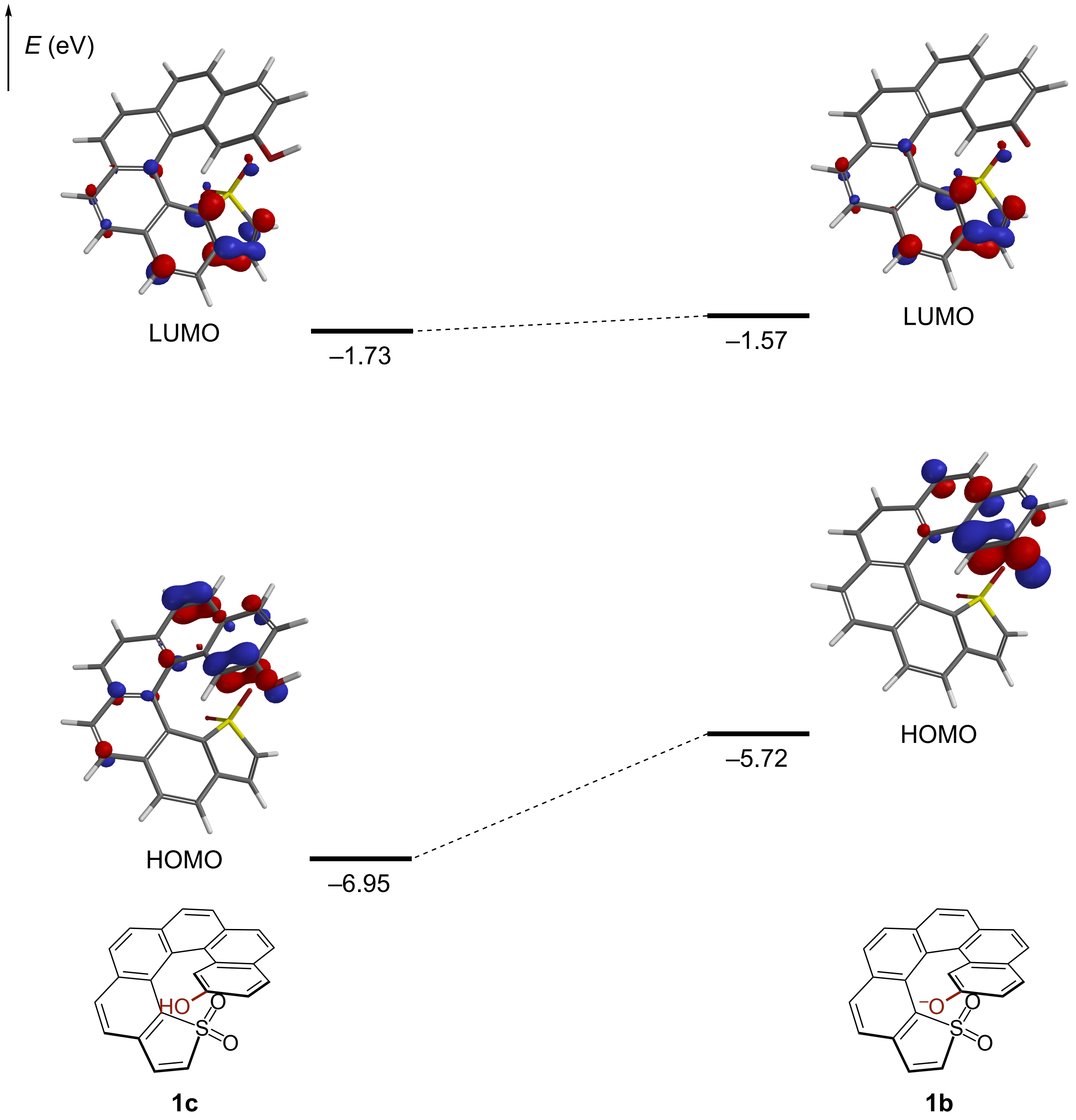

Figure S3. Frontier molecular orbitals and their energies for **1b** and **1c** (isovalue = 0.07). Upon deprotonation of **1c** to give **1b**, the HOMO energy of **1b** is significantly higher than that of **1c**, while the LUMO energy changes only slightly.

Optimized Cartesian coordinates (Å) and electronic energies (Hartree)

Opt/Freq: CPCM(MeOH); SP: SMD(MeOH)

1b

C	2.374382	-0.531027	-1.541211
C	2.838409	0.864453	0.821826
C	1.250367	-0.025428	-0.824694
C	3.667206	-0.49101	-0.963214
C	3.902897	0.163041	0.226136
C	1.553086	0.742383	0.322743
H	4.490209	-0.942009	-1.510689
H	4.901295	0.229417	0.646742
C	-0.078868	-0.208809	-1.375758
C	-0.156663	-0.374383	-2.776246
C	1.003437	-0.780865	-3.515557
C	2.200116	-0.967507	-2.895733
H	0.888649	-0.984273	-4.57688
H	3.061464	-1.351711	-3.434224
C	-1.402366	-0.166355	-3.436545
C	-2.510453	0.168401	-2.712285
C	-2.497632	0.106304	-1.285024
C	-1.296008	-0.209399	-0.603723
H	-1.440952	-0.244965	-4.519547
H	-3.449029	0.397839	-3.210191
C	-1.379152	-0.676694	0.779254
C	-2.617786	-0.489287	1.463229
C	-3.752737	0.033671	0.782818
C	-3.71782	0.280918	-0.559964
H	-4.674136	0.171237	1.345089
H	-4.607167	0.58938	-1.102103
C	-2.714113	-0.90657	2.817524
C	-1.673396	-1.528824	3.456255
C	-0.435013	-1.825323	2.771092
C	-0.345225	-1.375377	1.429423
C	1.928629	2.672787	2.040573
C	3.001132	1.904514	1.859631
H	1.754016	3.499371	2.717845
H	3.939912	2.029958	2.389424
S	0.580476	2.101747	1.030418
O	0.281849	3.025434	-0.077203
H	-3.654653	-0.739943	3.340296
H	-1.769487	-1.852072	4.490502
H	0.574623	-1.613186	0.90713
O	-0.531561	1.772811	1.924279
O	0.512063	-2.468645	3.358566

E(CPCM(MeOH)-M06-2X/6-31+G(d,p)) = −1545.891502

E(SMD(MeOH)-M06-2X/6-311+G(2d,p)) = −1546.238987

0 imaginary frequencies

TS11

C	-1.165122	-2.295155	-1.241314
C	-2.679376	-0.601865	0.416256
C	-0.502995	-1.287735	-0.519822
C	-2.600947	-2.36875	-1.239474
C	-3.330601	-1.50667	-0.46121
C	-1.275904	-0.430052	0.366972
H	-3.087583	-3.125465	-1.846847
H	-4.415977	-1.570922	-0.432476
C	0.889301	-1.045654	-0.751973
C	1.660952	-2.037436	-1.383979
C	0.994012	-3.147016	-1.986731
C	-0.372289	-3.228422	-1.981716
H	1.589958	-3.900659	-2.495115
H	-0.878197	-4.028019	-2.516533
C	3.089266	-1.894852	-1.419797
C	3.697224	-0.806711	-0.866267
C	2.921006	0.287871	-0.353135
C	1.514564	0.20652	-0.392634
H	3.677889	-2.688391	-1.872788
H	4.781169	-0.728453	-0.846784
C	0.731699	1.37125	-0.110047
C	1.34715	2.47888	0.4828
C	2.76185	2.505246	0.621014
C	3.534013	1.463934	0.166823
H	3.227949	3.379608	1.069265
H	4.618493	1.514247	0.21456
C	0.511065	3.550136	0.978603
C	-0.842078	3.519992	0.863412
C	-1.510442	2.485672	0.068669
C	-0.696479	1.376893	-0.37318
C	-2.445942	0.380419	2.555861
C	-3.296252	0.018278	1.573738
H	-2.636999	0.792519	3.537506
H	-4.374421	0.13067	1.651493
S	-0.829066	-0.217161	2.159399
O	0.211971	0.751025	2.53757
O	-0.626695	-1.58182	2.702747
H	0.998844	4.385296	1.477834
H	-1.456649	4.330588	1.2471
H	-1.017152	0.968739	-1.327381
O	-2.717208	2.593425	-0.272419

E(CPCM(MeOH)-M06-2X/6-31+G(d,p)) = −1545.866109

E(SMD(MeOH)-M06-2X/6-311+G(2d,p)) = −1546.209015

1 imaginary frequency −498.01 cm^{-1}

Intermediate **A**

C	-0.34716	-2.184593	-1.768513
C	-0.325001	-2.547546	1.047507
C	-0.094011	-1.064929	-0.956704
C	-0.71758	-3.452164	-1.19351
C	-0.698253	-3.593549	0.171349
C	-0.071625	-1.168315	0.541665
H	-0.961524	-4.281041	-1.849502
H	-0.886569	-4.569961	0.61763
C	0.076523	0.210487	-1.547218
C	0.238623	0.344239	-2.942455
C	0.082004	-0.813275	-3.749204
C	-0.247696	-2.01968	-3.182496
H	0.197807	-0.724373	-4.826649
H	-0.420152	-2.887916	-3.813761
C	0.55245	1.633118	-3.493104
C	0.663154	2.736153	-2.700211
C	0.381203	2.657075	-1.293015
C	0.039546	1.404011	-0.73068
H	0.714223	1.708147	-4.56568
H	0.923891	3.70195	-3.124213
C	-0.411463	1.330377	0.605091
C	-0.374961	2.459724	1.412127
C	0.040675	3.699493	0.867713
C	0.385518	3.8031	-0.461364
H	0.071424	4.573184	1.513679
H	0.66275	4.763625	-0.887057
C	-0.675317	2.331247	2.833624
C	-1.045593	1.166376	3.401844
C	-1.331603	-0.01826	2.581299
C	-0.951957	0.01298	1.109964
C	1.303226	-1.942259	2.683373
C	0.342227	-2.828443	2.264683
S	1.681304	-0.869029	1.347409
O	2.753242	-1.427817	0.477297
H	-0.565325	3.222561	3.448358
H	-1.256834	1.08839	4.464083
O	1.955269	0.511166	1.820835
O	-1.984953	-0.95475	3.033312
H	-1.913963	-0.150715	0.600079
H	2.046726	-2.089785	3.456009
H	0.169753	-3.769004	2.786405

E(CPCM(MeOH)-M06-2X/6-31+G(d,p)) = −1545.883541

E(SMD(MeOH)-M06-2X/6-311+G(2d,p)) = −1546.224434

0 imaginary frequencies

TS12

C	2.268196	-0.193674	-1.736751
C	2.531337	0.20371	1.084807
C	1.09892	-0.077767	-0.982184
C	3.572623	-0.214608	-1.077126
C	3.683412	-0.088106	0.265017
C	1.159835	0.203048	0.480826
H	4.451286	-0.368619	-1.696942
H	4.657303	-0.118787	0.748189
C	-0.163415	-0.296404	-1.583278
C	-0.263998	-0.425303	-2.985587
C	0.926344	-0.452388	-3.751503
C	2.157854	-0.379917	-3.138459
H	0.855483	-0.55623	-4.831131
H	3.067392	-0.458654	-3.728216
C	-1.56891	-0.507967	-3.589209
C	-2.697676	-0.457962	-2.831362
C	-2.626945	-0.394681	-1.393936
C	-1.35866	-0.384164	-0.769709
H	-1.632063	-0.588258	-4.671247
H	-3.678682	-0.486951	-3.298492
C	-1.258034	-0.452719	0.635726
C	-2.399063	-0.33633	1.418882
C	-3.666643	-0.291736	0.791851
C	-3.78333	-0.359999	-0.581448
H	-4.55594	-0.205339	1.411308
H	-4.762475	-0.364006	-1.053038
C	-2.262262	-0.138946	2.866667
C	-1.064084	-0.100578	3.471497
C	0.193083	-0.498089	2.774311
C	0.109992	-0.659677	1.250674
C	1.268513	1.360231	2.682678
C	2.548556	0.817552	2.310644
S	0.663623	1.941385	1.106237
O	1.484523	2.973561	0.424989
H	-3.173782	0.034064	3.435181
H	-0.974896	0.077811	4.5402
O	-0.776132	2.261591	1.142909
O	0.955619	-1.301169	3.393603
H	3.454685	0.930944	2.899943
H	1.156568	2.055308	3.511628
H	0.401844	-1.700178	1.051853

E(CPCM(MeOH)-M06-2X/6-31+G(d,p)) = −1545.869141

E(SMD(MeOH)-M06-2X/6-311+G(2d,p)) = −1546.212285

1 imaginary frequency −309.33 cm^{-1}

2b

C	2.256231	-0.142342	-1.77858
C	2.54581	0.127263	1.057157
C	1.093563	-0.086631	-1.006182
C	3.567761	-0.138363	-1.133744
C	3.702127	-0.080286	0.207375
C	1.172844	0.157493	0.455041
H	4.442272	-0.22626	-1.773224
H	4.681994	-0.110519	0.676677
C	-0.171197	-0.316618	-1.597849
C	-0.275825	-0.433454	-3.001931
C	0.906683	-0.412525	-3.780123
C	2.141661	-0.302864	-3.181201
H	0.826561	-0.505269	-4.860249
H	3.047533	-0.336595	-3.780974
C	-1.578623	-0.553411	-3.601286
C	-2.703931	-0.54168	-2.837254
C	-2.630221	-0.465879	-1.402249
C	-1.360889	-0.413935	-0.777016
H	-1.643226	-0.63229	-4.683484
H	-3.686045	-0.603592	-3.299664
C	-1.25864	-0.433848	0.629485
C	-2.406208	-0.322687	1.406118
C	-3.673512	-0.344773	0.778164
C	-3.787455	-0.450799	-0.592386
H	-4.56584	-0.272254	1.395647
H	-4.765608	-0.496066	-1.064691
C	-2.281482	-0.054111	2.842813
C	-1.089871	0.144319	3.423445
C	0.245992	-0.197224	2.79117
C	0.103298	-0.593406	1.272974
C	1.225995	1.058223	2.763556
C	2.565139	0.61031	2.32442
S	0.74285	1.93633	1.155469
O	1.698988	2.966326	0.708465
H	-3.204153	0.06355	3.408393
H	-1.026901	0.418215	4.475688
O	-0.672235	2.327962	1.094334
O	0.806181	-1.190057	3.528104
H	3.473356	0.795134	2.888406
H	1.168648	1.712358	3.634574
H	0.366378	-1.659746	1.234609

E(CPCM(MeOH)-M06-2X/6-31+G(d,p)) = −1545.883147

E(SMD(MeOH)-M06-2X/6-311+G(2d,p)) = −1546.229366

0 imaginary frequencies

TS13

C	-1.31788	-2.519237	0.581942
C	-2.729057	-0.127485	-0.059115
C	-0.58358	-1.377183	0.233063
C	-2.768987	-2.481941	0.5573
C	-3.439653	-1.355971	0.224413
C	-1.267746	-0.12114	-0.051081
H	-3.305367	-3.393357	0.806684
H	-4.525947	-1.331969	0.210165
C	0.83402	-1.430841	0.150218
C	1.510656	-2.612644	0.516151
C	0.751824	-3.752437	0.887668
C	-0.622469	-3.71267	0.903644
H	1.277135	-4.664184	1.159918
H	-1.194889	-4.596751	1.171047
C	2.948425	-2.623183	0.535033
C	3.659305	-1.500371	0.24251
C	3.000397	-0.281609	-0.141583
C	1.586811	-0.261517	-0.242981
H	3.454713	-3.543054	0.815055
H	4.745305	-1.506428	0.288438
C	0.922709	0.899192	-0.693749
C	1.639245	2.072908	-0.897612
C	3.047156	2.057277	-0.766227
C	3.718591	0.900051	-0.428796
H	3.599422	2.97816	-0.936913
H	4.803096	0.89042	-0.358377
C	0.917468	3.325986	-1.14522
C	-0.420771	3.385427	-1.127033
C	-1.343274	2.180159	-1.156221
C	-0.555857	0.828267	-1.005959
C	-2.366546	2.211222	0.037108
C	-3.310614	1.117389	-0.029192
S	-1.26996	1.440067	1.536034
O	-2.099661	1.072814	2.704173
H	1.514071	4.227361	-1.271149
H	-0.933057	4.339158	-1.245346
O	-0.072836	2.229959	1.87646
O	-2.036391	2.222686	-2.329555
H	-4.373424	1.246349	0.152353
H	-2.745628	3.199558	0.300619
H	-0.646761	0.369562	-2.003888

E(CPCM(MeOH)-M06-2X/6-31+G(d,p)) = −1545.881236

E(SMD(MeOH)-M06-2X/6-311+G(2d,p)) = −1546.225899

1 imaginary frequency −203.68 cm^{-1}

3b

C	-0.016108	-2.453458	-1.548519
C	0.230824	-2.404133	1.267451
C	-0.174888	-1.244465	-0.826321
C	0.266764	-3.641737	-0.829814
C	0.423734	-3.606125	0.537382
C	-0.121341	-1.246505	0.588963
H	0.385084	-4.574159	-1.376221
H	0.682893	-4.511778	1.080983
C	-0.2939	0	-1.542089
C	-0.327614	0	-2.944029
C	-0.296421	-1.249838	-3.646248
C	-0.126203	-2.42688	-2.976631
H	-0.359008	-1.236764	-4.731528
H	-0.052814	-3.365493	-3.520582
C	-0.296421	1.249838	-3.646248
C	-0.126203	2.42688	-2.976631
C	-0.016108	2.453458	-1.548519
C	-0.174888	1.244465	-0.826321
H	-0.359008	1.236764	-4.731528
H	-0.052814	3.365493	-3.520582
C	-0.121341	1.246505	0.588963
C	0.230824	2.404133	1.267451
C	0.423734	3.606125	0.537382
C	0.266764	3.641737	-0.829814
H	0.682893	4.511778	1.080983
H	0.385084	4.574159	-1.376221
C	0.422378	2.352207	2.719996
C	0.176459	1.238312	3.425798
C	-0.515567	0	2.882108
C	-0.568838	0	1.313388
C	0.176459	-1.238312	3.425798
C	0.422378	-2.352207	2.719996
H	0.793676	3.252873	3.206005
H	0.35244	1.224753	4.501407
O	-1.804156	0	3.390533
H	-1.664864	0	1.155847
H	0.35244	-1.224753	4.501407
H	0.793676	-3.252873	3.206005

E(CPCM(MeOH)-M06-2X/6-31+G(d,p)) = −997.397920

E(SMD(MeOH)-M06-2X/6-311+G(2d,p)) = −997.642299

0 imaginary frequencies

TS14

C	-0.025448	-2.46548	-1.551959
C	0.185178	-2.433964	1.275814
C	-0.113305	-1.244525	-0.837927
C	0.160964	-3.671257	-0.825486
C	0.292215	-3.644904	0.545517
C	-0.067057	-1.244001	0.583169
H	0.225236	-4.609883	-1.369495
H	0.477401	-4.567937	1.090747
C	-0.188972	0.000156	-1.552068
C	-0.230015	0.00022	-2.958324
C	-0.222548	-1.250018	-3.654514
C	-0.113137	-2.434032	-2.980475
H	-0.275415	-1.238725	-4.740418
H	-0.079115	-3.375237	-3.523532
C	-0.219414	1.250411	-3.654564
C	-0.108164	2.434206	-2.980457
C	-0.022562	2.465538	-1.551812
C	-0.112757	1.244728	-0.83777
H	-0.270918	1.239225	-4.740536
H	-0.070871	3.375291	-3.523522
C	-0.068252	1.244094	0.583398
C	0.18286	2.434177	1.27625
C	0.292498	3.644924	0.546002
C	0.164312	3.671178	-0.825269
H	0.477802	4.567845	1.09139
H	0.231661	4.609536	-1.369394
C	0.388505	2.380677	2.719772
C	0.221547	1.249289	3.433
C	-0.36714	-0.000642	2.83696
C	-0.342913	-0.00021	1.295292
C	0.226252	-1.248636	3.432248
C	0.393941	-2.37985	2.71887
H	0.723164	3.294432	3.208088
H	0.442778	1.233258	4.499768
O	-1.785241	-0.00332	3.030251
H	-1.585004	-0.002297	1.605923
H	0.450252	-1.231524	4.49842
H	0.731683	-3.292745	3.206646

E(CPCM(MeOH)-M06-2X/6-31+G(d,p)) = −997.374771

E(SMD(MeOH)-M06-2X/6-311+G(2d,p)) = −997.614539

1 imaginary frequency −1566.02 cm^{-1}

Intermediate **B**

C	-0.00817	-2.468112	-1.562255
C	0.174126	-2.459179	1.274005
C	-0.022943	-1.236477	-0.853761
C	0.099975	-3.684008	-0.834883
C	0.211881	-3.66366	0.541683
C	0.019316	-1.22651	0.578342
H	0.117244	-4.624418	-1.379047
H	0.335782	-4.598014	1.086613
C	-0.075094	0	-1.568741
C	-0.134851	0	-2.985607
C	-0.151976	-1.248322	-3.671568
C	-0.086962	-2.434695	-2.989333
H	-0.204306	-1.243274	-4.758072
H	-0.08673	-3.378377	-3.529618
C	-0.151976	1.248322	-3.671568
C	-0.086962	2.434695	-2.989333
C	-0.00817	2.468112	-1.562255
C	-0.022943	1.236477	-0.853761
H	-0.204306	1.243274	-4.758072
H	-0.08673	3.378377	-3.529618
C	0.019316	1.22651	0.578342
C	0.174126	2.459179	1.274005
C	0.211881	3.66366	0.541683
C	0.099975	3.684008	-0.834883
H	0.335782	4.598014	1.086613
H	0.117244	4.624418	-1.379047
C	0.388891	2.4022	2.700038
C	0.26523	1.248473	3.3977
C	-0.267751	0	2.759524
C	-0.03235	0	1.280181
C	0.26523	-1.248473	3.3977
C	0.388891	-2.4022	2.700038
H	0.69312	3.317639	3.205407
H	0.471298	1.216454	4.465949
H	0.471298	-1.216454	4.465949
H	0.69312	-3.317639	3.205407
O	-1.745295	0	3.019683
H	-2.176212	0	2.152559

E(CPCM(MeOH)-M06-2X/6-31+G(d,p)) = −997.441775
E(SMD(MeOH)-M06-2X/6-311+G(2d,p)) = −997.679612
0 imaginary frequencies

TS15

C	2.483598	0.014876	-1.57543
C	2.480291	0.199124	1.259067
C	1.256211	0.02155	-0.866232
C	3.703668	0.106839	-0.847566
C	3.692798	0.210885	0.524081
C	1.250731	0.083905	0.562454
H	4.64234	0.106041	-1.395152
H	4.629767	0.303124	1.069369
C	0.01857	-0.036173	-1.579365
C	0.017811	-0.111287	-2.992599
C	1.265719	-0.138128	-3.681768
C	2.45228	-0.074181	-3.000612
H	1.258575	-0.201531	-4.767238
H	3.395992	-0.086284	-3.540249
C	-1.230617	-0.14452	-3.680094
C	-2.416644	-0.086585	-2.997153
C	-2.446342	0.002578	-1.57219
C	-1.21819	0.015521	-0.864655
H	-1.224712	-0.207975	-4.765562
H	-3.361055	-0.103626	-3.53544
C	-1.211017	0.079368	0.564367
C	-2.440809	0.190756	1.262535
C	-3.654398	0.195184	0.528478
C	-3.666251	0.089257	-0.842678
H	-4.591113	0.28373	1.07482
H	-4.605518	0.083291	-1.389221
C	-2.39891	0.358978	2.687523
C	-1.22792	0.286014	3.377518
C	0.022537	-0.061764	2.712012
C	0.020435	0.06898	1.271698
C	1.272277	0.288962	3.377623
C	2.439692	0.366596	2.685067
H	-3.331118	0.566312	3.20895
H	-1.206445	0.429387	4.455221
H	1.251829	0.435709	4.454901
H	3.371946	0.582424	3.20328
O	0.0693	-1.915711	3.007201
H	-0.765309	-2.201628	2.607042

E(CPCM(MeOH)-M06-2X/6-31+G(d,p)) = −997.435655
E(SMD(MeOH)-M06-2X/6-311+G(2d,p)) = −997.677453
2 imaginary frequencies −434.60 cm^{-1}, −32.90 cm^{-1}*

**The small residual imaginary mode (ν = −32.90 cm^{-1}) corresponds to a shallow, collective out-of-plane bowl-flip motion of the polyaromatic framework. Visualization of the normal-mode displacement shows neither concerted elongation of the breaking C–O bond (HO^- departure) nor shortening of the nascent C=C bond beyond thermal noise.*

Coronene

C	2.459855	0	1.420198
C	2.459855	0	-1.420198
C	1.236302	0	0.713779
C	3.682034	0	0.684641
C	3.682034	0	-0.684641
C	1.236302	0	-0.713779
H	4.619852	0	1.233767
H	4.619852	0	-1.233767
C	0	0	1.427559
C	0	0	2.840396
C	1.248101	0	3.531056
C	2.433933	0	2.846415
H	1.241453	0	4.617793
H	3.378399	0	3.384026
C	-1.248101	0	3.531056
C	-2.433933	0	2.846415
C	-2.459855	0	1.420198
C	-1.236302	0	0.713779
H	-1.241453	0	4.617793
H	-3.378399	0	3.384026
C	-1.236302	0	-0.713779
C	-2.459855	0	-1.420198
C	-3.682034	0	-0.684641
C	-3.682034	0	0.684641
H	-4.619852	0	-1.233767
H	-4.619852	0	1.233767
C	-2.433933	0	-2.846415
C	-1.248101	0	-3.531056
C	0	0	-2.840396
C	0	0	-1.427559
C	1.248101	0	-3.531056
C	2.433933	0	-2.846415
H	-3.378399	0	-3.384026
H	-1.241453	0	-4.617793
H	1.241453	0	-4.617793
H	3.378399	0	-3.384026

E(CPCM(MeOH)-M06-2X/6-31+G(d,p)) = −921.576062
E(SMD(MeOH)-M06-2X/6-311+G(2d,p)) = −921.786926
0 imaginary frequencies

SO_2

S	0	0	-0.507193
O	-1.234366	0	0.253597
O	1.234366	0	0.253596

E(CPCM(MeOH)-M06-2X/6-31+G(d,p)) = −548.505666
E(SMD(MeOH)-M06-2X/6-311+G(2d,p)) = −548.602254
0 imaginary frequencies

MeOH

H	-1.155713	0	0.708296
C	-0.127293	0	0.344234
H	0.38098	0.892495	0.724811
H	0.38098	-0.892495	0.724811
O	-0.189533	0	-1.07758
H	0.71058	0	-1.424573

E(CPCM(MeOH)-M06-2X/6-31+G(d,p)) = −115.680172
E(SMD(MeOH)-M06-2X/6-311+G(2d,p)) = −115.718180
0 imaginary frequencies

$HO^{-}\cdots$MeOH cluster

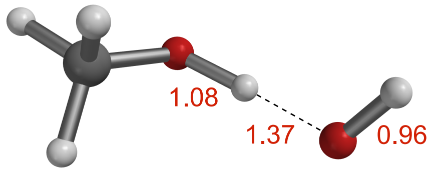

C	-0.011406	-0.106644	1.098448
O	-0.961402	-0.100487	0.065567
H	-0.436061	-0.089736	-0.88238
H	0.414041	0.752675	-2.407599
O	0.22978	-0.134473	-2.078814
H	-0.515258	-0.033424	2.069915
H	0.591611	-1.029073	1.108929
H	0.688695	0.741162	1.025936

∠O–H···O = 177.56°, ∠H···O–H = 111.15°
E(CPCM(MeOH)-M06-2X/6-31+G(d,p)) = −191.598450
E(SMD(MeOH)-M06-2X/6-311+G(2d,p)) = −191.668833
0 imaginary frequencies

Opt/Freq: CPCM(solvent); SP: SMD(solvent)

1c (MeOH)

C	2.368332	-0.460265	-1.643656
C	2.847925	0.943319	0.70843
C	1.24643	0.039501	-0.918313
C	3.667676	-0.406977	-1.082959
C	3.912581	0.255102	0.099078
C	1.555748	0.805574	0.230316
H	4.487034	-0.852576	-1.639929
H	4.915788	0.335586	0.505431
C	-0.082673	-0.146808	-1.471146
C	-0.16624	-0.320623	-2.869588
C	0.992388	-0.724853	-3.613198
C	2.190716	-0.901609	-2.995999
H	0.87457	-0.931084	-4.673373
H	3.053233	-1.280613	-3.53618
C	-1.412784	-0.118742	-3.530338
C	-2.523114	0.220115	-2.811626
C	-2.505263	0.164545	-1.385932
C	-1.305345	-0.143816	-0.706174
H	-1.448893	-0.203895	-4.612744
H	-3.461315	0.447327	-3.310358
C	-1.401244	-0.599411	0.673887
C	-2.63054	-0.421875	1.364255
C	-3.770485	0.111833	0.683436
C	-3.72519	0.346335	-0.655312
H	-4.689096	0.259921	1.245236
H	-4.6103	0.661538	-1.200701
C	-2.734066	-0.834518	2.712977
C	-1.688997	-1.458706	3.353437
C	-0.504377	-1.720101	2.632642
C	-0.36387	-1.306077	1.324044
C	1.93495	2.732847	1.952805
C	3.016314	1.984669	1.743758
H	1.761154	3.554462	2.636457
H	3.965951	2.127048	2.249241
S	0.570633	2.129705	0.98531
O	0.189448	3.054301	-0.093029
H	-3.670809	-0.668473	3.238519
H	-1.776748	-1.782279	4.387176
H	0.55384	-1.562881	0.809519
O	-0.48723	1.736073	1.918683
O	0.536192	-2.398344	3.196041
H	0.317676	-2.665276	4.099877

E(CPCM(MeOH)-M06-2X/6-31+G(d,p)) = −1546.370881
E(SMD(MeOH)-M06-2X/6-311+G(2d,p)) = −1546.715717
0 imaginary frequencies

1c (DMSO)

C	2.368318	-0.460281	-1.643642
C	2.847942	0.943325	0.708446
C	1.246431	0.039517	-0.918312
C	3.667663	-0.407014	-1.082922
C	3.912585	0.255076	0.099121
C	1.555758	0.805591	0.230312
H	4.487018	-0.852626	-1.639887
H	4.915791	0.335553	0.505477
C	-0.082675	-0.146781	-1.471142
C	-0.166258	-0.320608	-2.869586
C	0.992356	-0.724863	-3.613197
C	2.19068	-0.901641	-2.995988
H	0.874533	-0.931099	-4.673371
H	3.05319	-1.280678	-3.536157
C	-1.412803	-0.118699	-3.530342
C	-2.523127	0.220182	-2.811632
C	-2.505267	0.16459	-1.385937
C	-1.305338	-0.143783	-0.706172
H	-1.448915	-0.203857	-4.612749
H	-3.461326	0.447407	-3.310359
C	-1.401225	-0.59941	0.673886
C	-2.630533	-0.421922	1.364245
C	-3.770488	0.111786	0.683428
C	-3.725194	0.346354	-0.655315
H	-4.689113	0.259815	1.245226
H	-4.610315	0.661545	-1.200696
C	-2.734079	-0.83461	2.712965
C	-1.689011	-1.458782	3.353454
C	-0.504368	-1.720126	2.632677
C	-0.363844	-1.306063	1.324072
C	1.934991	2.732926	1.952756
C	3.016339	1.984711	1.743754
H	1.761202	3.554569	2.636383
H	3.965969	2.127079	2.249258
S	0.57072	2.129769	0.985261
O	0.189503	3.054334	-0.093148
H	-3.670851	-0.668627	3.23848
H	-1.776778	-1.782392	4.387178
H	0.5539	-1.562815	0.809576
O	-0.487252	1.736228	1.918593
O	0.536206	-2.398327	3.196096
H	0.317667	-2.665352	4.099912

E(CPCM(DMSO)-M06-2X/6-31+G(d,p)) = −1546.371306
E(SMD(DMSO)-M06-2X/6-311+G(2d,p)) = −1546.704949
0 imaginary frequencies

TS10 (MeOH)

C	-1.063038	-2.392294	-1.206662
C	-2.606447	-0.582514	0.322939
C	-0.405429	-1.346649	-0.551238
C	-2.511792	-2.458723	-1.214743
C	-3.243854	-1.550818	-0.510847
C	-1.168334	-0.386173	0.275048
H	-2.991783	-3.245999	-1.787432
H	-4.329779	-1.608515	-0.491708
C	0.983882	-1.13358	-0.77155
C	1.757845	-2.129811	-1.392327
C	1.094047	-3.260071	-1.942597
C	-0.275153	-3.354168	-1.903564
H	1.684454	-4.029323	-2.43329
H	-0.781188	-4.181425	-2.393758
C	3.187188	-1.965017	-1.456922
C	3.792449	-0.85869	-0.94427
C	3.012081	0.228486	-0.412262
C	1.608534	0.114869	-0.401725
H	3.777025	-2.75808	-1.908736
H	4.874267	-0.759859	-0.962236
C	0.817907	1.238603	-0.055733
C	1.412965	2.368035	0.498813
C	2.831883	2.443637	0.566153
C	3.609382	1.420919	0.080949
H	3.290519	3.329132	0.997672
H	4.692952	1.500107	0.089421
C	0.569559	3.393698	1.066363
C	-0.793504	3.320966	1.023859
C	-1.411031	2.273819	0.286061
C	-0.653661	1.167986	-0.26069
C	-2.351573	0.535193	2.383266
C	-3.21616	0.05685	1.433466
H	-2.573285	0.960096	3.353413
H	-4.296743	0.127915	1.528589
S	-0.800471	-0.264606	2.132102
O	0.326849	0.586018	2.546703
O	-0.779678	-1.638901	2.675808
H	1.046047	4.240707	1.55292
H	-1.41533	4.104567	1.447433
H	-0.910721	1.030601	-1.315511
O	-2.654155	2.360702	-0.173
H	-3.136728	3.102309	0.229825

E(CPCM(MeOH)-M06-2X/6-31+G(d,p)) = −1546.318980

E(SMD(MeOH)-M06-2X/6-311+G(2d,p)) = −1546.661273

1 imaginary frequency −191.90 cm^{-1}

TS10 (DMSO)

C	-1.063058	-2.392293	-1.206689
C	-2.606472	-0.582542	0.322952
C	-0.405454	-1.346616	-0.551303
C	-2.511805	-2.458756	-1.214735
C	-3.243874	-1.550857	-0.510813
C	-1.168377	-0.386166	0.275005
H	-2.991778	-3.246042	-1.787423
H	-4.329797	-1.608589	-0.491649
C	0.983855	-1.133533	-0.771608
C	1.757835	-2.129795	-1.392332
C	1.09405	-3.260072	-1.942573
C	-0.275158	-3.354179	-1.903555
H	1.684483	-4.029336	-2.433216
H	-0.781175	-4.181465	-2.393724
C	3.187178	-1.965029	-1.456857
C	3.792428	-0.858684	-0.944206
C	3.01205	0.228509	-0.412271
C	1.608496	0.114919	-0.401802
H	3.777042	-2.758123	-1.90858
H	4.874248	-0.759881	-0.962111
C	0.817874	1.238662	-0.055806
C	1.412946	2.368065	0.498807
C	2.831861	2.443652	0.56617
C	3.609363	1.420922	0.080966
H	3.290508	3.329148	0.997677
H	4.692932	1.500111	0.089445
C	0.56956	3.393714	1.066386
C	-0.793513	3.321032	1.023834
C	-1.411032	2.273954	0.285945
C	-0.653688	1.168101	-0.260771
C	-2.351505	0.535123	2.383311
C	-3.216131	0.056817	1.433527
H	-2.573159	0.960046	3.353467
H	-4.296712	0.127941	1.528676
S	-0.800442	-0.264667	2.131991
O	0.327029	0.585769	2.546609
O	-0.779644	-1.639131	2.675658
H	1.046055	4.240676	1.553017
H	-1.41532	4.104641	1.447413
H	-0.910812	1.030639	-1.315567
O	-2.654162	2.360855	-0.173051
H	-3.136725	3.102458	0.229783

E(CPCM(DMSO)-M06-2X/6-31+G(d,p)) = −1546.319455

E(SMD(DMSO)-M06-2X/6-311+G(2d,p)) = −1546.649035

1 imaginary frequency −191.60 cm^{-1}

2c (MeOH)

C	2.21466	-0.163061	-1.904207
C	2.556157	0.196435	0.915675
C	1.065949	-0.083815	-1.114329
C	3.53643	-0.134301	-1.283299
C	3.697817	-0.034222	0.052245
C	1.176254	0.209538	0.333193
H	4.399326	-0.237958	-1.935334
H	4.685493	-0.045724	0.504021
C	-0.209598	-0.33069	-1.673106
C	-0.340654	-0.494191	-3.069049
C	0.827673	-0.499987	-3.869
C	2.074142	-0.369848	-3.298
H	0.727328	-0.628904	-4.943317
H	2.968026	-0.423114	-3.913222
C	-1.655637	-0.631609	-3.638143
C	-2.766958	-0.591122	-2.855217
C	-2.66551	-0.466894	-1.425161
C	-1.38361	-0.395804	-0.829506
H	-1.740927	-0.747157	-4.715217
H	-3.757195	-0.665805	-3.296485
C	-1.259893	-0.366972	0.573645
C	-2.390118	-0.236141	1.367443
C	-3.670203	-0.27167	0.769271
C	-3.807661	-0.421206	-0.595078
H	-4.549075	-0.176778	1.401566
H	-4.793664	-0.477893	-1.047899
C	-2.232436	0.075699	2.792572
C	-1.036262	0.284254	3.354944
C	0.268055	-0.01487	2.664154
C	0.115041	-0.505188	1.192895
C	1.264603	1.179026	2.61947
C	2.595687	0.711169	2.170888
S	0.750812	2.020029	0.988531
O	1.716434	3.031027	0.541342
H	-3.139273	0.223524	3.374295
H	-0.948625	0.594242	4.393551
O	-0.661981	2.408254	0.946964
O	0.947096	-1.002838	3.44069
H	0.320895	-1.71836	3.628886
H	1.221306	1.852207	3.475586
H	3.507987	0.924122	2.716072
H	0.372109	-1.573398	1.187673

E(CPCM(MeOH)-M06-2X/6-31+G(d,p)) = −1546.376838

E(SMD(MeOH)-M06-2X/6-311+G(2d,p)) = −1546.718048

0 imaginary frequencies

2c (DMSO)

C	2.214673	-0.163061	-1.904171
C	2.556159	0.196465	0.915731
C	1.065937	-0.083793	-1.114319
C	3.536463	-0.13436	-1.28324
C	3.697844	-0.03427	0.052318
C	1.176227	0.209451	0.33322
H	4.399348	-0.238142	-1.935277
H	4.685502	-0.04587	0.504141
C	-0.20962	-0.330625	-1.673092
C	-0.340664	-0.494119	-3.069045
C	0.827687	-0.499965	-3.868989
C	2.074177	-0.369863	-3.297975
H	0.727343	-0.628938	-4.943302
H	2.968075	-0.423233	-3.91317
C	-1.655654	-0.631533	-3.638168
C	-2.76699	-0.591045	-2.855234
C	-2.665538	-0.466821	-1.425156
C	-1.383634	-0.395733	-0.82948
H	-1.740913	-0.747127	-4.715242
H	-3.757235	-0.66577	-3.296486
C	-1.259918	-0.366937	0.573684
C	-2.390149	-0.23615	1.367497
C	-3.670247	-0.271695	0.769314
C	-3.807706	-0.421186	-0.595063
H	-4.549118	-0.176937	1.401637
H	-4.793706	-0.47795	-1.04789
C	-2.232489	0.075627	2.792664
C	-1.036306	0.284244	3.355012
C	0.268018	-0.014842	2.664192
C	0.115004	-0.505209	1.192947
C	1.264528	1.179156	2.619504
C	2.595668	0.711319	2.170899
S	0.750895	2.01989	0.988509
O	1.716516	3.030995	0.541021
H	-3.139339	0.223244	3.374422
H	-0.94864	0.594111	4.393652
O	-0.661815	2.40884	0.946584
O	0.947165	-1.00269	3.440718
H	0.321324	-1.718756	3.628226
H	1.221198	1.852443	3.475543
H	3.507945	0.924277	2.716139
H	0.371987	-1.573439	1.187723

E(CPCM(DMSO)-M06-2X/6-31+G(d,p)) = −1546.377192

E(SMD(DMSO)-M06-2X/6-311+G(2d,p)) = −1546.707866

0 imaginary frequencies

Table S1. Absolute energy contributions (kcal mol^{-1}) at 298.15 K for the transformation of **1b** into **coronene**: CPCM(MeOH) frequencies and SMD(MeOH) single-point energies

	$E_{\text{elec, CPCM}}$	$H_{\text{corr, CPCM}}$	$-T{\cdot}S_{\text{CPCM}}$	G_{CPCM}	$G_{\text{corr, CPCM}}$	$E_{\text{elec, SMD}}$	G_{final}
**1b**	-970061.6035	-969860.0913	-38.278707	-969898.3700	163.23341	-970279.6536	-970116.4202
**TS11**	-970045.6691	-969844.7167	-38.373460	-969883.0901	162.57892	-970260.8458	-970097.9709
Int **A**	-970056.6078	-969855.3599	-38.203405	-969893.5633	163.04453	-970270.5214	-970107.4769
**TS12**	-970047.5717	-969845.9554	-37.831920	-969883.7873	163.78437	-970262.8978	-970098.8174
**2b**	-970056.3606	-969853.8532	-37.392036	-969891.2453	165.11532	-970273.6163	-970108.5010
**TS13**	-970055.1614	-969853.3732	-37.804309	-969891.1775	163.98392	-970271.4407	-970107.1608
**3b**	-625876.6700	-625682.2048	-34.451526	-625716.6564	160.01366	-626030.0202	-625870.0065
**TS14**	-625862.1438	-625670.4892	-34.777204	-625705.2664	156.87737	-626012.6005	-625855.4271
Int **B**	-625904.1895	-625710.4515	-34.988047	-625745.4396	158.74986	-626053.4344	-625894.6846
**TS15**	-625900.3491	-625707.1421	-35.613674	-625742.7557	157.59336	-626052.0796	-625894.1433
**Coronene**	-578297.7338	-578112.3926	-31.920780	-578144.3134	153.42042	-578430.0530	-578276.6326
**SO_2**	-344192.5162	-344185.5671	-17.708318	-344203.2754	-10.759278	-344253.1261	-344263.8853
**MeOH**	-72590.40689	-72555.25569	-16.990447	-72572.24613	18.160752	-72614.25727	-72596.09652
**HO^-···MeOH**	-120229.8475	-120187.8734	-20.555956	-120208.4294	21.418154	-120274.0135	-120252.5954
**HO^-**							-47656.49889

	1 M standard-state correction	ΔG (rel. **1b**)
**1b**	0	0.00
**TS11**	0	18.45
Int **A**	0	8.94
**TS12**	0	17.60
**2b**	0	7.92
**TS13**	0	9.26
**3b** + **SO_2**	1.89	-15.58
**TS14** + **SO_2**	1.89	-1.00
Int **B** + **SO_2**	1.89	-40.26
**TS15** + **SO_2**	1.89	-39.72
**Coronene** + **SO_2** + **HO^-**	3.78	-76.82

- TS thermochemistry follows the conventions stated in **Thermochemistry conventions** (main imaginary excluded; low-frequency clamping applied; **TS15** residual −32.90 cm^{-1} inverted).
- Energies are reported per species. Reaction ΔG values are reported relative to **1b** and include the 1 M standard-state correction where stated elsewhere.

Table S2. Absolute energy contributions (kcal mol^{-1}) at 298.15 K for the intramolecular Diels–Alder reaction of **1c** to give **2c**: CPCM(MeOH) frequencies and SMD(MeOH) single-point energies

	$E_{\text{elec, CPCM}}$	$H_{\text{corr, CPCM}}$	$-T{\cdot}S_{\text{CPCM}}$	G_{CPCM}	$G_{\text{corr, CPCM}}$	$E_{\text{elec, SMD}}$	G_{final}
**1c**	-970362.4183	-970151.7069	-38.520298	-970190.2272	172.191117	-970578.8062	-970406.6151
**TS10**	-970329.8499	-970120.2128	-38.392914	-970158.6057	171.244205	-970544.6420	-970373.1018
**2c**	-970366.1564	-970154.4980	-37.520048	-970192.0181	174.138279	-970580.2689	-970406.1306

	1 M standard-state correction	ΔG (rel. **1c**)
**1c**	0	0.00
**TS10**	0	33.51
**2c**	0	0.48

- TS thermochemistry follows the conventions stated in **Thermochemistry conventions** (main imaginary excluded; low-frequency clamping applied).
- Energies are reported per species. Reaction ΔG values are reported relative to **1c** and include the 1 M standard-state correction where stated elsewhere.

Table S3. Absolute energy contributions (kcal mol^{-1}) at 433.15 K (160 °C) for the intramolecular Diels–Alder reaction of **1c** to give **2c**: CPCM(DMSO) frequencies and SMD(DMSO) single-point energies

	$E_{\text{elec, CPCM}}$	$H_{\text{corr, CPCM}}$	$-T{\cdot}S_{\text{CPCM}}$	G_{CPCM}	$G_{\text{corr, CPCM}}$	$E_{\text{elec, SMD}}$	G_{final}
**1c**	-970362.6850	-970139.7020	-68.944469	-970208.6465	154.038522	-970572.0491	-970418.0106
**TS10**	-970330.1480	-970108.2073	-68.787591	-970176.9949	153.153106	-970536.9626	-970383.3795
**2c**	-970366.3785	-970142.5070	-67.764751	-970210.2717	156.106793	-970573.8796	-970417.7728

	1 M standard-state correction	ΔG (rel. **1c**)
**1c**	0	0.00
**TS10**	0	34.63
**2c**	0	0.24

- TS thermochemistry follows the conventions stated in **Thermochemistry conventions** (main imaginary excluded; low-frequency clamping applied).
- Energies are reported per species. Reaction ΔG values are reported relative to **1c** and include the 1 M standard-state correction where stated elsewhere.

4. NMR Spectra

Compound **4**

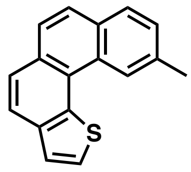

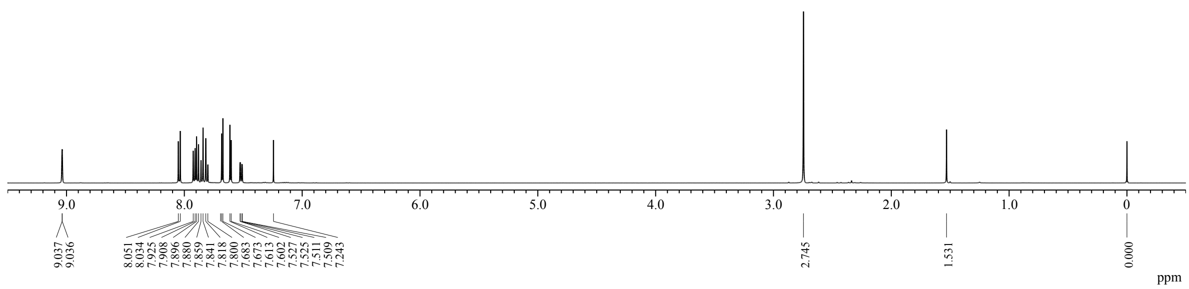

Figure S4. ¹H NMR spectrum (500 MHz, $CDCl_3$, 298 K) of **4**.

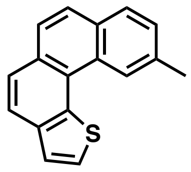

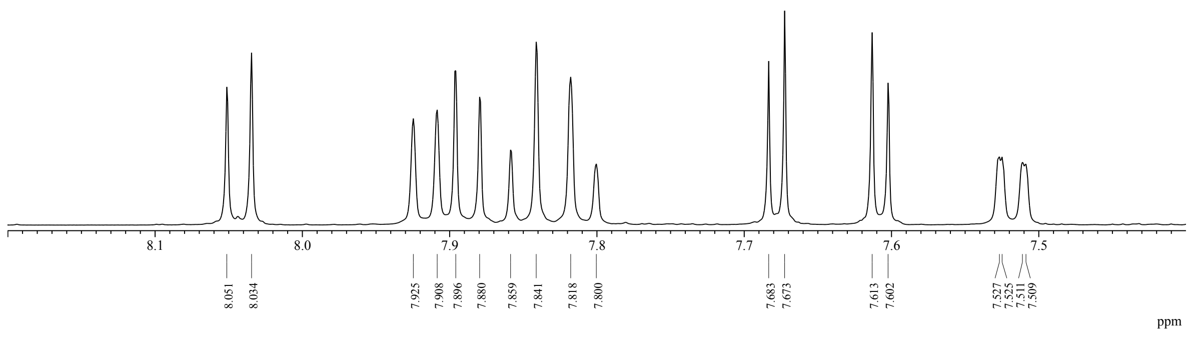

Figure S5. Enlarged ¹H NMR spectrum (500 MHz, $CDCl_3$, 298 K) of **4**.

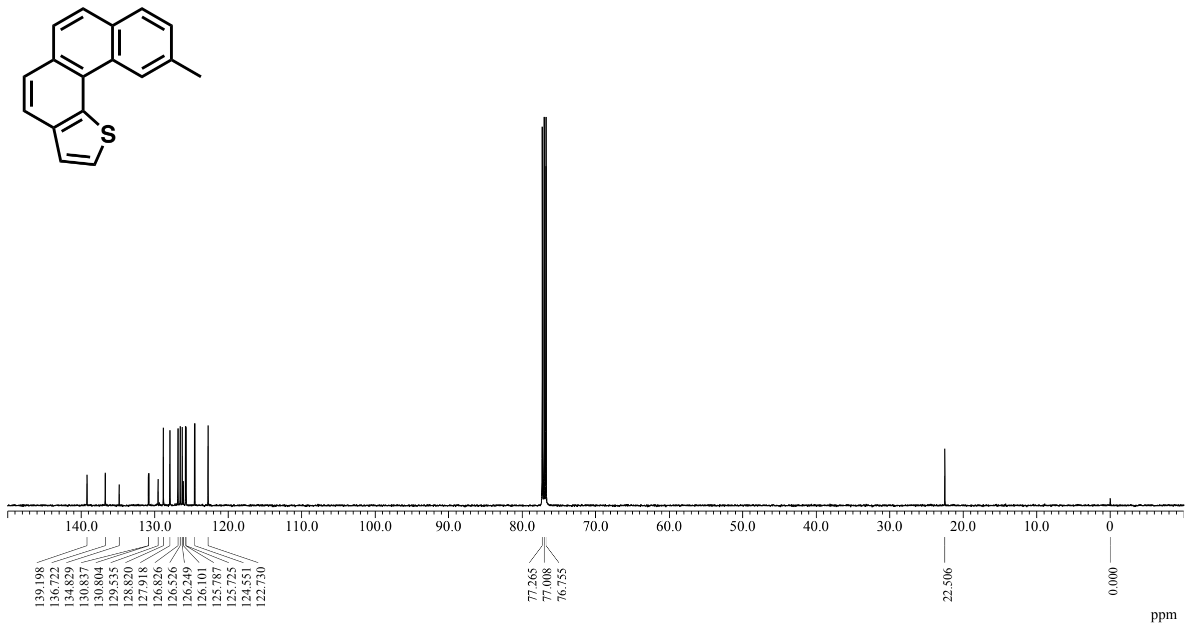

Figure S6. $^{13}C\{^{1}H\}$ NMR spectrum (125 MHz, $CDCl_3$, 298 K) of **4**.

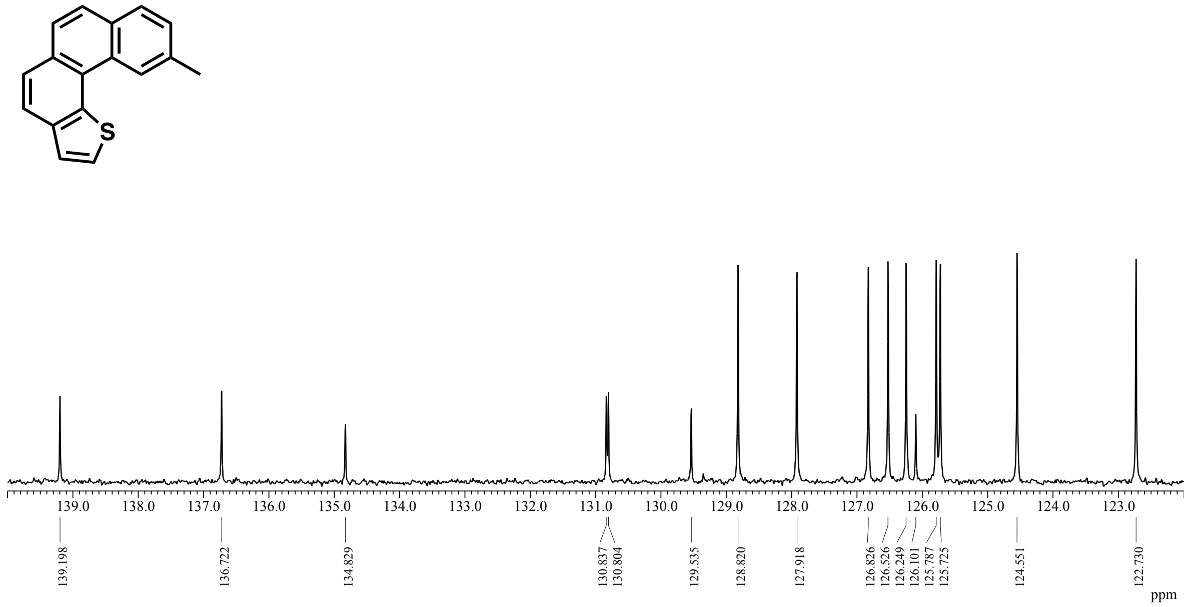

Figure S7. Enlarged $^{13}C\{^{1}H\}$ NMR spectrum (125 MHz, $CDCl_3$, 298 K) of **4**.

Compound **5**

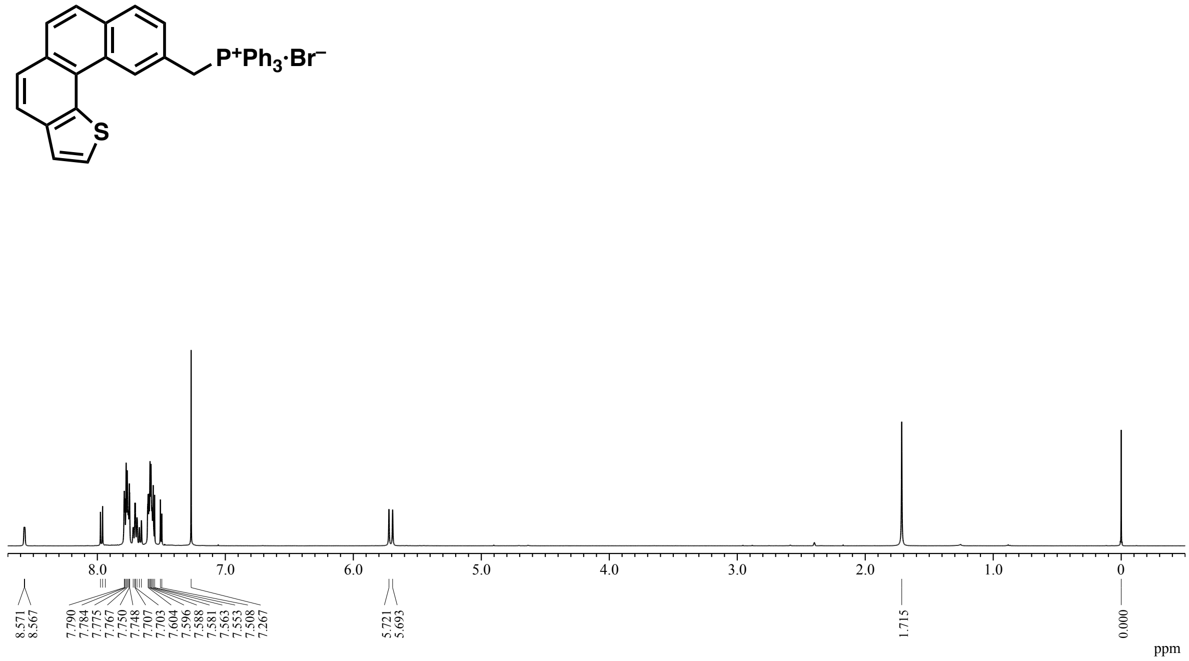

Figure S8. ^{1}H NMR spectrum (500 MHz, $CDCl_3$, 298 K) of **5**.

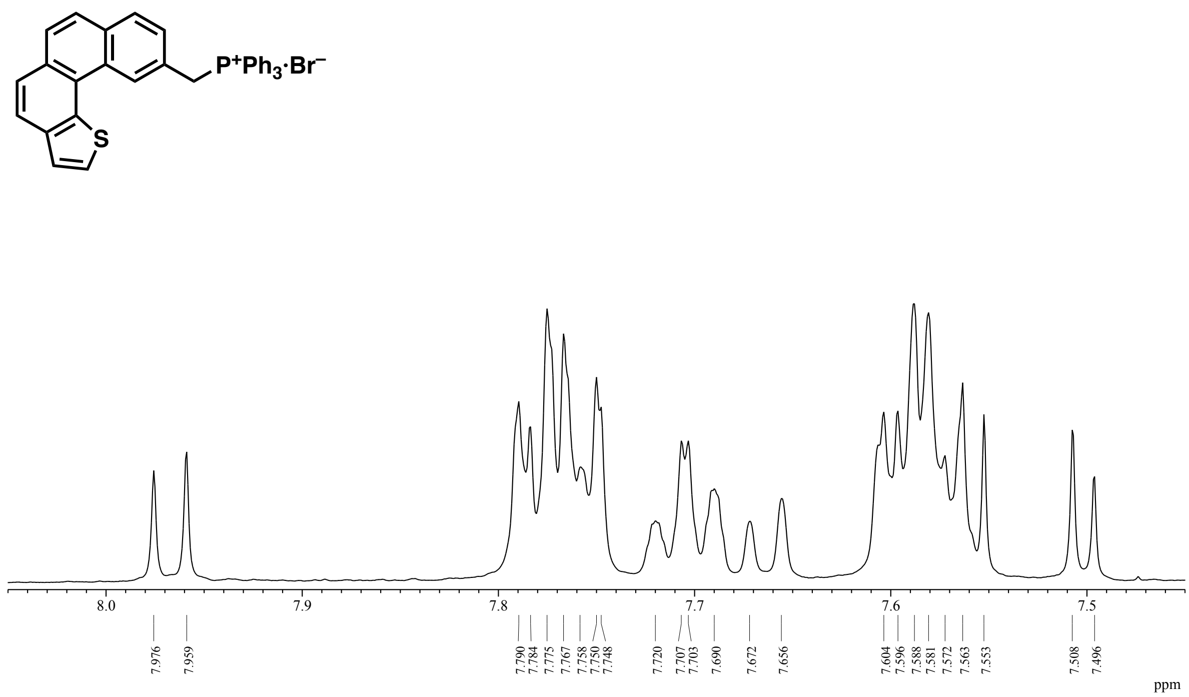

Figure S9. Enlarged ^{1}H NMR spectrum (500 MHz, $CDCl_3$, 298 K) of **5**.

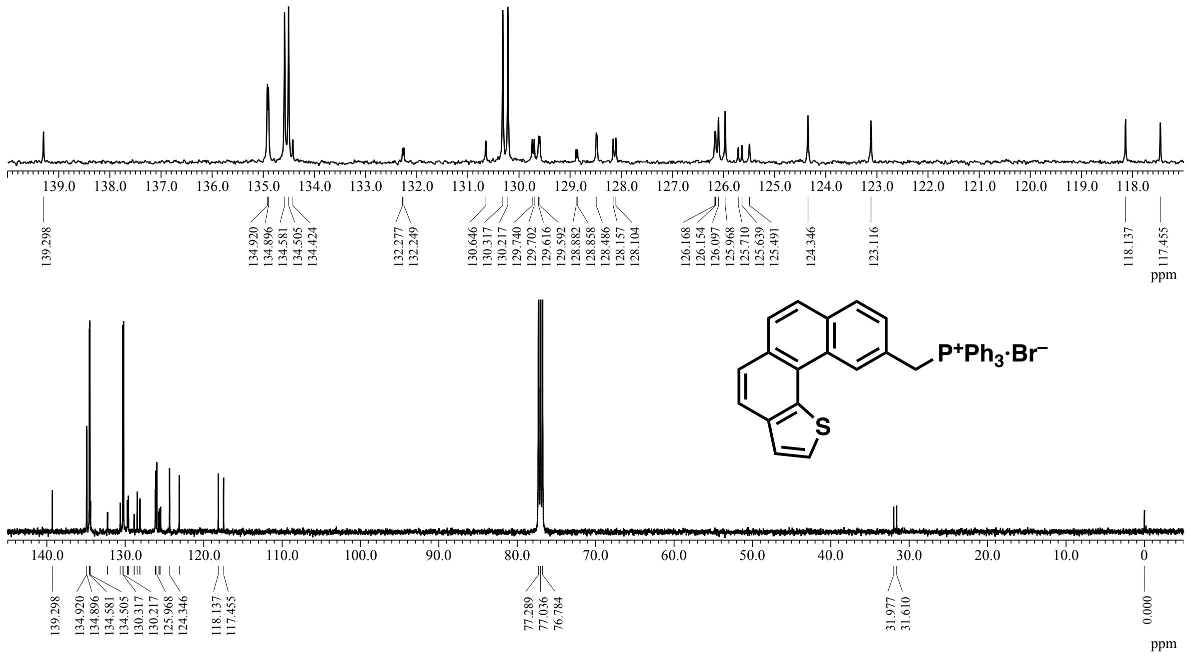

Figure S10. $^{13}C\{^{1}H\}$ NMR spectrum (125 MHz, $CDCl_3$, 298 K) of **5**.

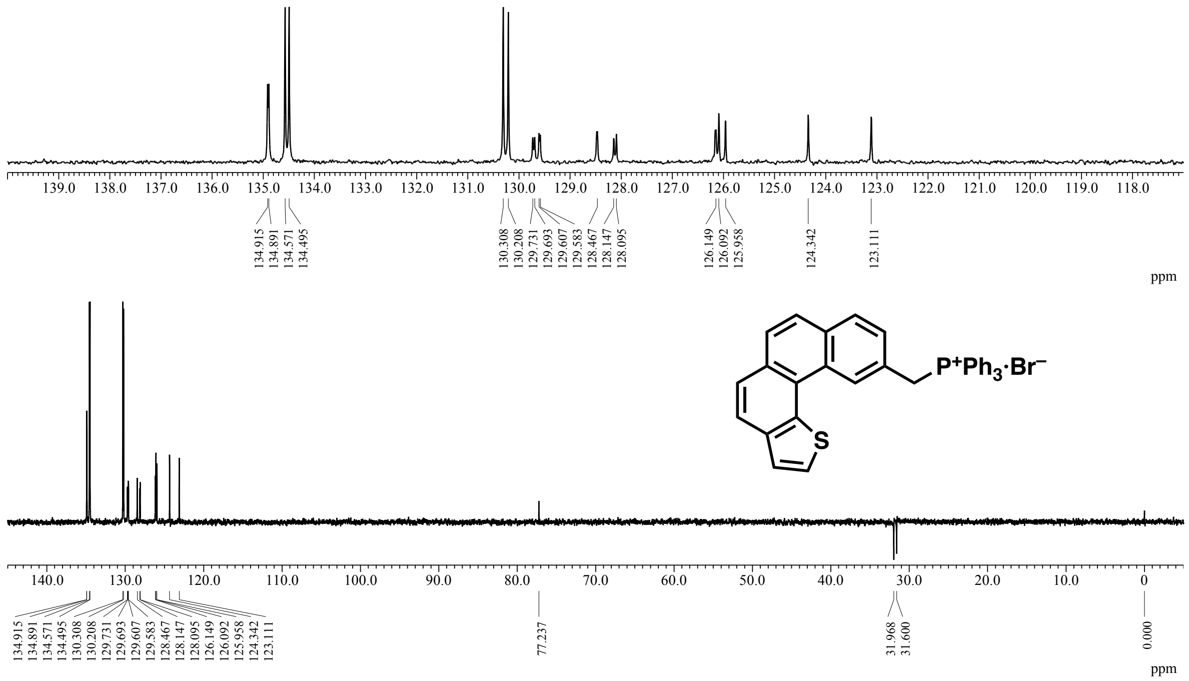

Figure S11. ^{13}C DEPT-135 NMR spectrum (125 MHz, $CDCl_3$, 298 K) of **5**.

Compound (*Z*)-**6**

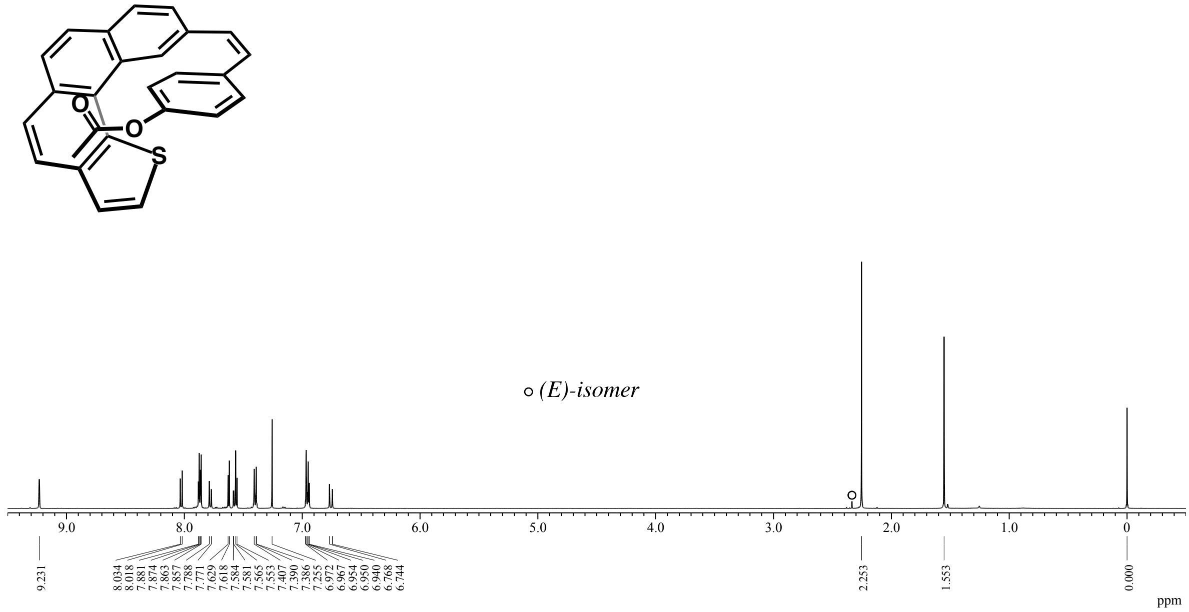

Figure S12. ^{1}H NMR spectrum (500 MHz, $CDCl_3$, 298 K) of (*Z*)-**6**.

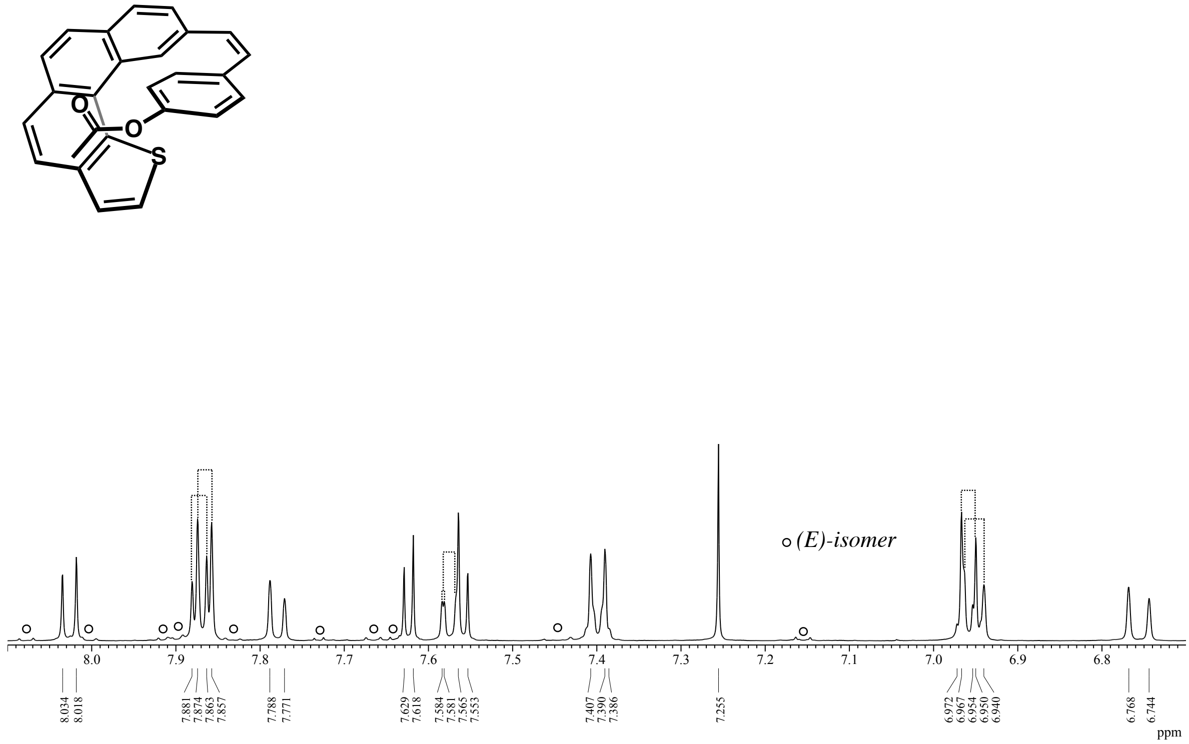

Figure S13. Enlarged ^{1}H NMR spectrum (500 MHz, $CDCl_3$, 298 K) of (*Z*)-**6**.

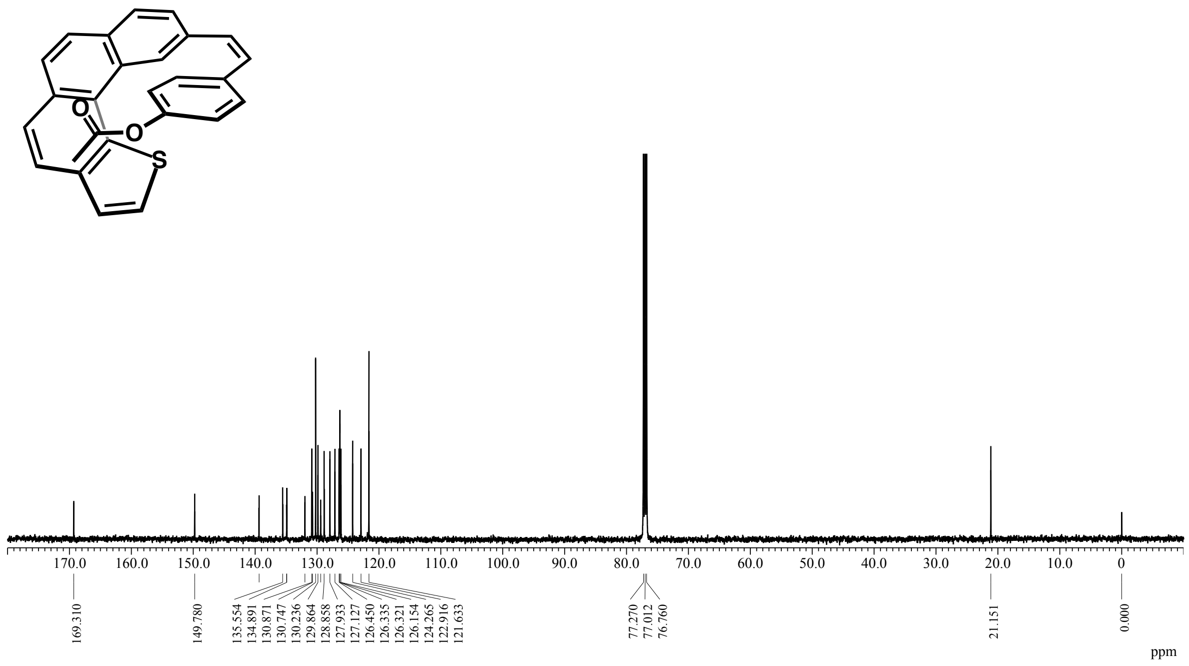

Figure S14. $^{13}C\{^{1}H\}$ NMR spectrum (125 MHz, $CDCl_3$, 298 K) of (*Z*)-**6**.

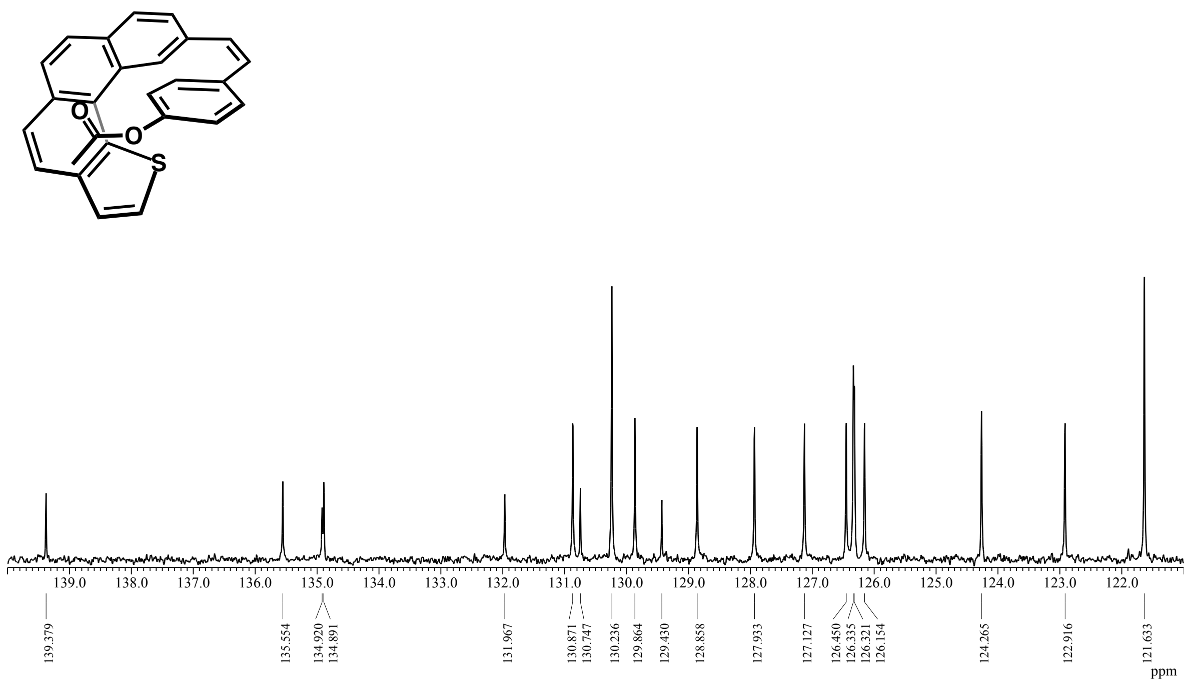

Figure S15. Enlarged $^{13}C\{^{1}H\}$ NMR spectrum (125 MHz, $CDCl_3$, 298 K) of (*Z*)-**6**.

Compound (*E*)-**6**

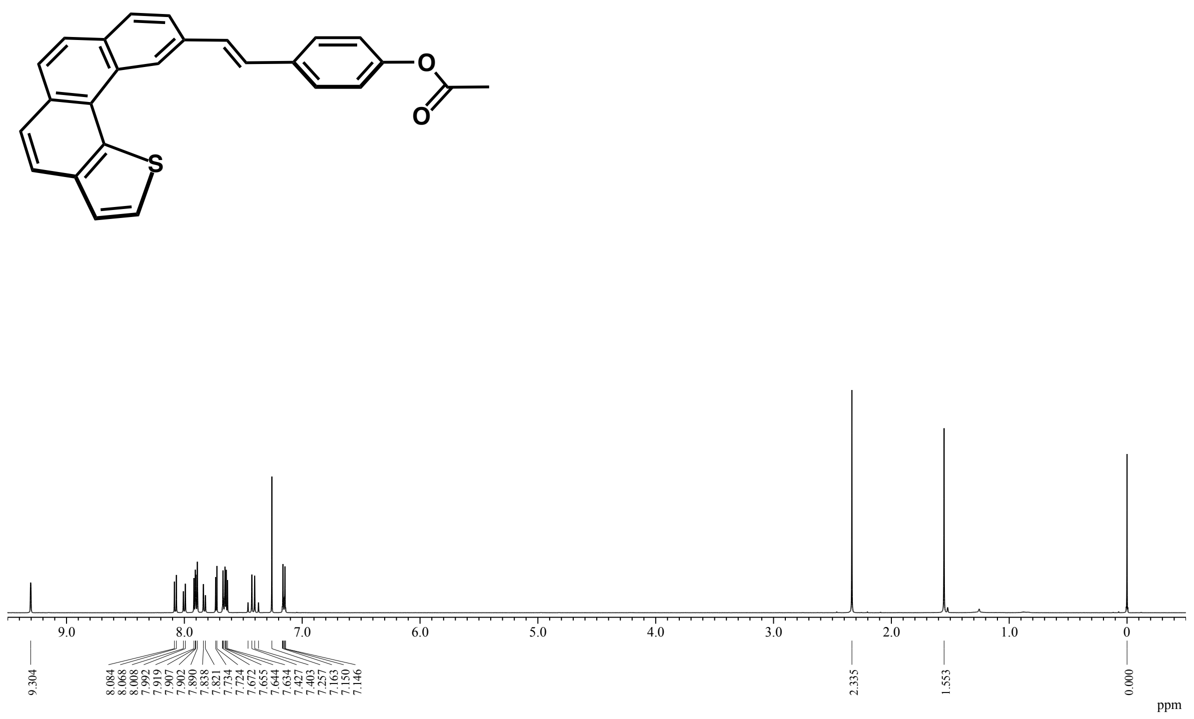

Figure S16. ^{1}H NMR spectrum (500 MHz, $CDCl_3$, 298 K) of (*E*)-**6**.

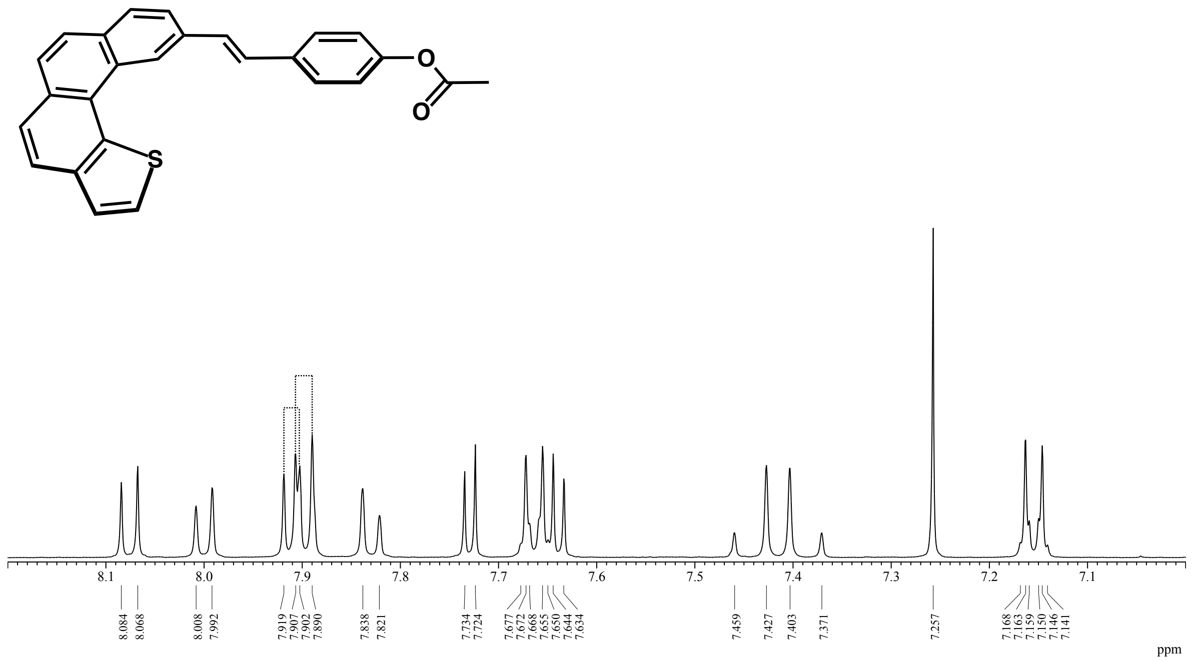

Figure S17. Enlarged ^{1}H NMR spectrum (500 MHz, $CDCl_3$, 298 K) of (*E*)-**6**.

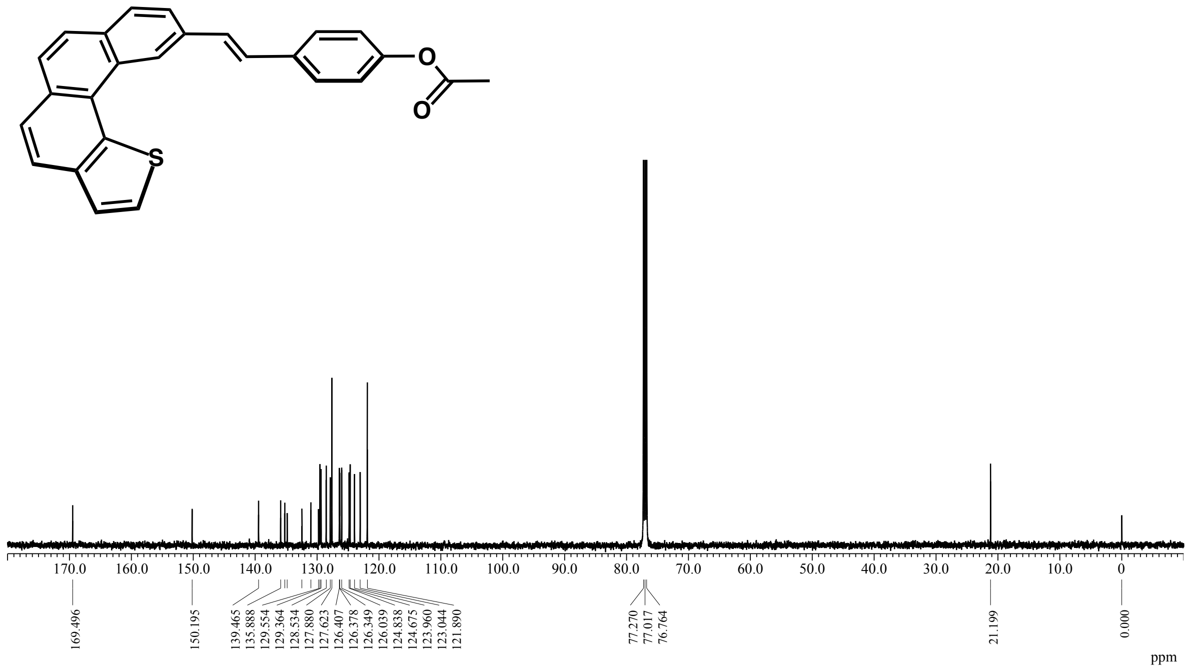

Figure S18. $^{13}C\{^{1}H\}$ NMR spectrum (125 MHz, $CDCl_3$, 298 K) of (*E*)-**6**.

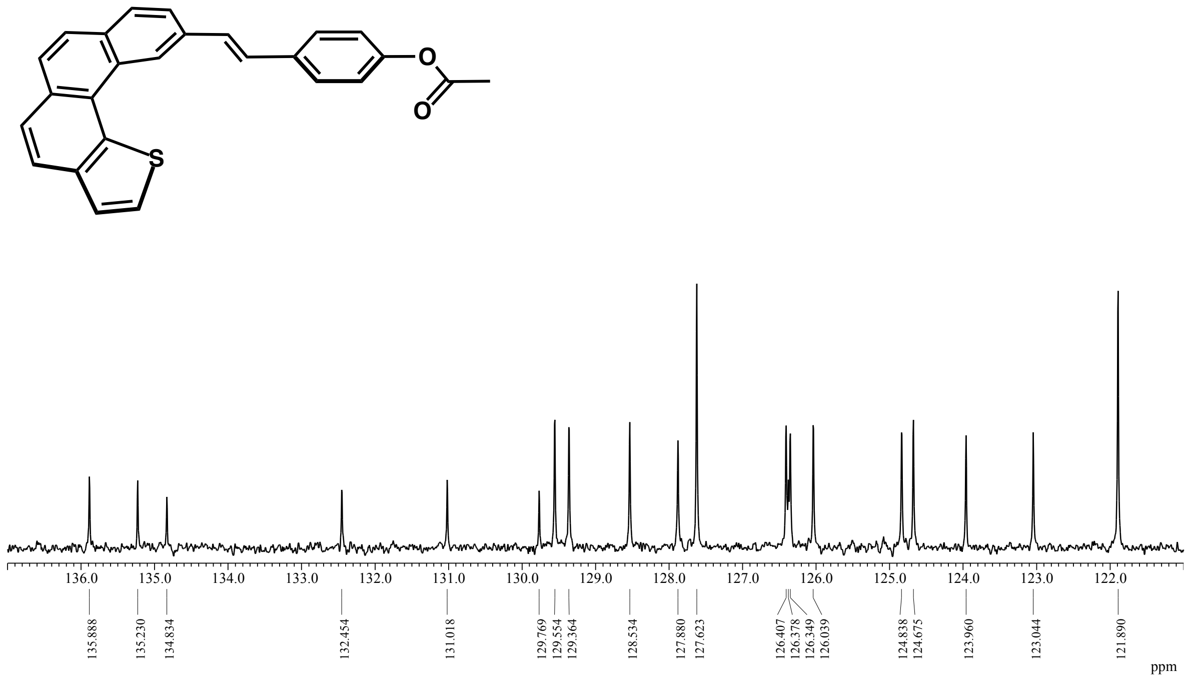

Figure S19. Enlarged $^{13}C\{^{1}H\}$ NMR spectrum (125 MHz, $CDCl_3$, 298 K) of (*E*)-**6**.

Thia[6]helicene **7**

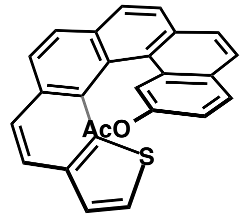

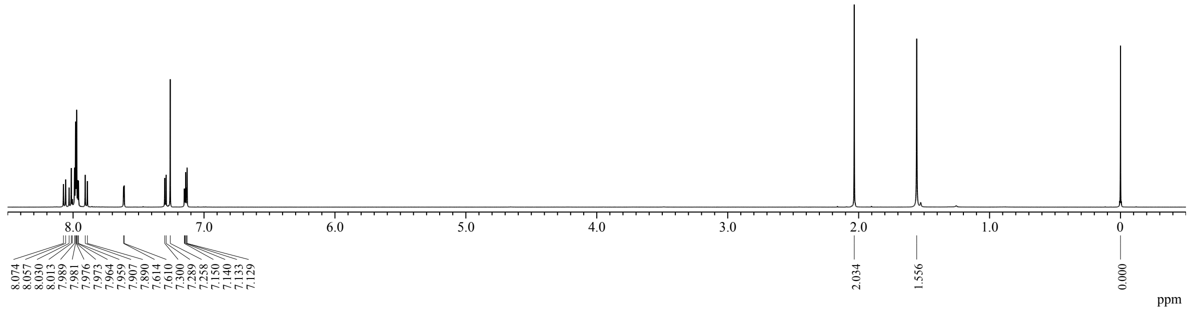

Figure S20. ^{1}H NMR spectrum (500 MHz, $CDCl_3$, 298 K) of **7**.

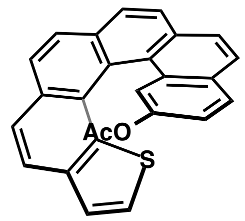

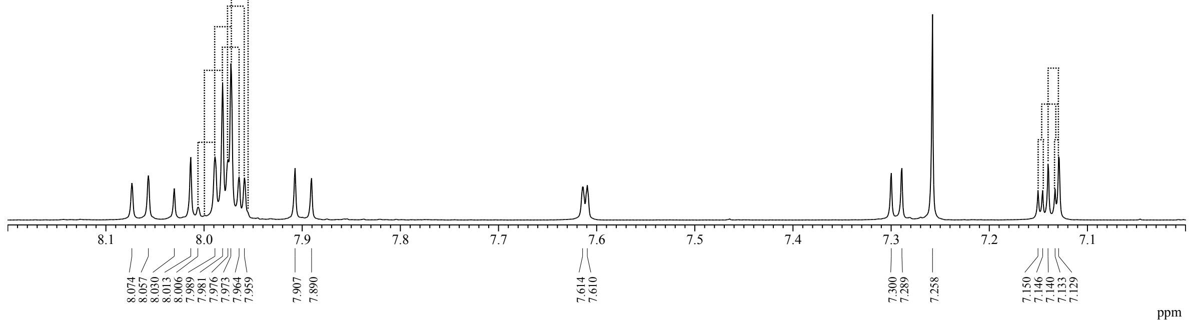

Figure S21. Enlarged ^{1}H NMR spectrum (500 MHz, $CDCl_3$, 298 K) of **7**.

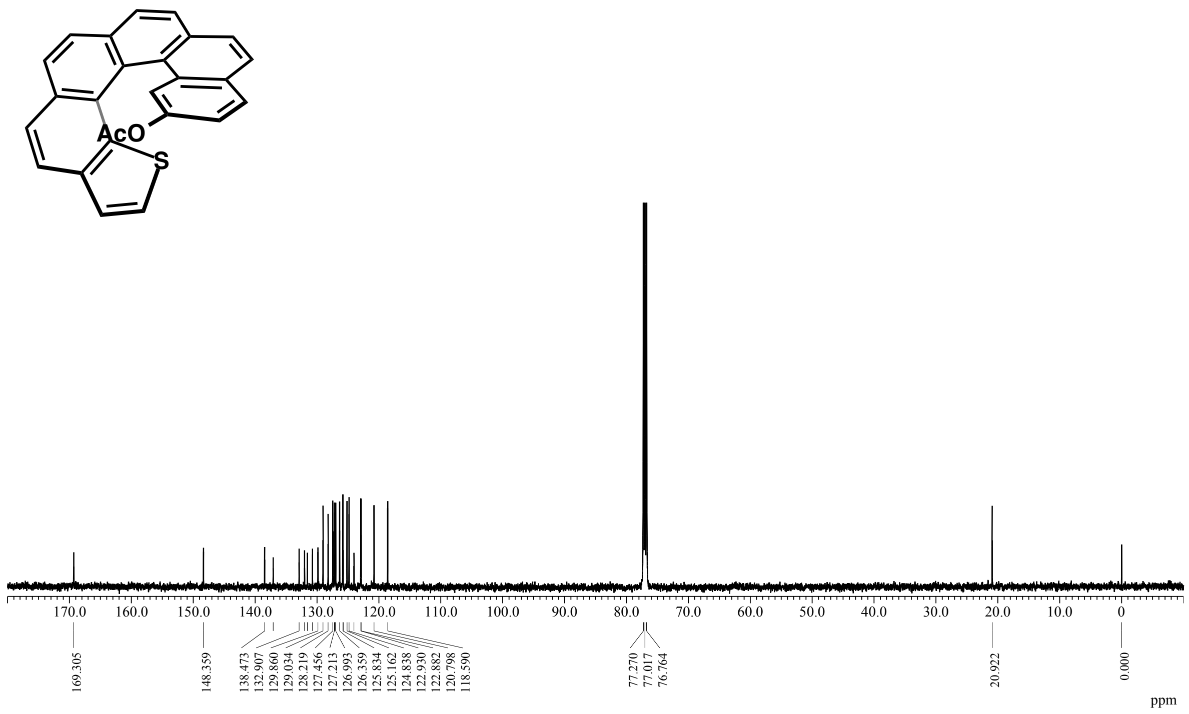

Figure S22. $^{13}C\{^{1}H\}$ NMR spectrum (125 MHz, $CDCl_3$, 298 K) of **7**.

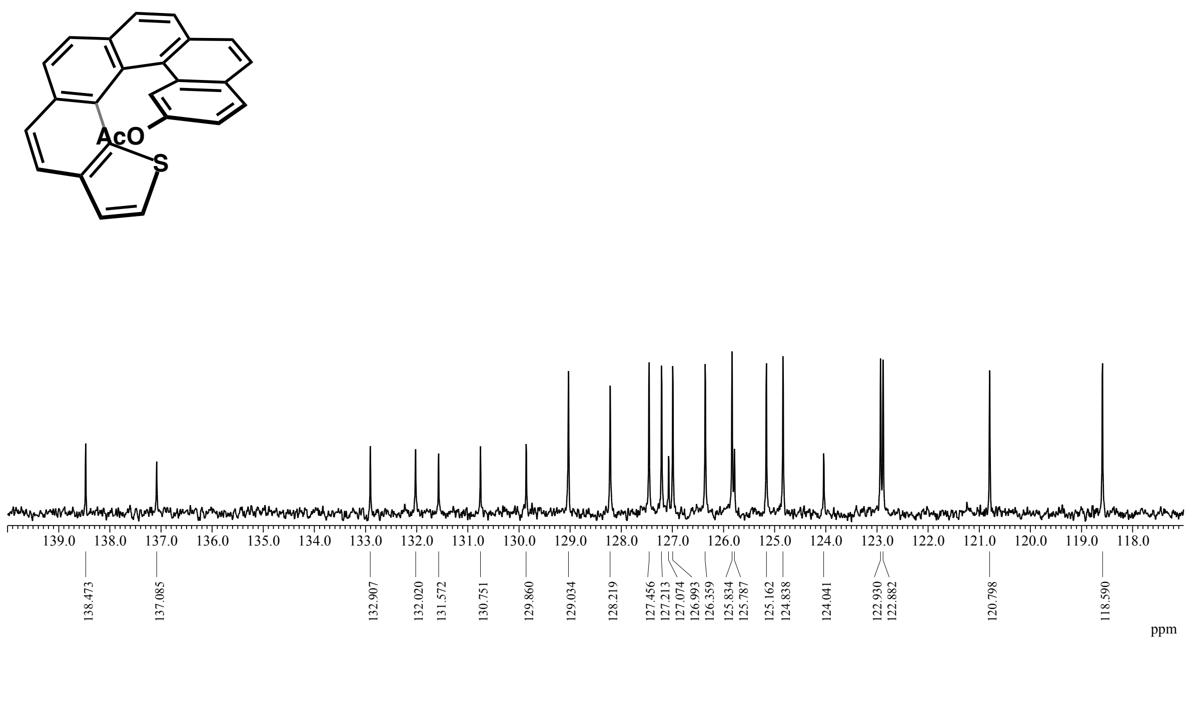

Figure S23. Enlarged $^{13}C\{^{1}H\}$ NMR spectrum (125 MHz, $CDCl_3$, 298 K) of **7**.

Thia[6]helicene *S,S*-dioxide **1d**

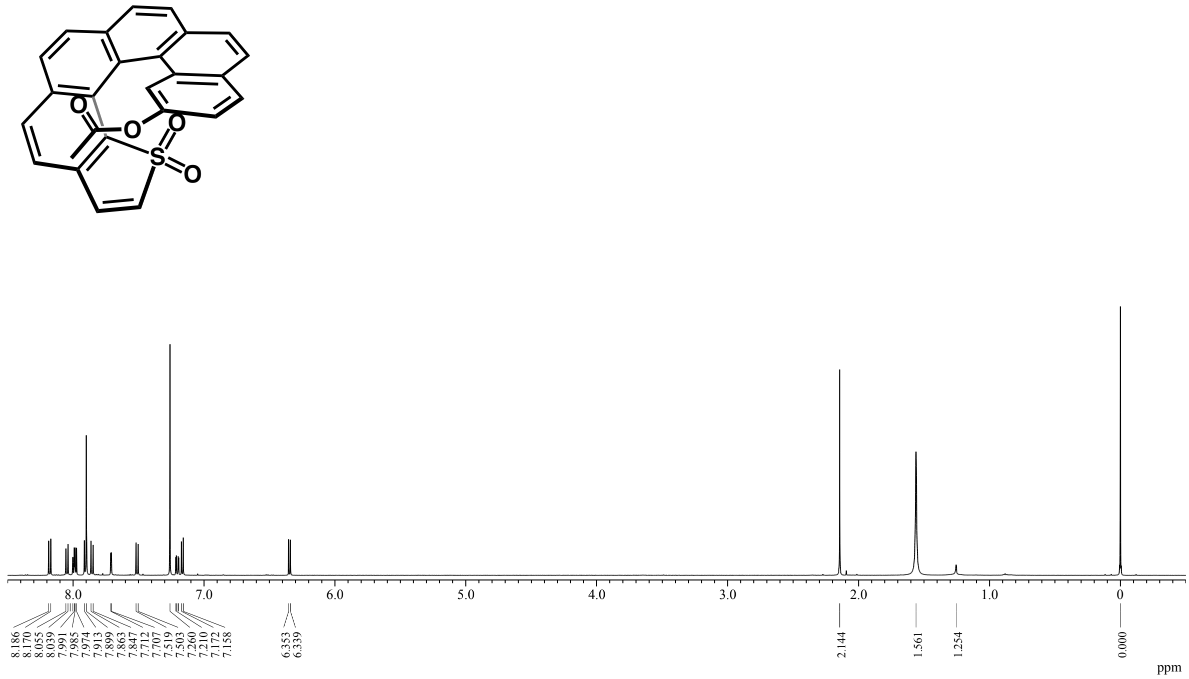

Figure S24. ^{1}H NMR spectrum (500 MHz, $CDCl_3$, 298 K) of **1d**.

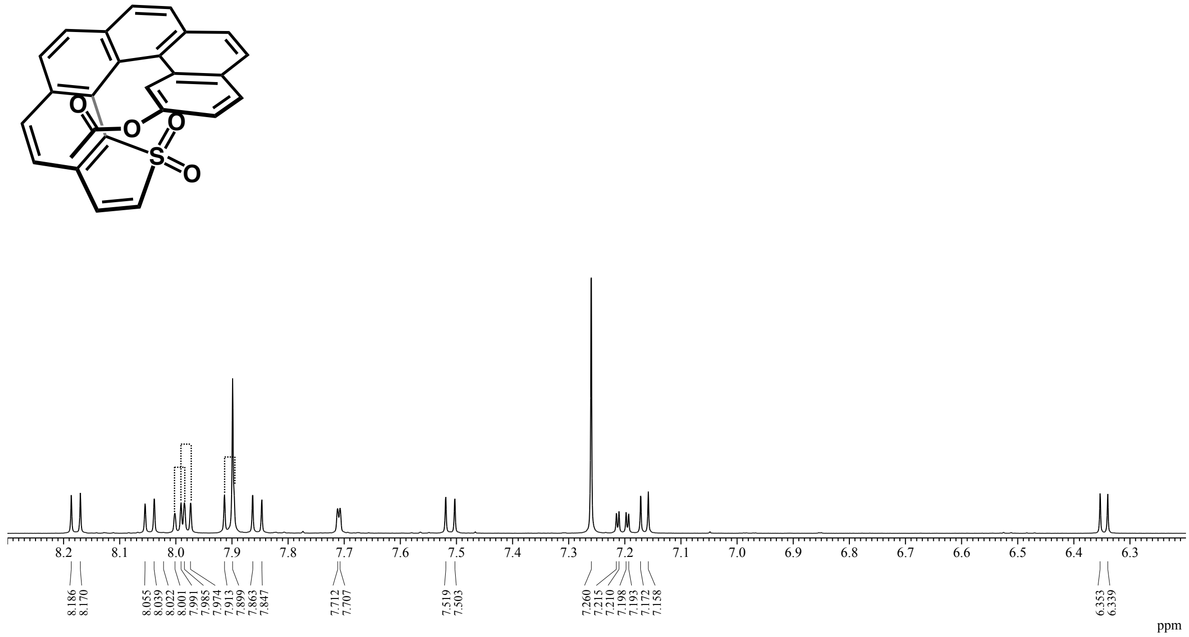

Figure S25. Enlarged ^{1}H NMR spectrum (500 MHz, $CDCl_3$, 298 K) of **1d**.

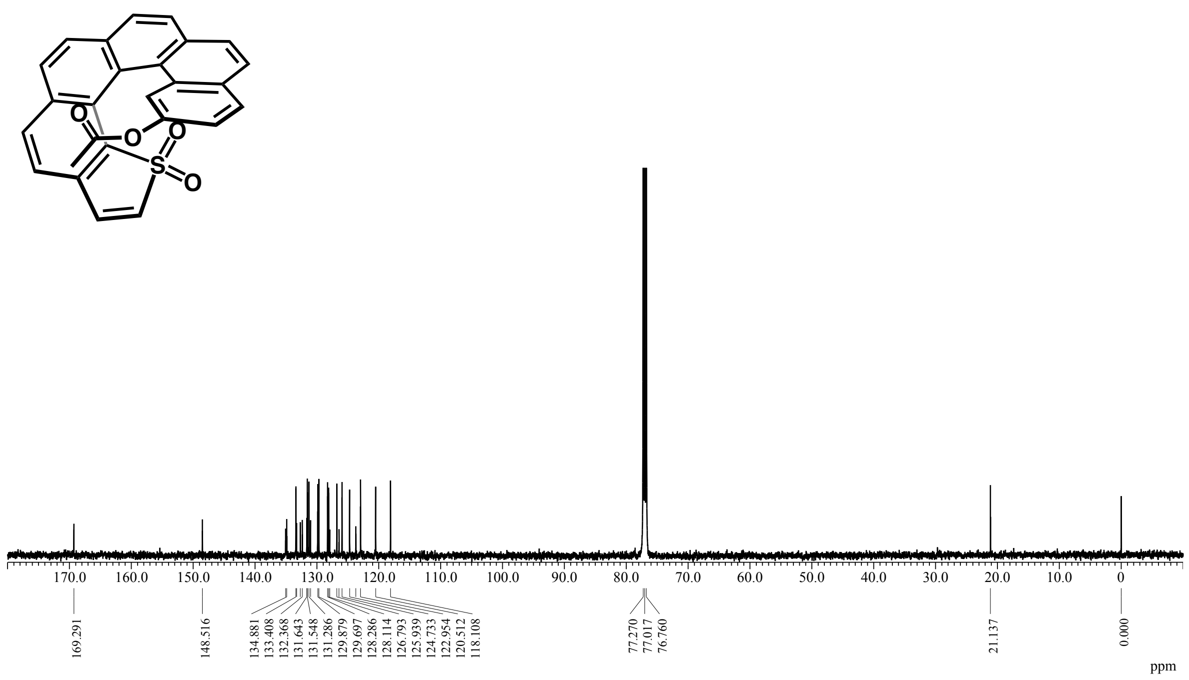

Figure S26. $^{13}C\{^{1}H\}$ NMR spectrum (125 MHz, $CDCl_3$, 298 K) of **1d**.

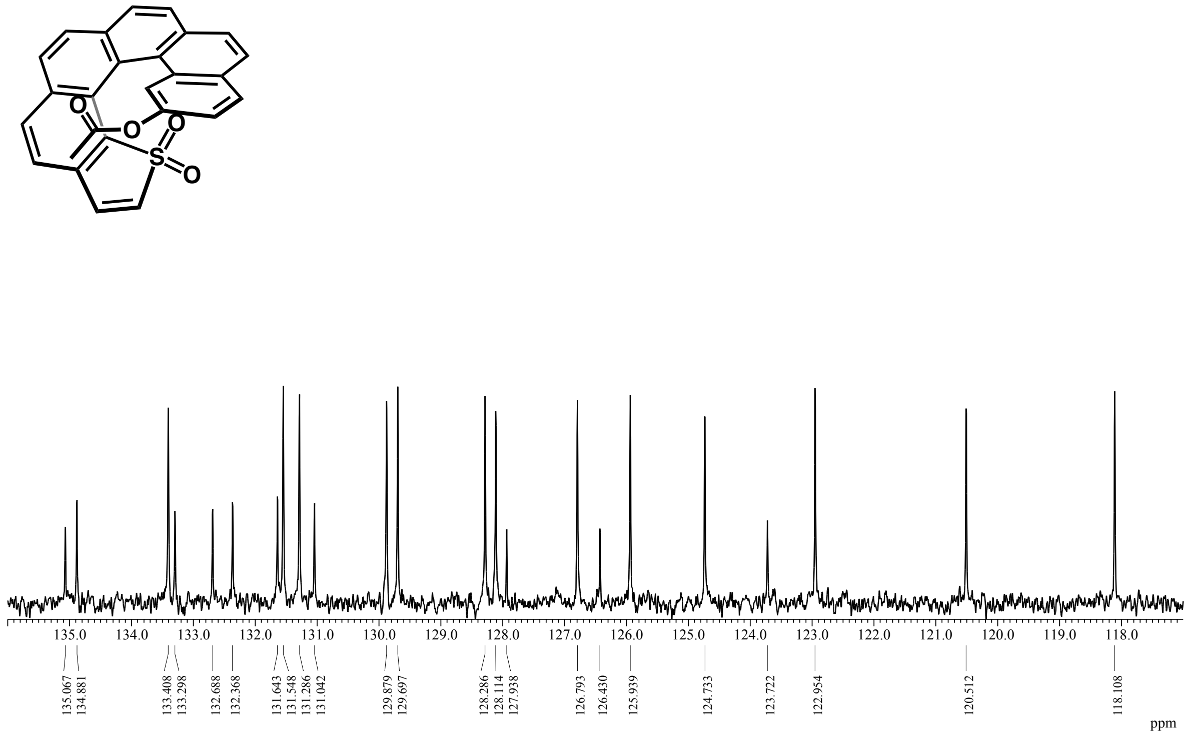

Figure S27. Enlarged $^{13}C\{^{1}H\}$ NMR spectrum (125 MHz, $CDCl_3$, 298 K) of **1d**.

Thia[6]helicene *S*,*S*-dioxide **1c**

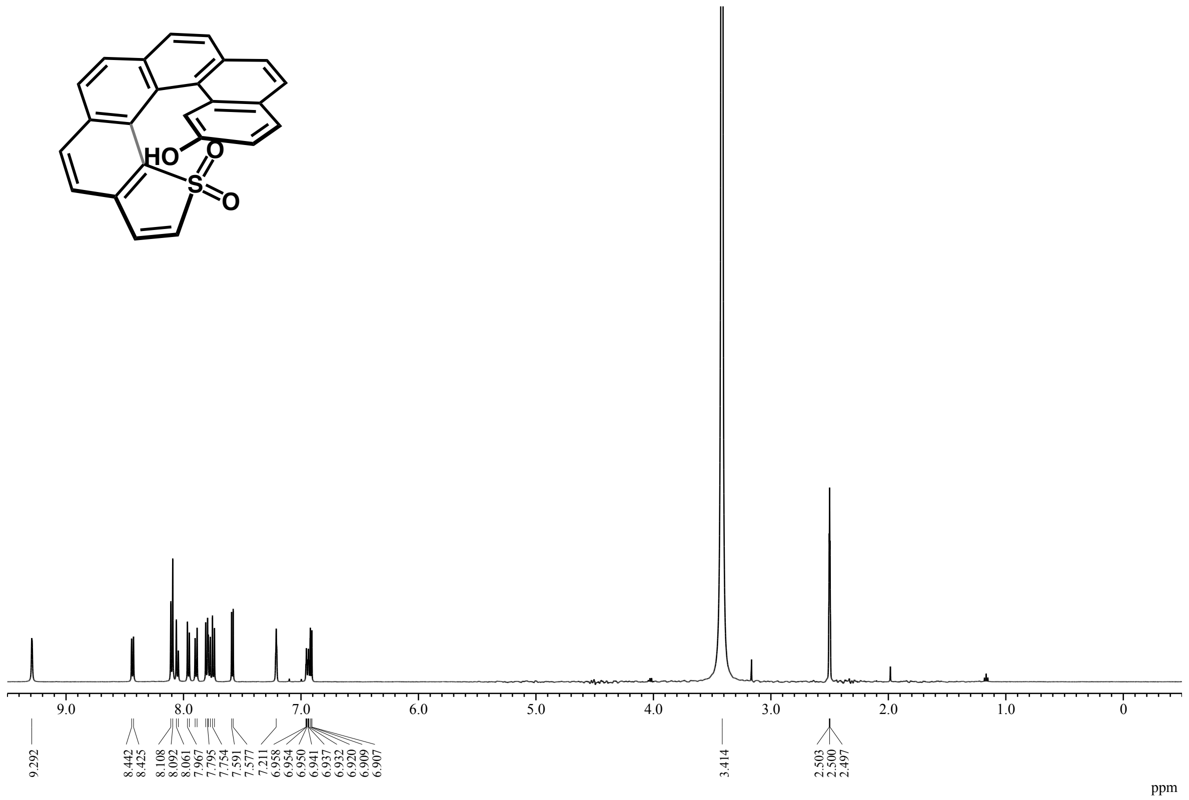

Figure S28. ^{1}H NMR spectrum (500 MHz, DMSO-d_6, 298 K) of **1c**.

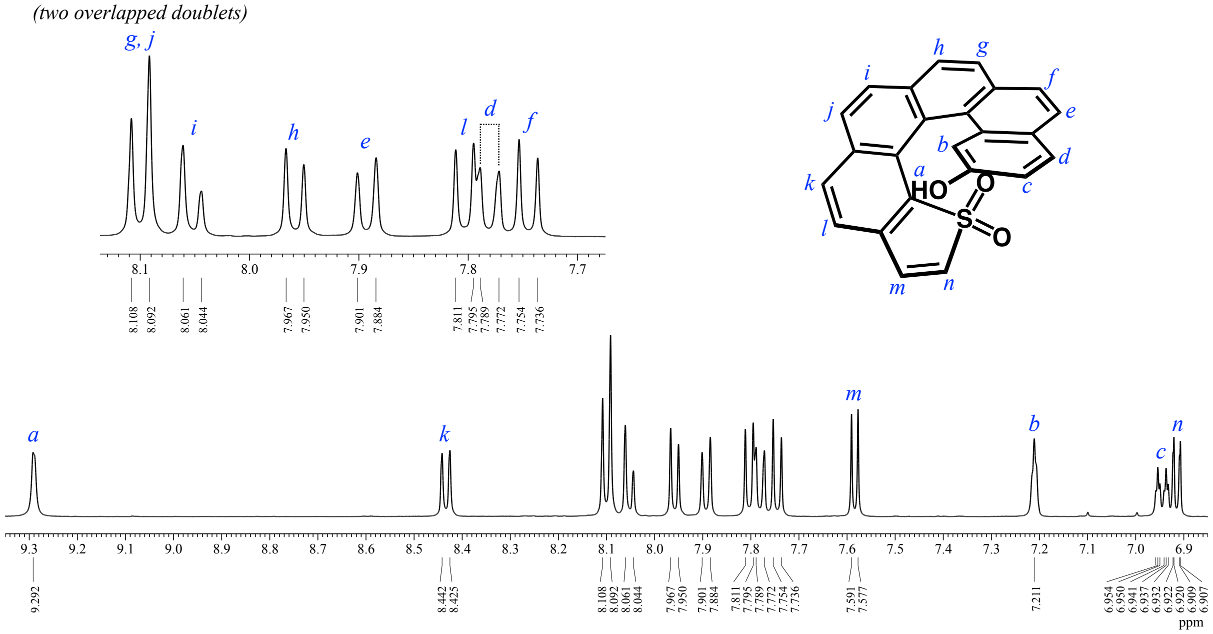

Figure S29. Enlarged ^{1}H NMR spectrum (500 MHz, DMSO-d_6, 298 K) of **1c**.

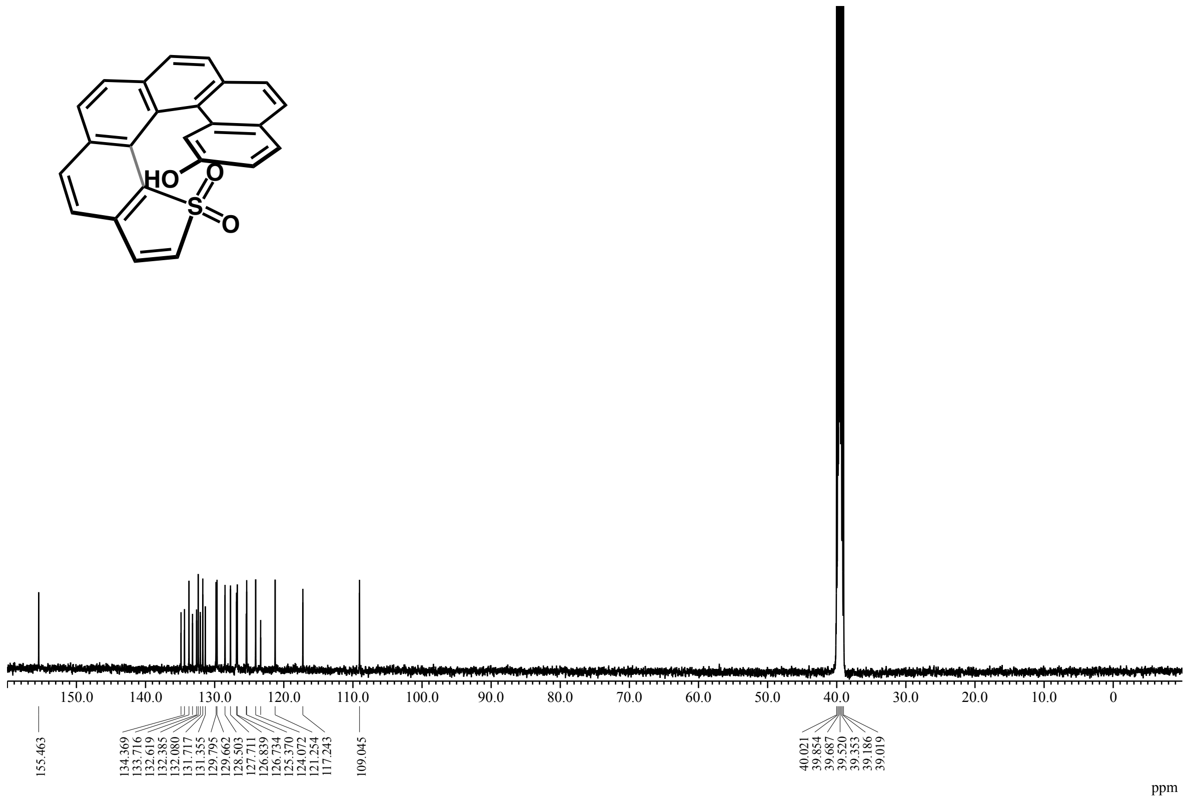

Figure S30. $^{13}C\{^{1}H\}$ NMR spectrum (125 MHz, DMSO-d_6, 298 K) of **1c**.

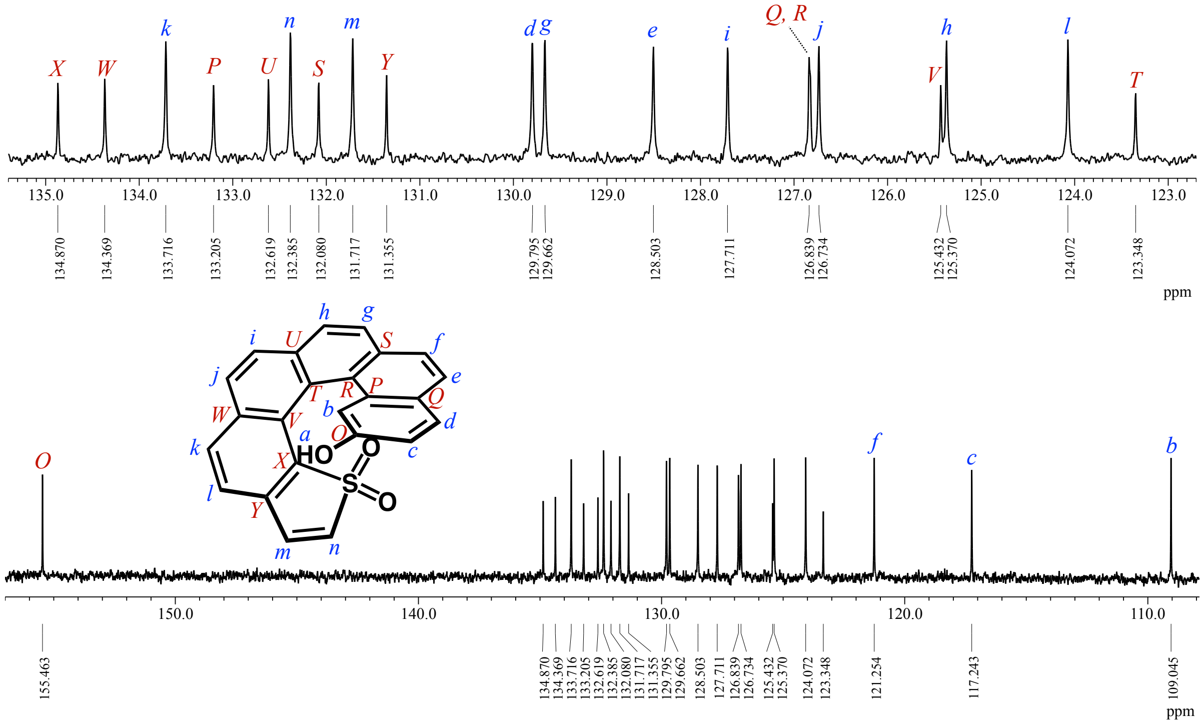

Figure S31. Enlarged $^{13}C\{^{1}H\}$ NMR spectrum (125 MHz, DMSO-d_6, 298 K) of **1c**.

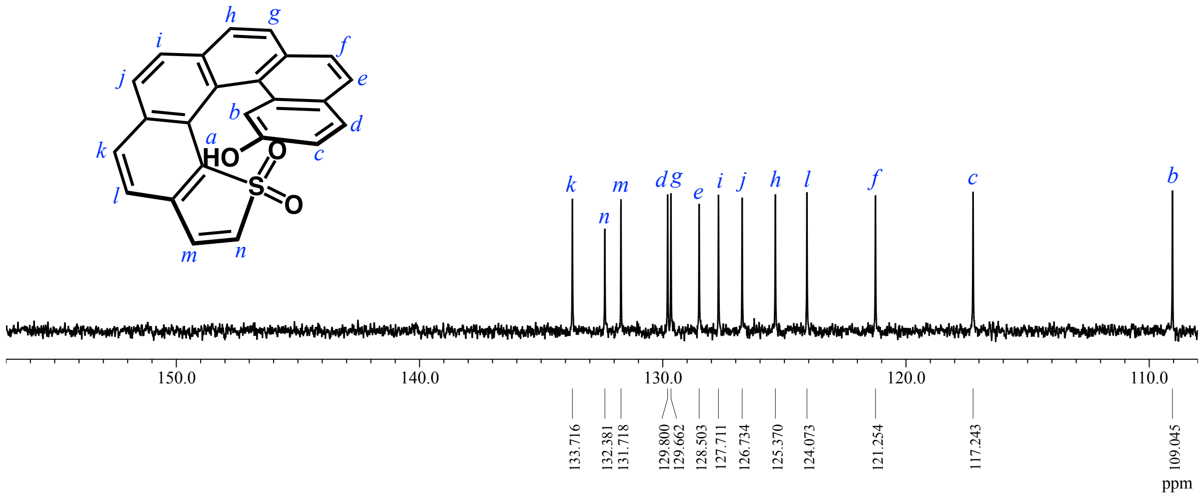

Figure S32. ^{13}C DEPT-135 NMR spectrum (125 MHz, DMSO-d_6, 298 K) of **1c**.

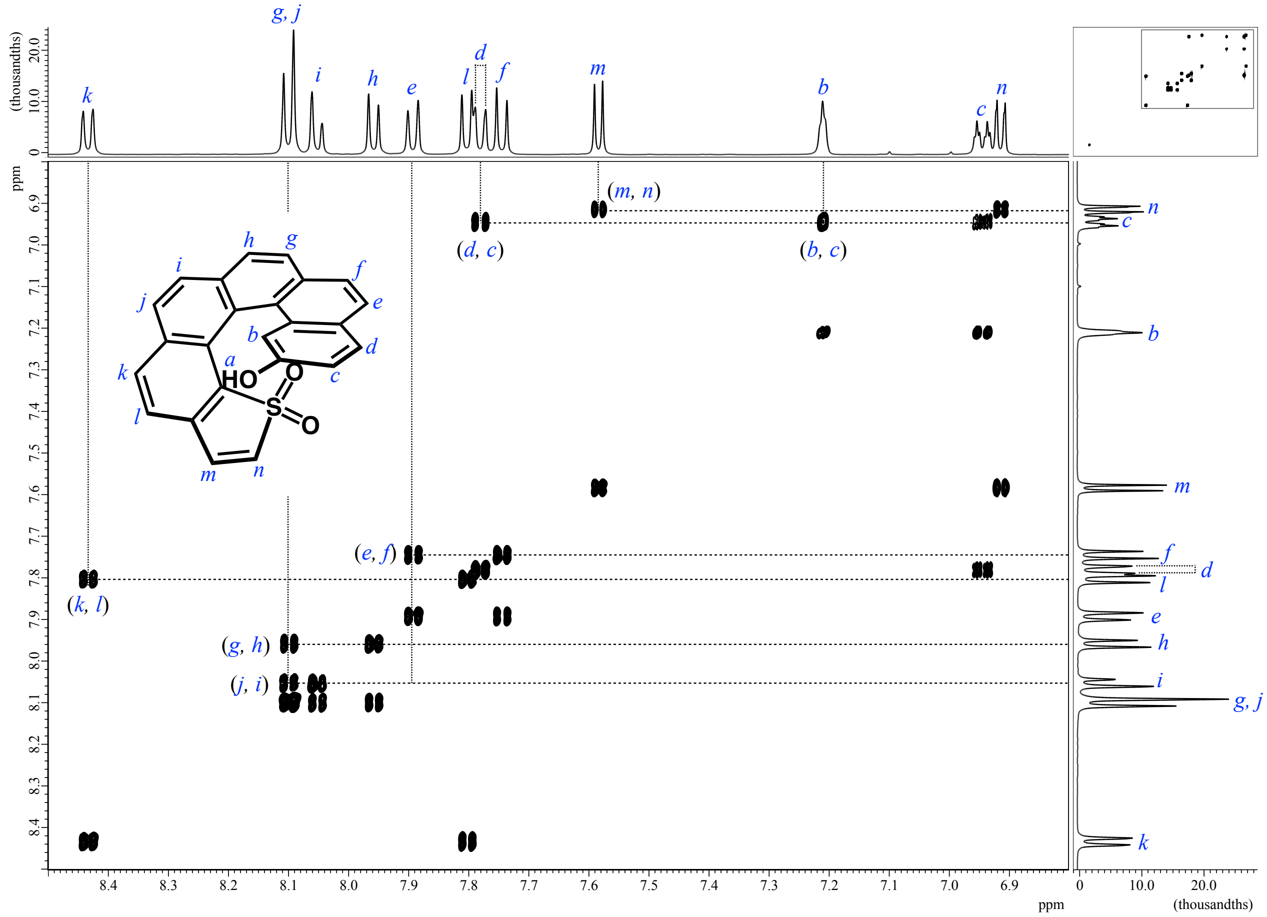

Figure S33. ^{1}H–^{1}H COSY NMR spectrum (500 MHz, DMSO-d_6, 298 K) of **1c**.

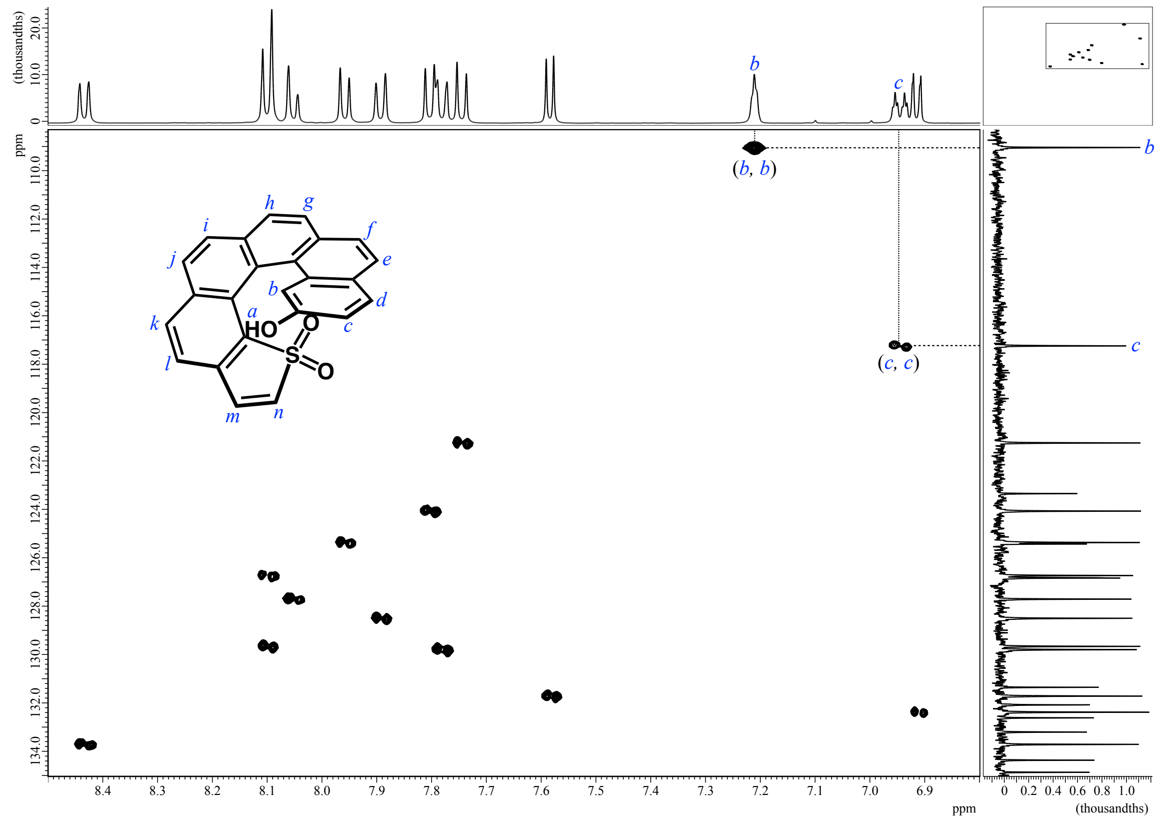

Figure S34. ^{1}H–^{13}C HMQC NMR spectrum (500 MHz, DMSO-d_6, 298 K) of **1c**.

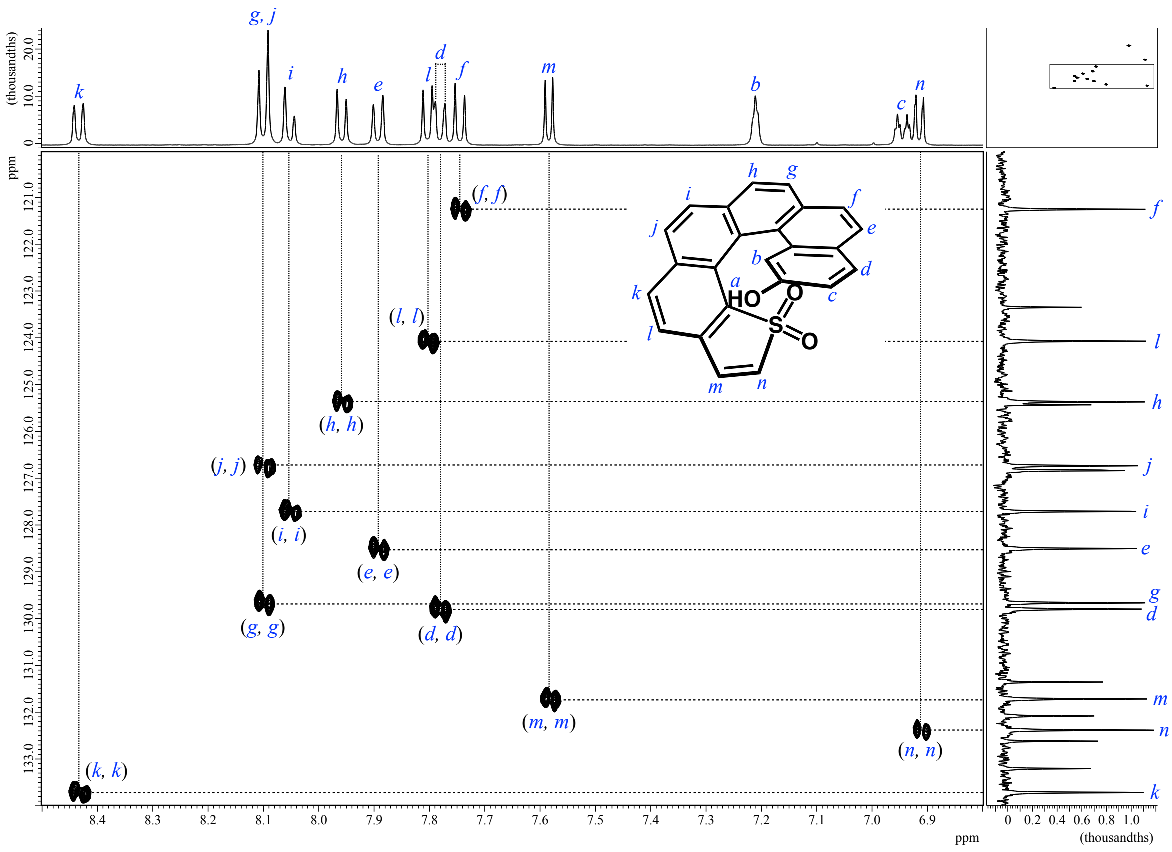

Figure S35. Enlarged ^{1}H–^{13}C HMQC NMR spectrum (500 MHz, DMSO-d_6, 298 K) of **1c**.

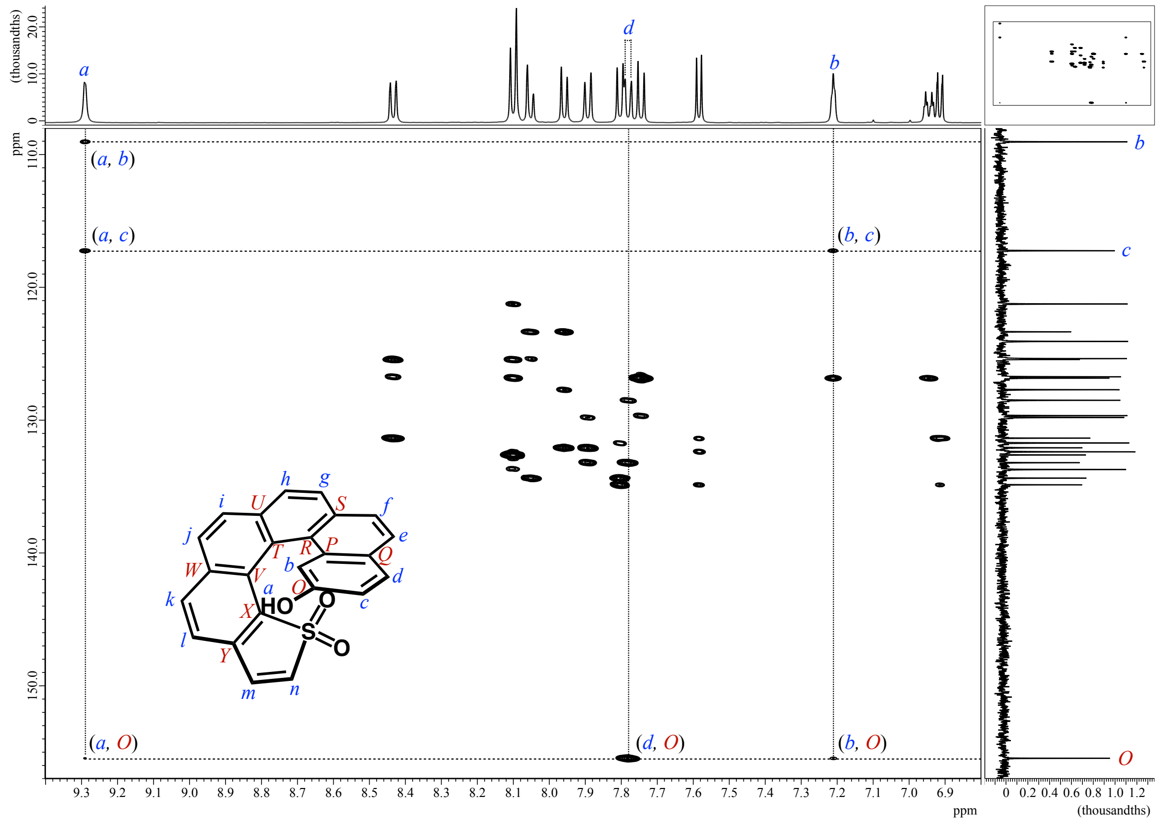

Figure S36. ^{1}H–^{13}C HMBC NMR spectrum (500 MHz, DMSO-d_6, 298 K) of **1c**.

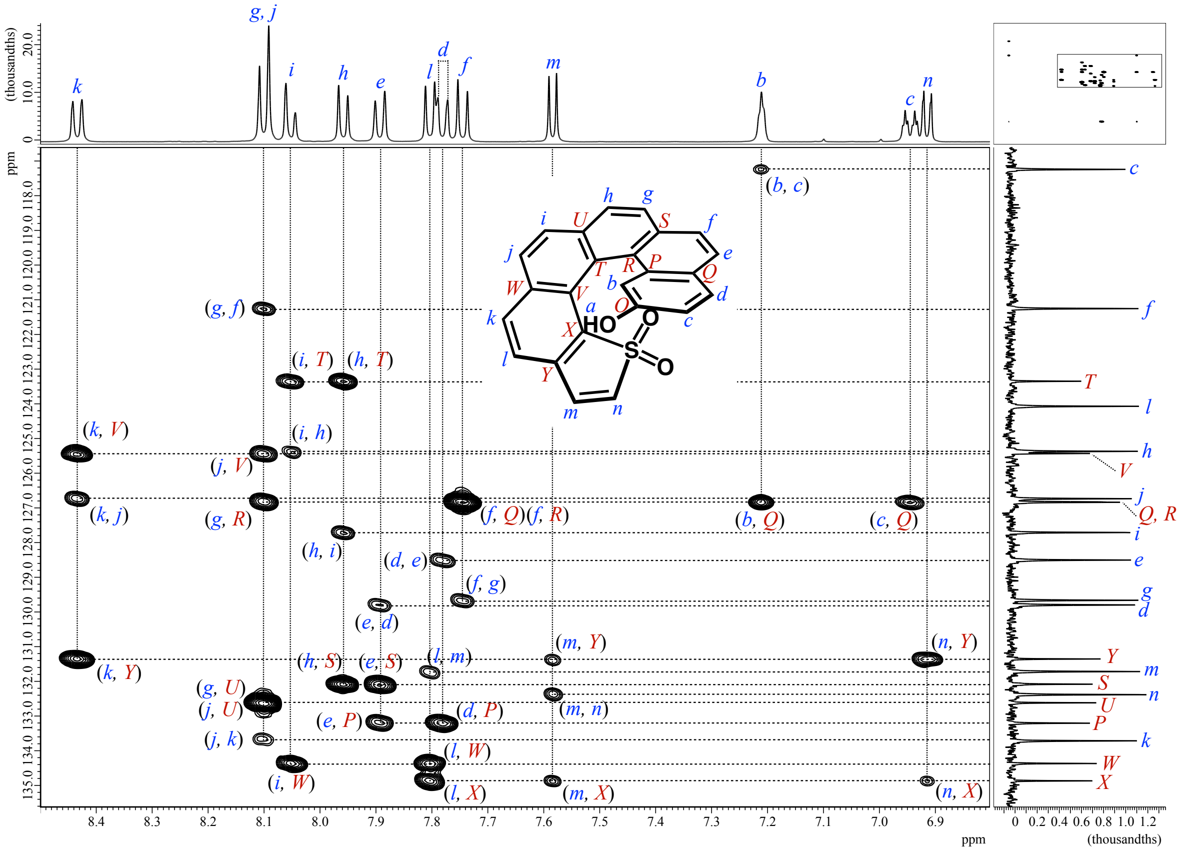

Figure S37. Enlarged ^{1}H–^{13}C HMBC NMR spectrum (500 MHz, DMSO-d_6, 298 K) of **1c**.

Oxa-Michael adduct **1c'**(α)

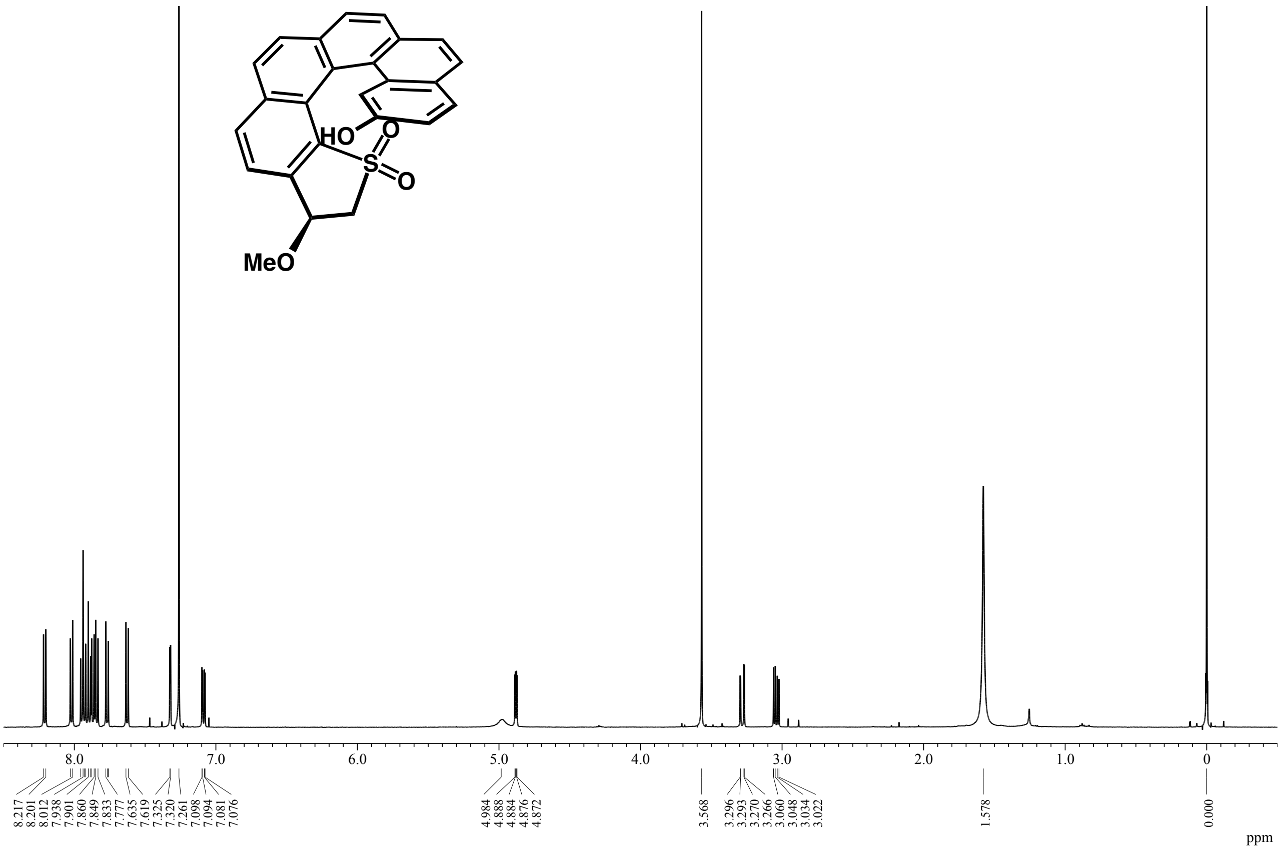

Figure S38. ^{1}H NMR spectrum (500 MHz, $CDCl_3$, 298 K) of **1c'**(α).

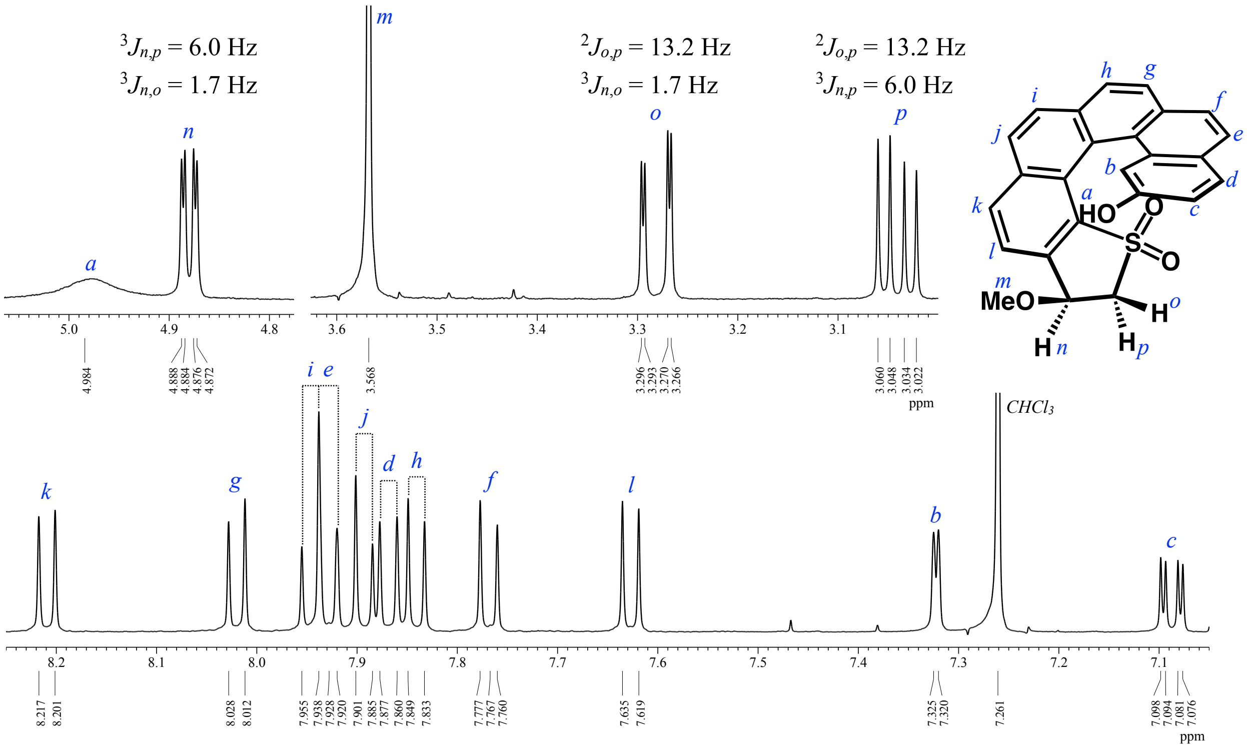

Figure S39. Enlarged ^{1}H NMR spectrum (500 MHz, $CDCl_3$, 298 K) of **1c'**(α).

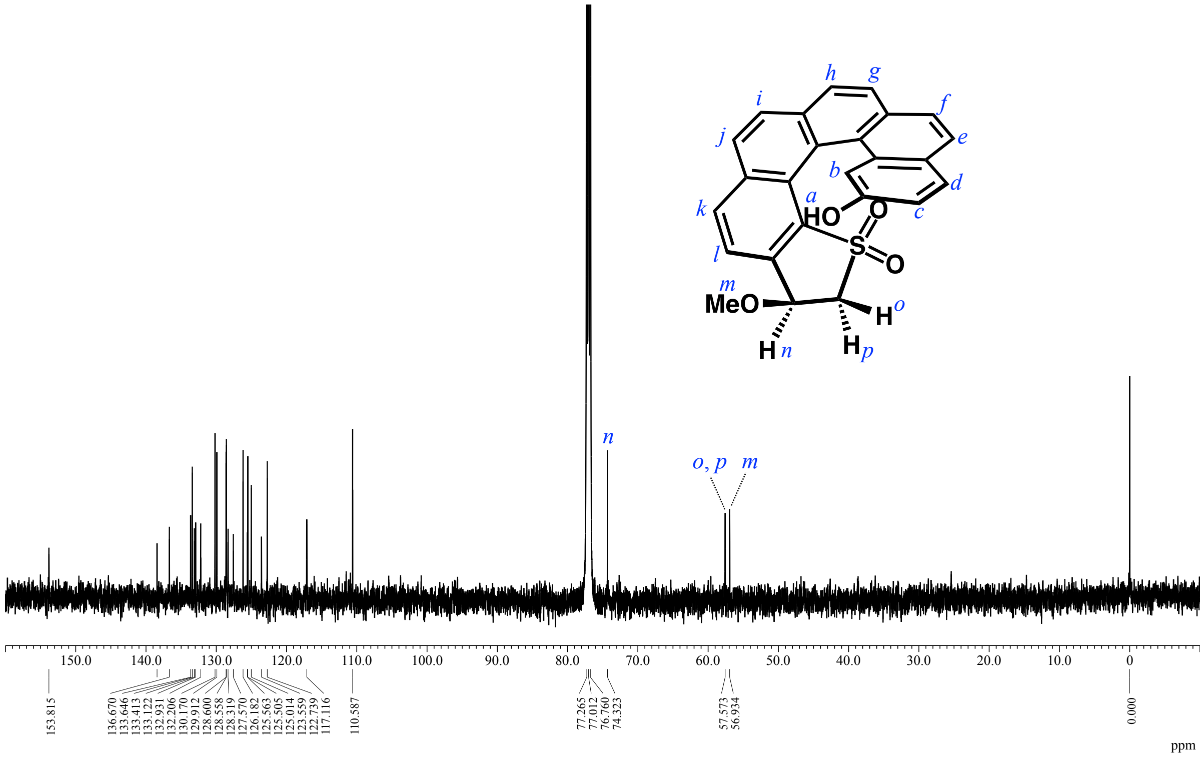

Figure S40. $^{13}C\{^{1}H\}$ NMR spectrum (125 MHz, $CDCl_3$, 298 K) of **1c'**(α).

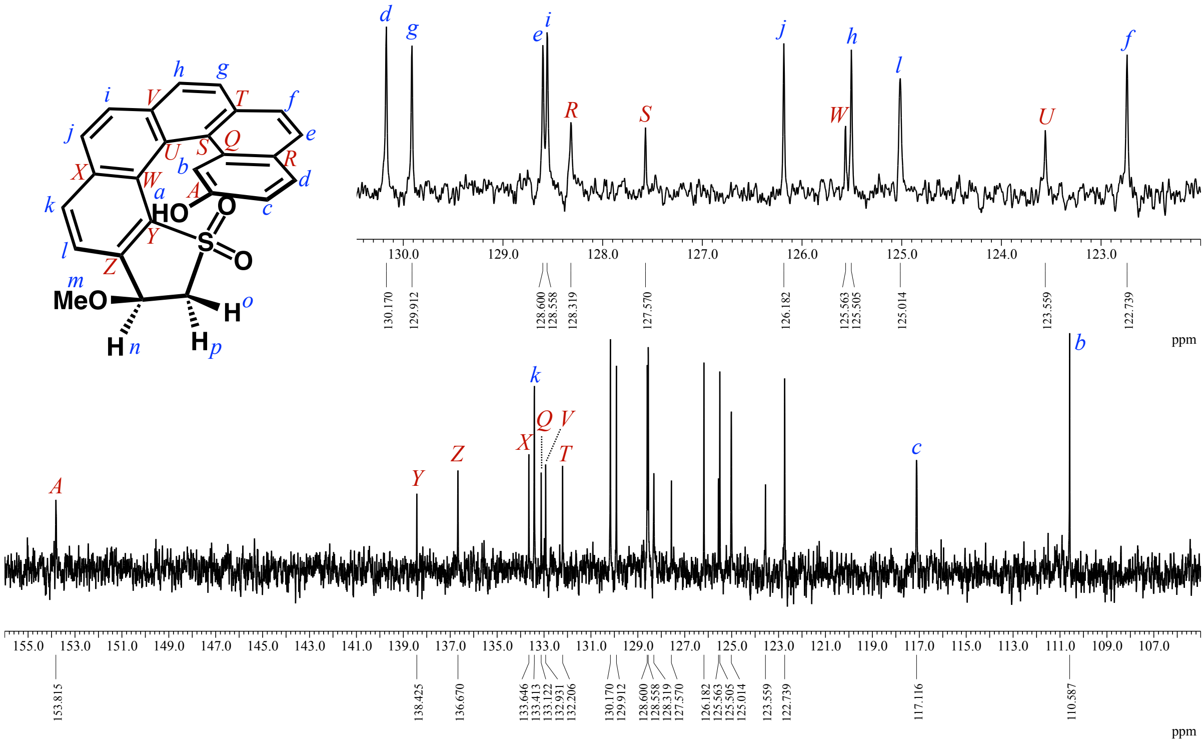

Figure S41. Enlarged $^{13}C\{^{1}H\}$ NMR spectrum (125 MHz, $CDCl_3$, 298 K) of **1c'**(α).

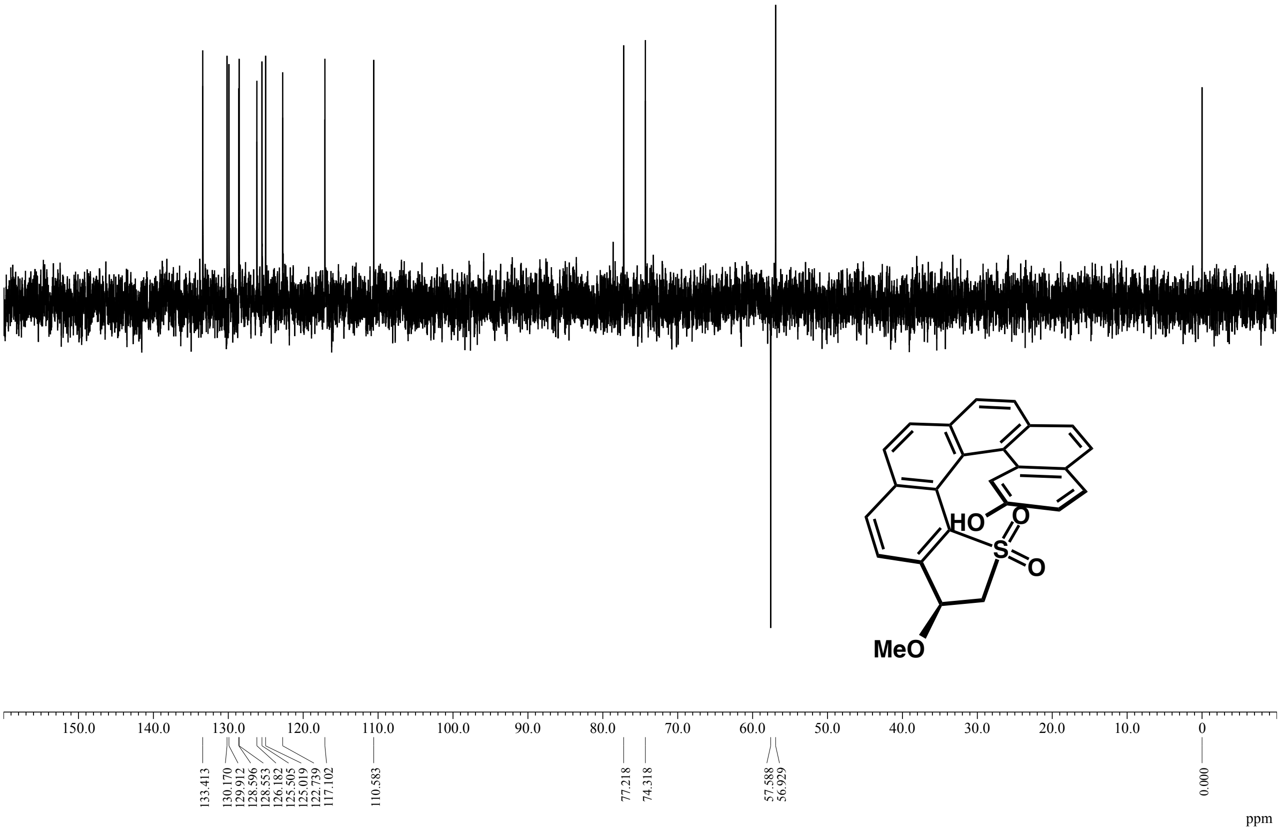

Figure S42. ^{13}C DEPT-135 NMR spectrum (125 MHz, $CDCl_3$, 298 K) of **1c'**(α).

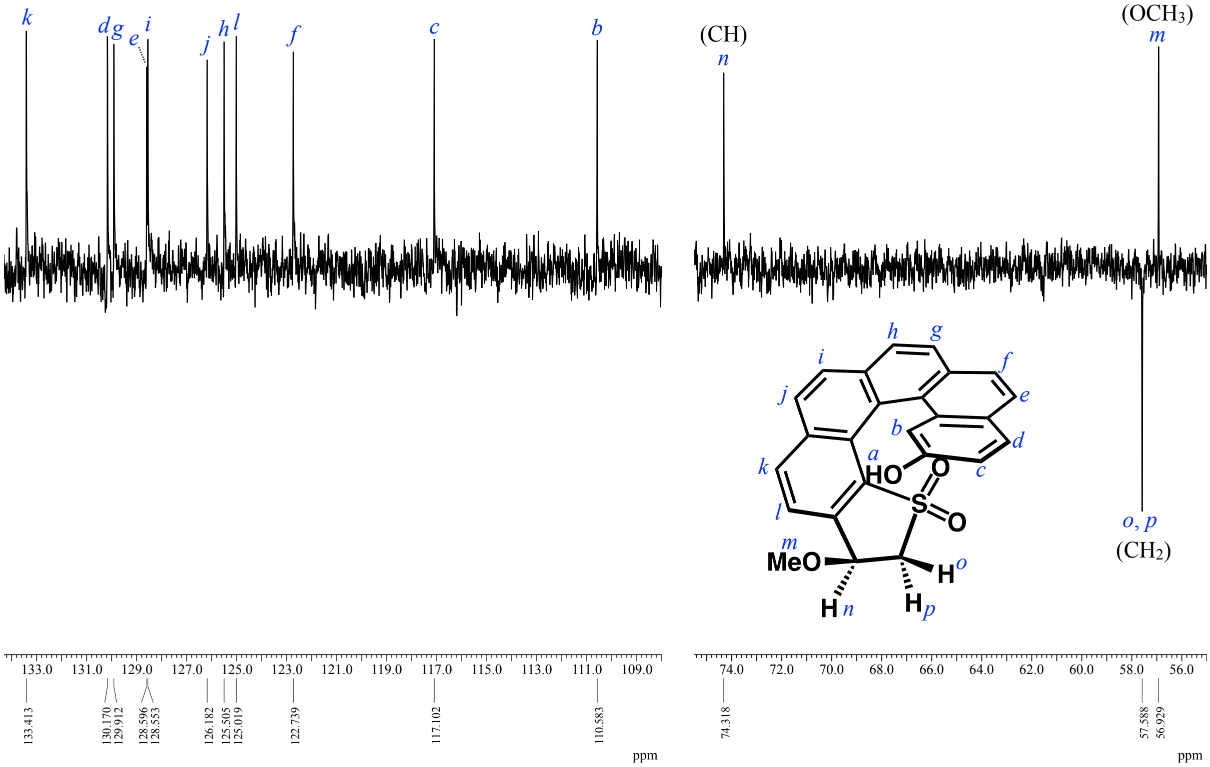

Figure S43. Enlarged ^{13}C DEPT-135 NMR spectrum (125 MHz, $CDCl_3$, 298 K) of **1c'**(α).

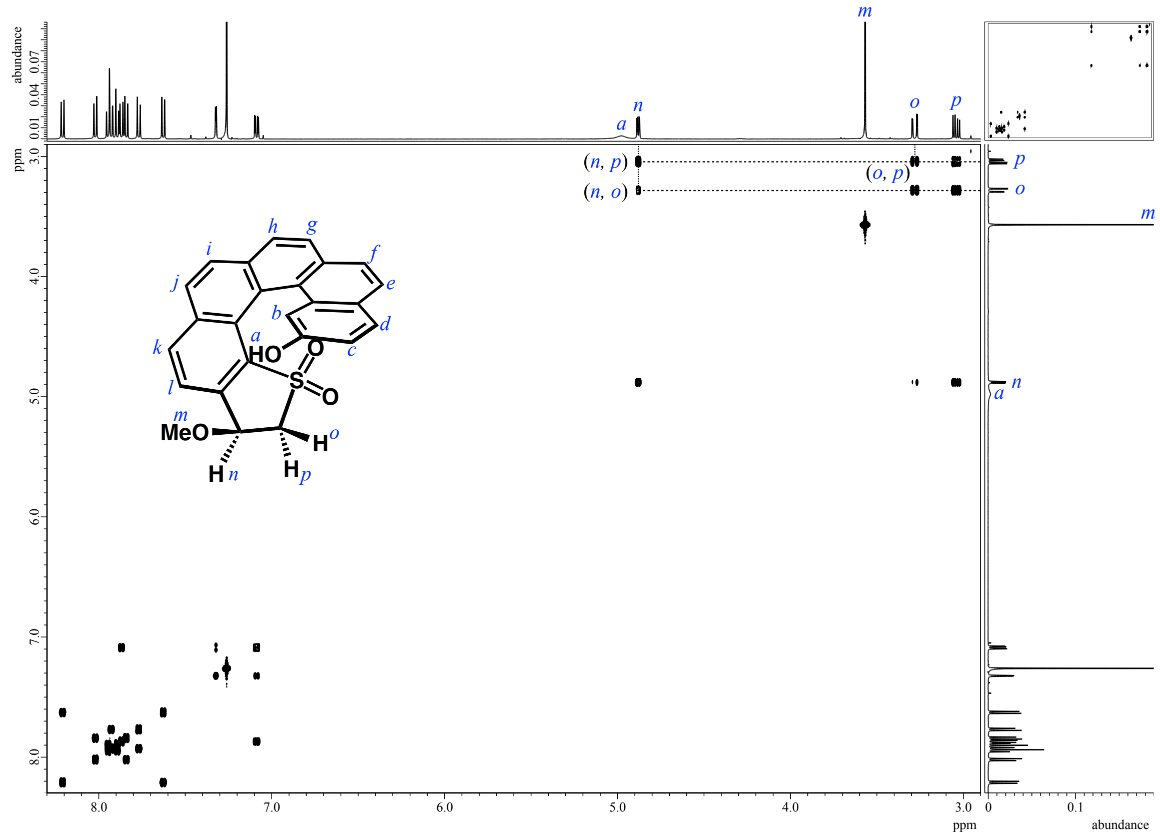

Figure S44. ^{1}H–^{1}H COSY NMR spectrum (500 MHz, $CDCl_3$, 298 K) of **1c'**(α).

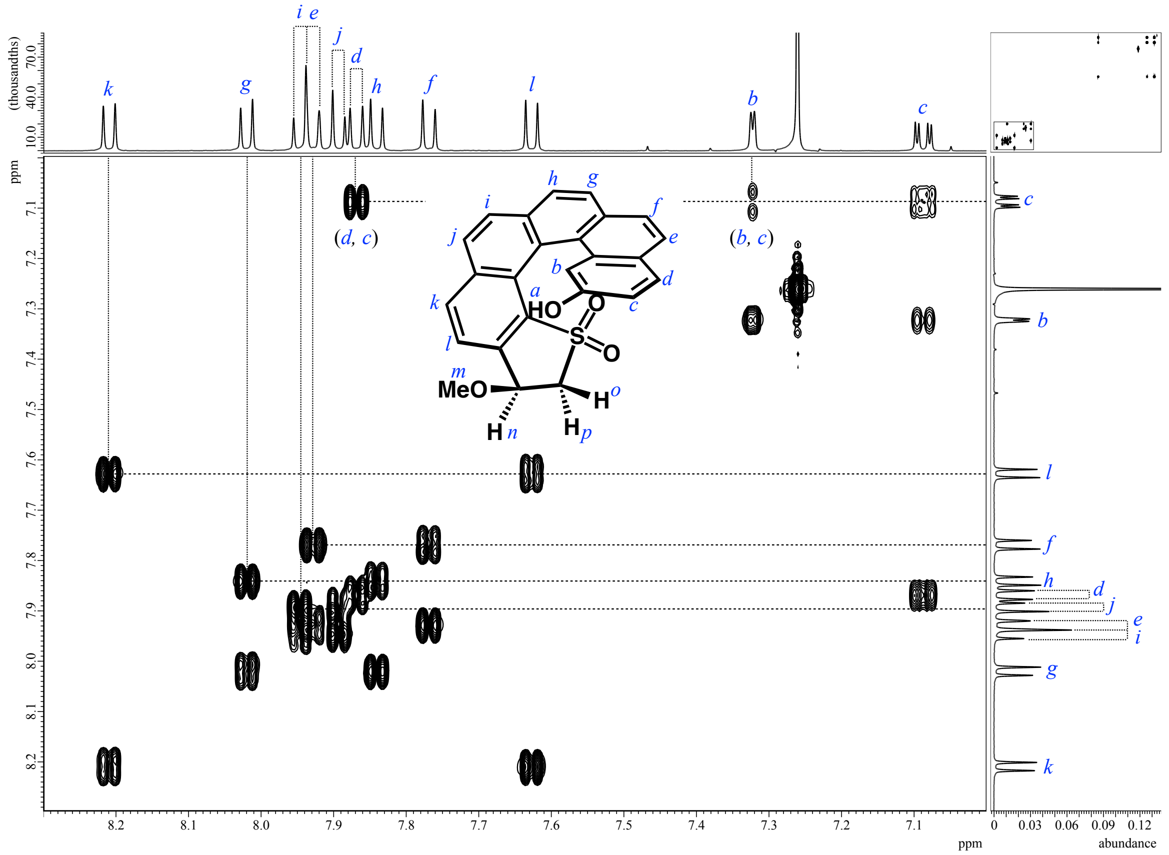

Figure S45. Enlarged ^{1}H–^{1}H COSY NMR spectrum (500 MHz, $CDCl_3$, 298 K) of **1c'**(α).

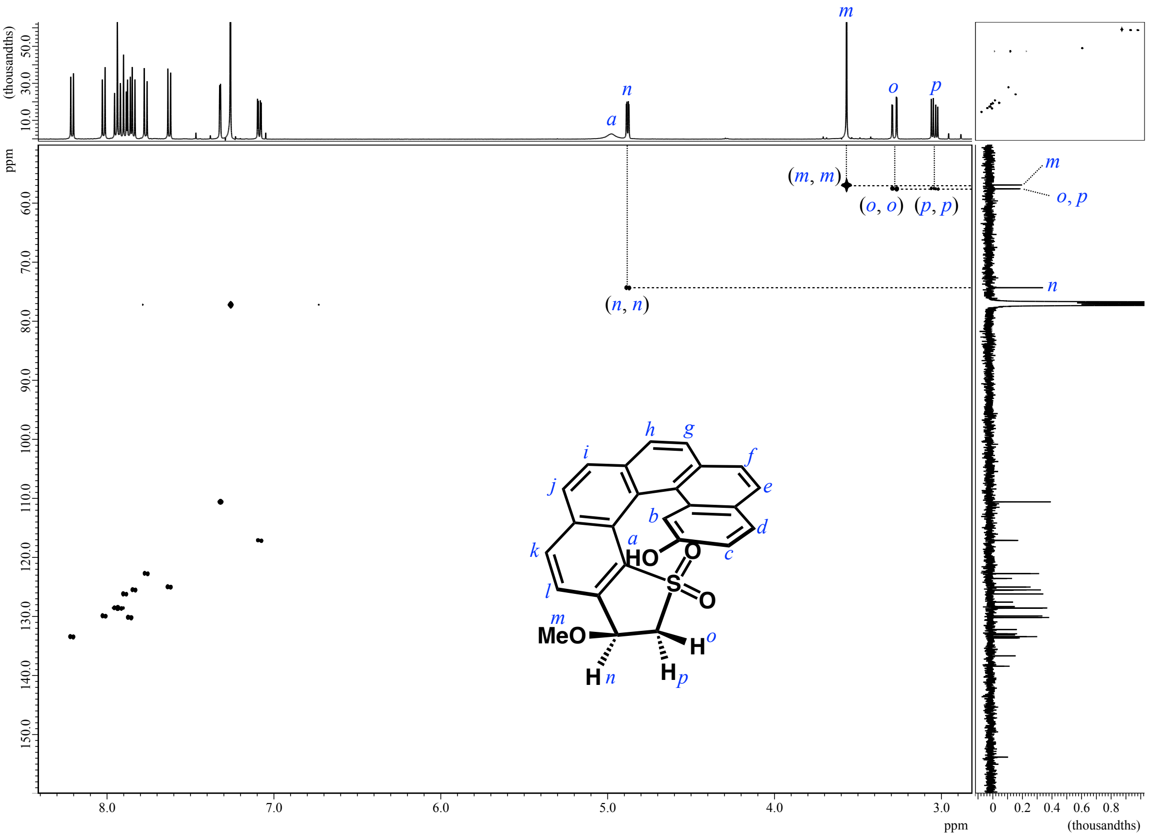

Figure S46. ^{1}H–^{13}C HMQC NMR spectrum (500 MHz, $CDCl_3$, 298 K) of **1c'**(α).

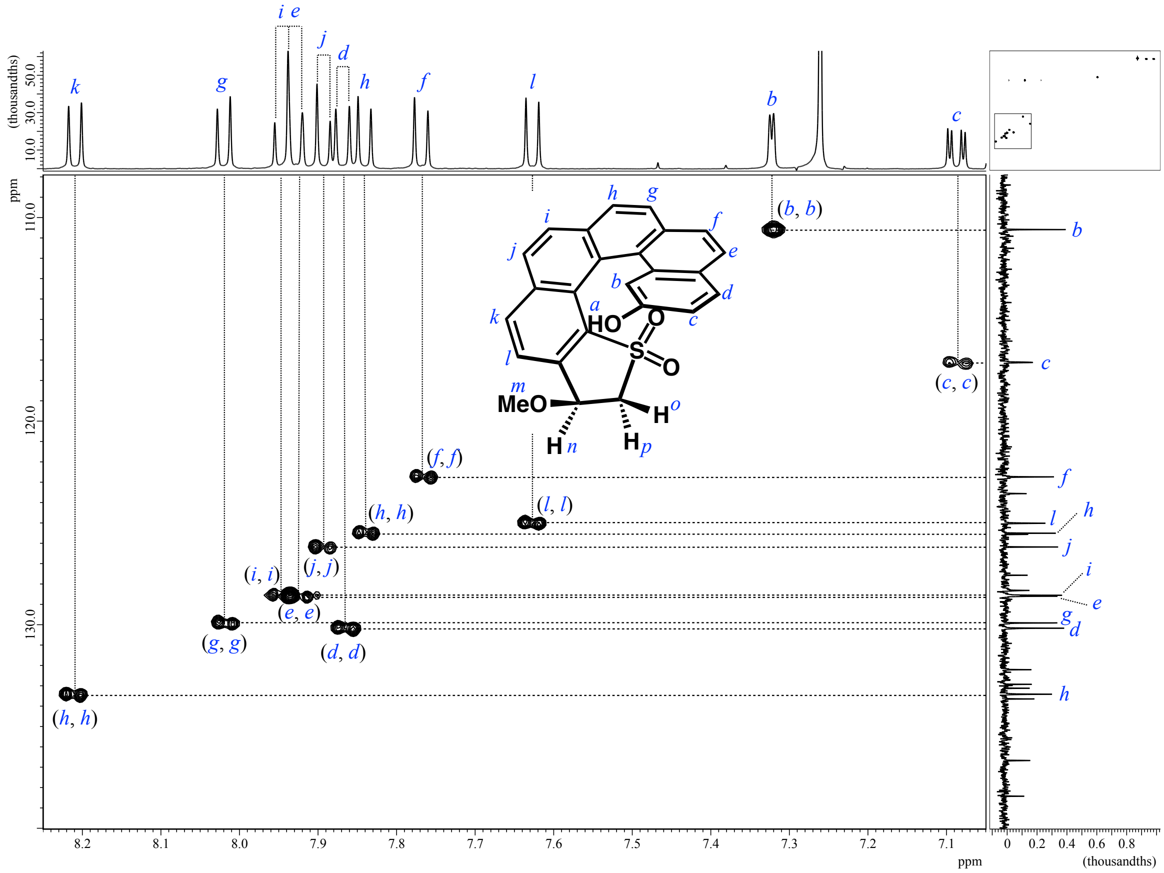

Figure S47. Enlarged ^{1}H–^{13}C HMQC NMR spectrum (500 MHz, $CDCl_3$, 298 K) of **1c'**(α).

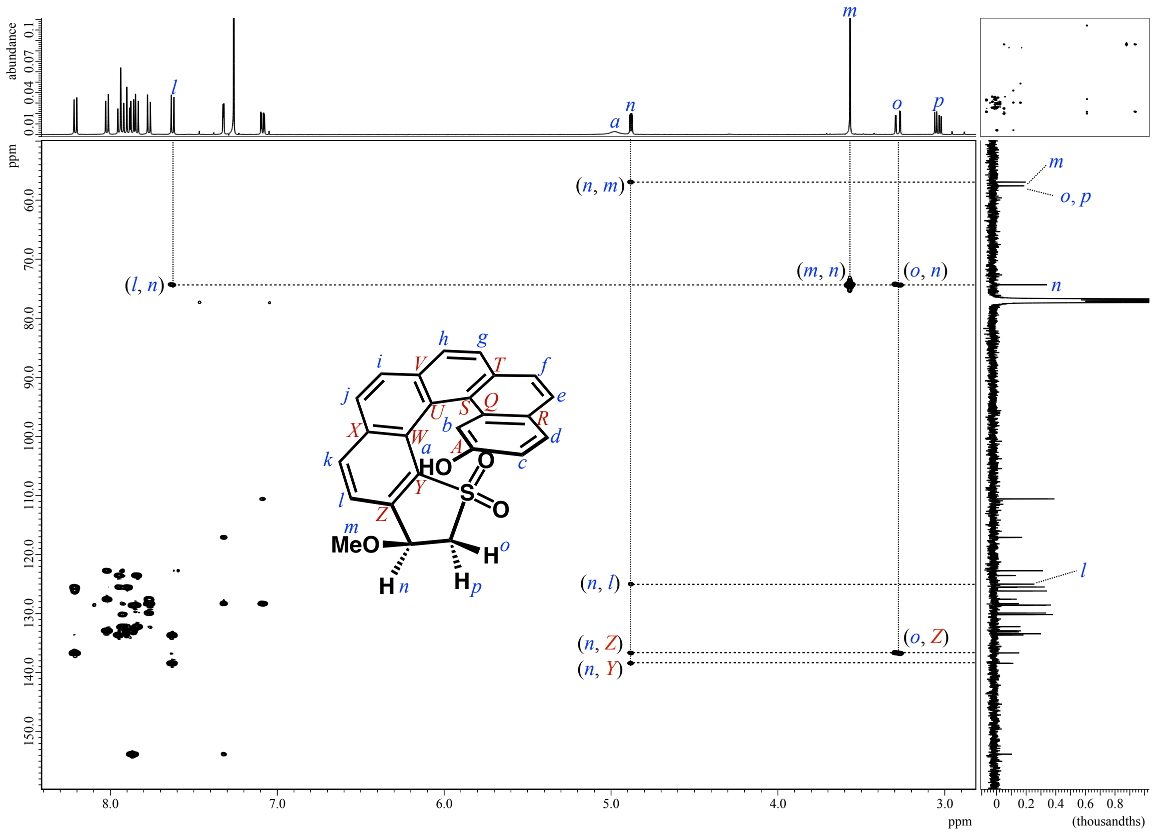

Figure S48. ^{1}H–^{13}C HMBC NMR spectrum (500 MHz, $CDCl_3$, 298 K) of **1c'**(α).

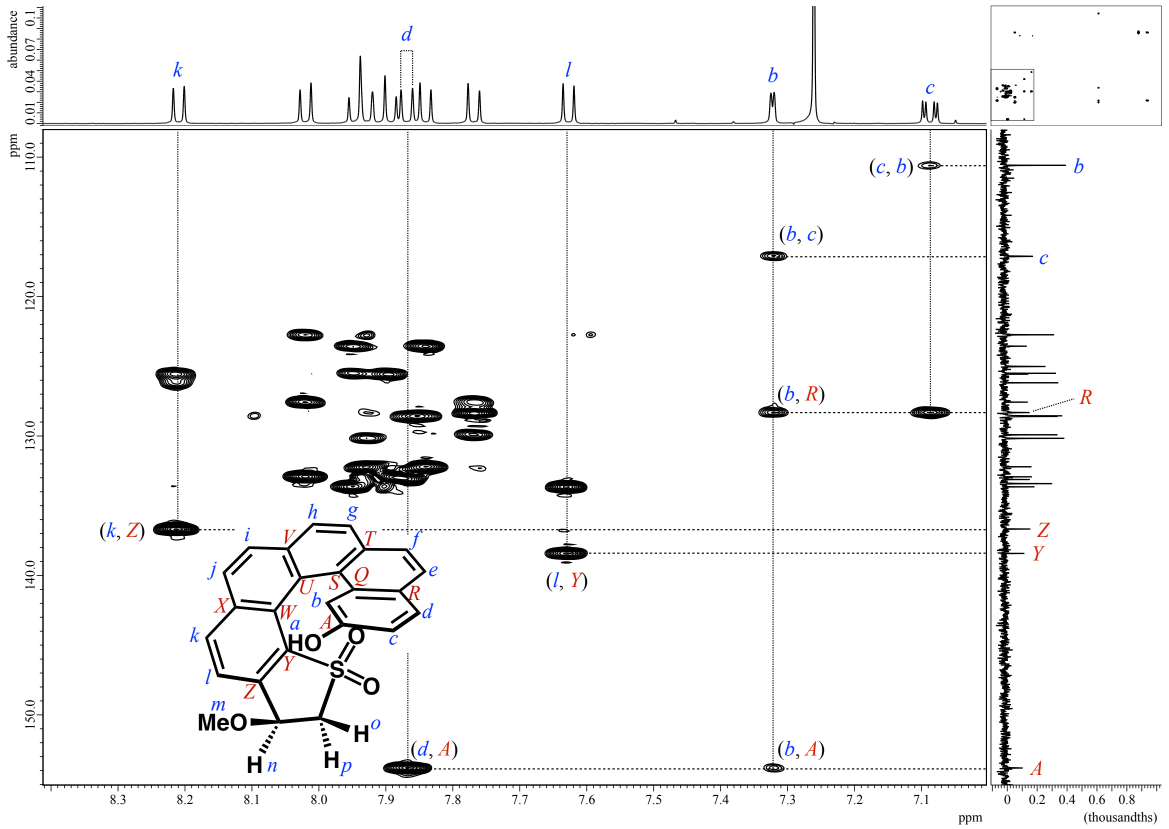

Figure S49. Enlarged ^{1}H–^{13}C HMBC NMR spectrum 1 (500 MHz, $CDCl_3$, 298 K) of **1c'**(α).

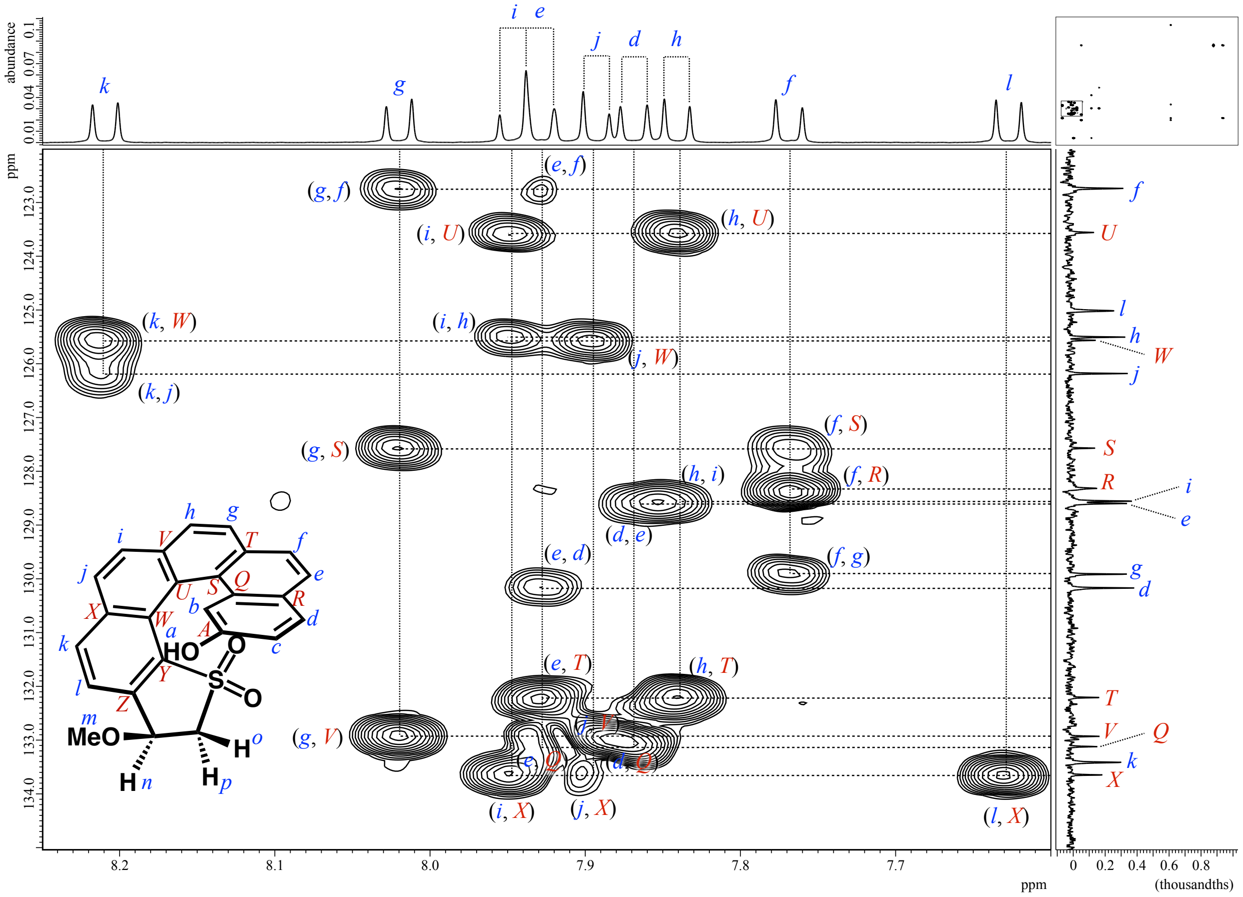

Figure S50. Enlarged ^{1}H–^{13}C HMBC NMR spectrum 2 (500 MHz, $CDCl_3$, 298 K) of **1c'**(α).

Oxa-Michael adduct **1c'**(β)

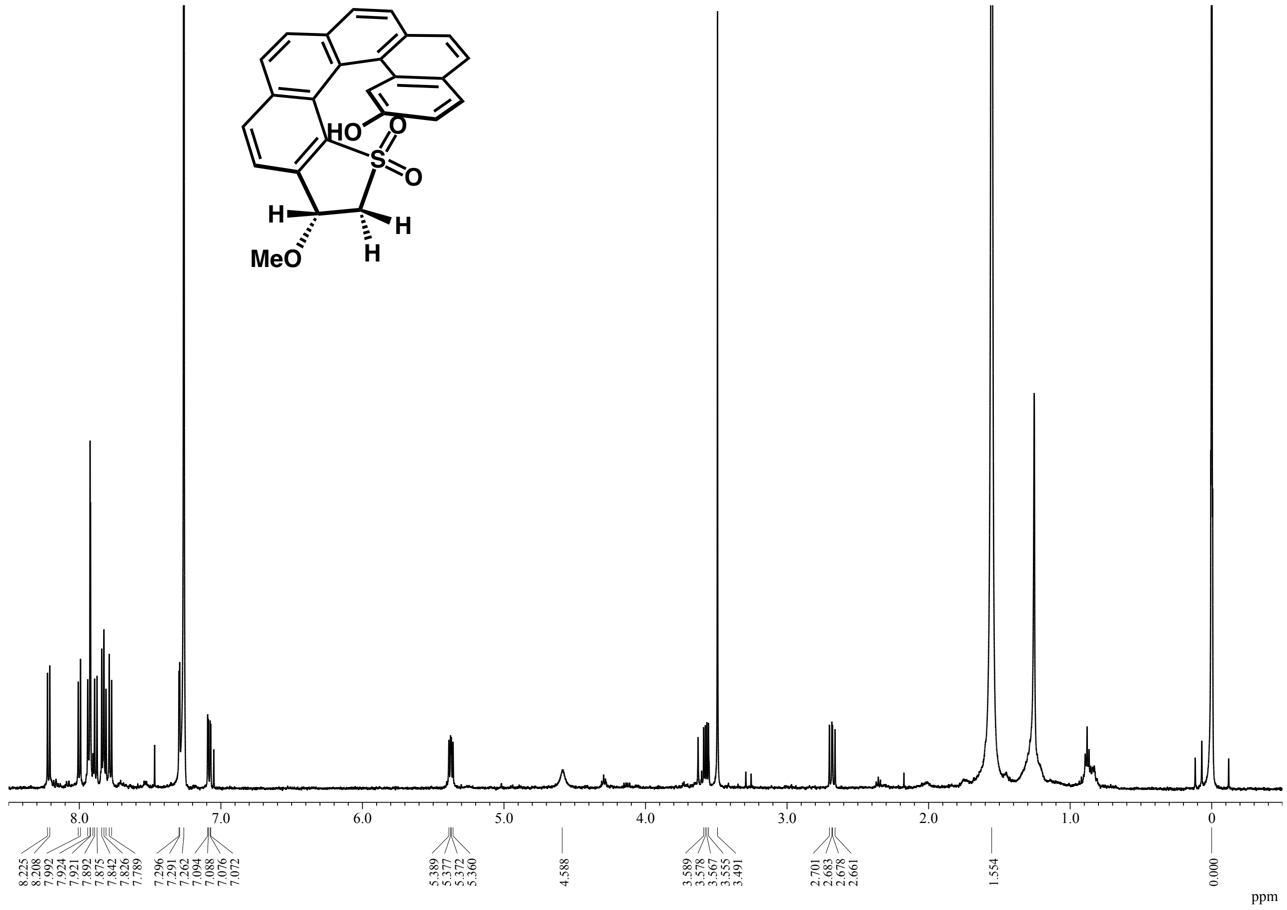

Figure S51. ^{1}H NMR spectrum (500 MHz, $CDCl_3$, 298 K) of **1c'**(β).

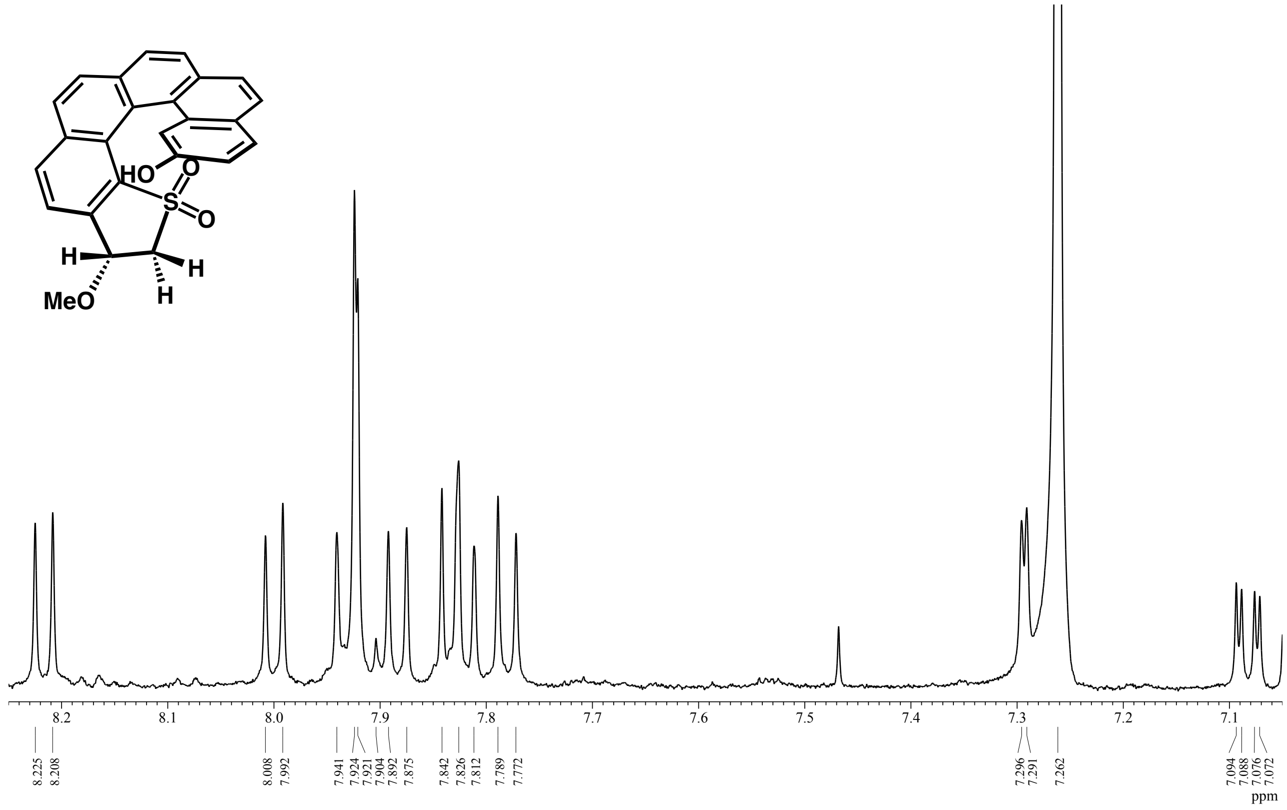

Figure S52. Enlarged ^{1}H NMR spectrum (aromatic region) (500 MHz, $CDCl_3$, 298 K) of **1c'**(β).

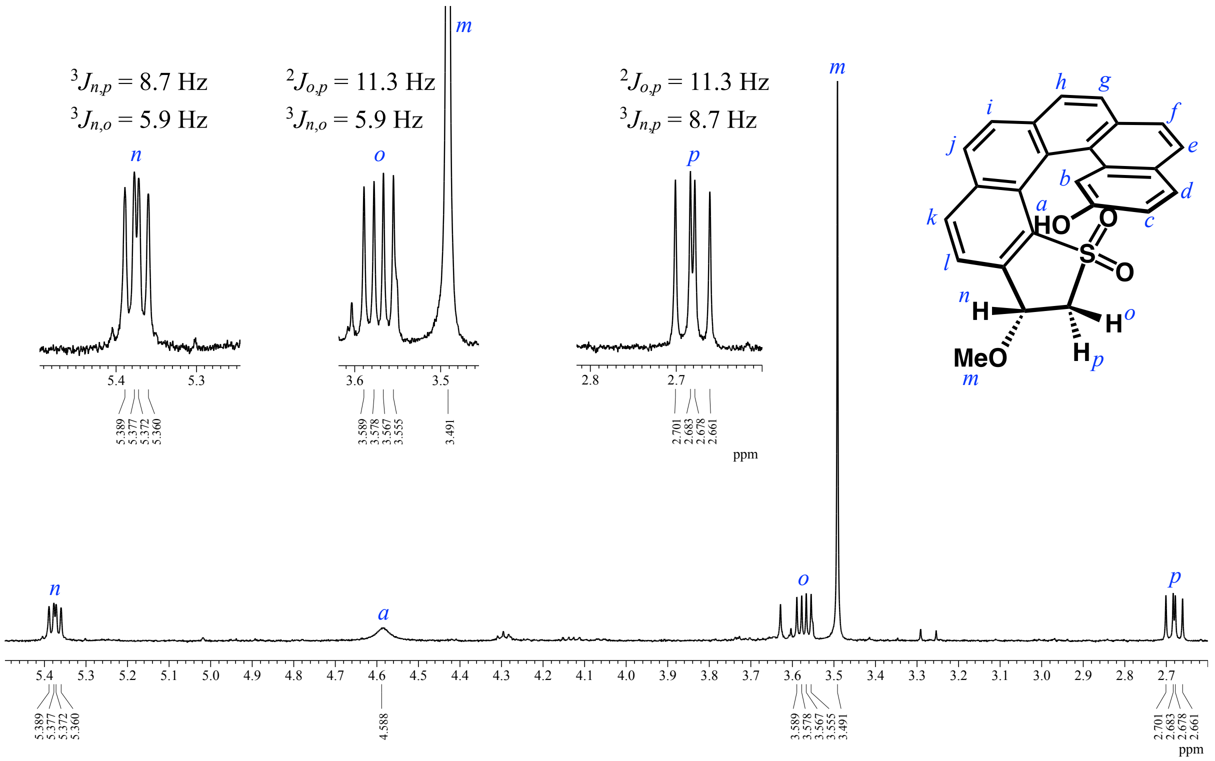

Figure S53. Enlarged ^{1}H NMR spectrum (aliphatic region) (500 MHz, $CDCl_3$, 298 K) of **1c'**(β).

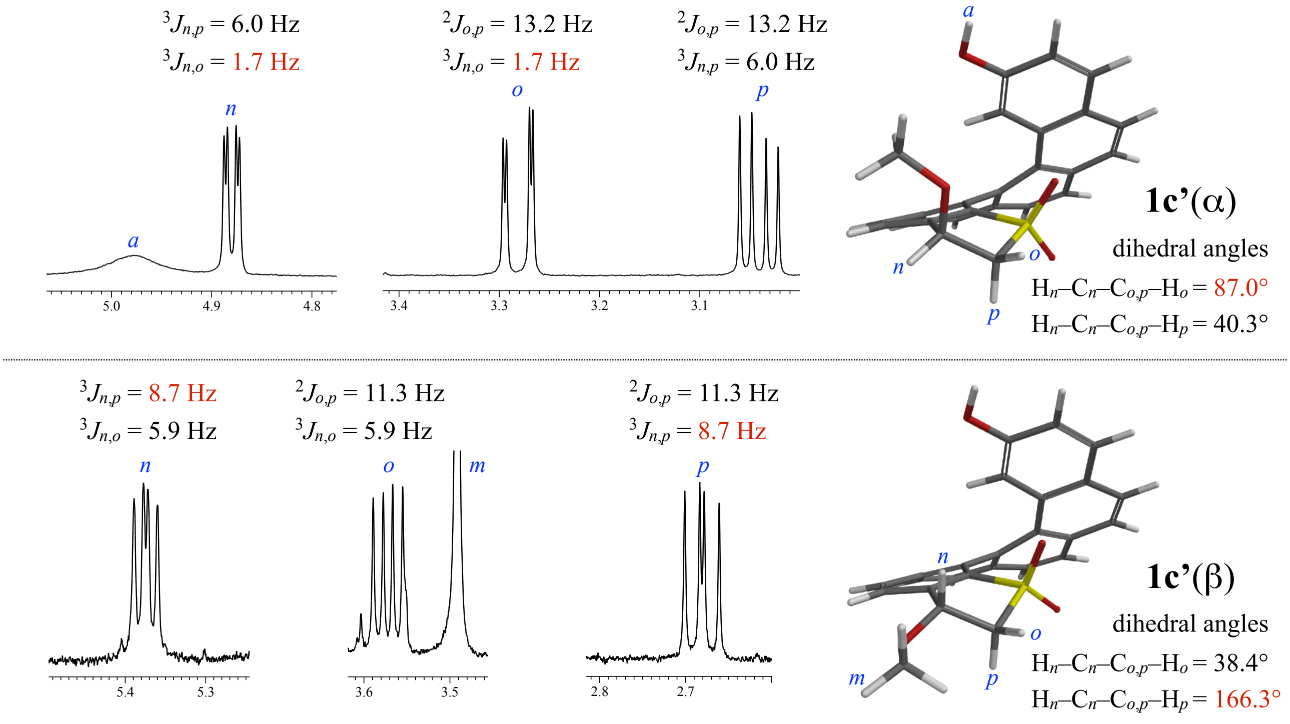

Figure S54. Assignment of the relative stereochemistry of **1c'**(α) and **1c'**(β) by comparison of NMR coupling constants and DFT-derived dihedral angles. Dihedral angles were extracted from DFT-optimized geometries (M06-2X/6-31+G(d,p)). The observed differences in ^{3}J values are consistent with a Karplus-type relationship, where couplings are maximized for near-antiperiplanar arrangements (~180°) and minimized near orthogonal conformations (~90°), enabling stereochemical assignment of **1c'**(α) and **1c'**(β).

5. MS Spectra

Compound **4** TDCMAS ESI-TOF

Analysis Info

		Acquisition Date	2025/09/10 11:15:05
Analysis Name	D:\Data\ofcbunseki\irai\2025\aliance\250911murase\MRS053-000003.d		
Method	esi_posi_low.m	Operator	BDAL@DE
Sample Name	MRS053-	Instrument / Ser#	micrOTOF 213750.10321
Comment			

Acquisition Parameter

Source Type	ESI	Ion Polarity	Positive	Set Nebulizer	0.3 Bar
Focus	Not active			Set Dry Heater	180 ‹C
Scan Begin	50 m/z	Set Capillary	4500 V	Set Dry Gas	4.0 l/min
Scan End	1000 m/z	Set End Plate Offset	-500 V	Set Divert Valve	Waste

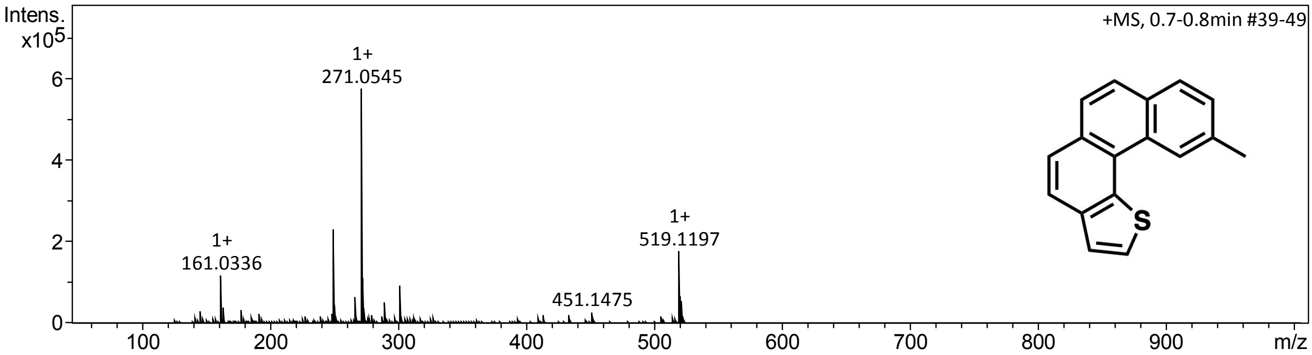

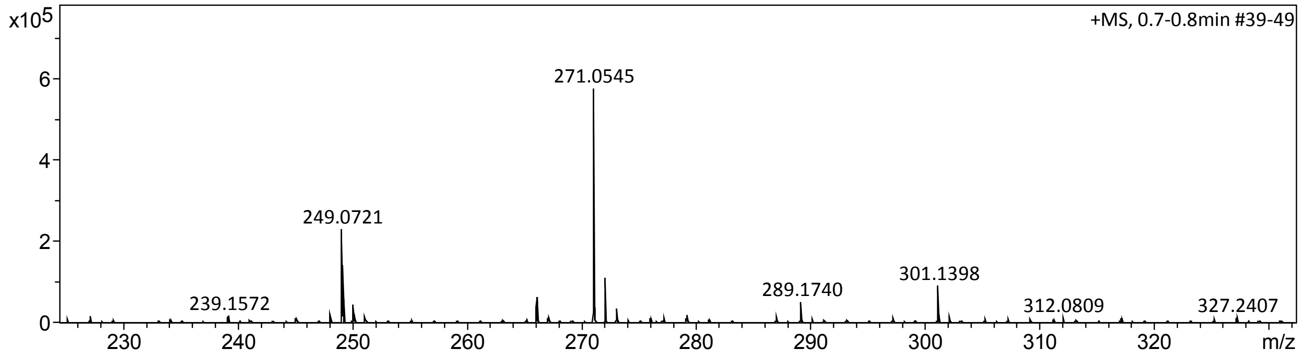

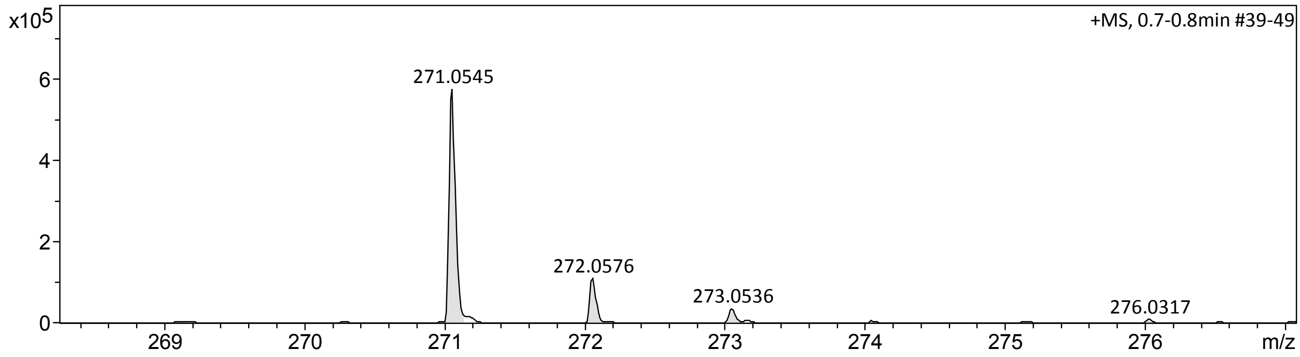

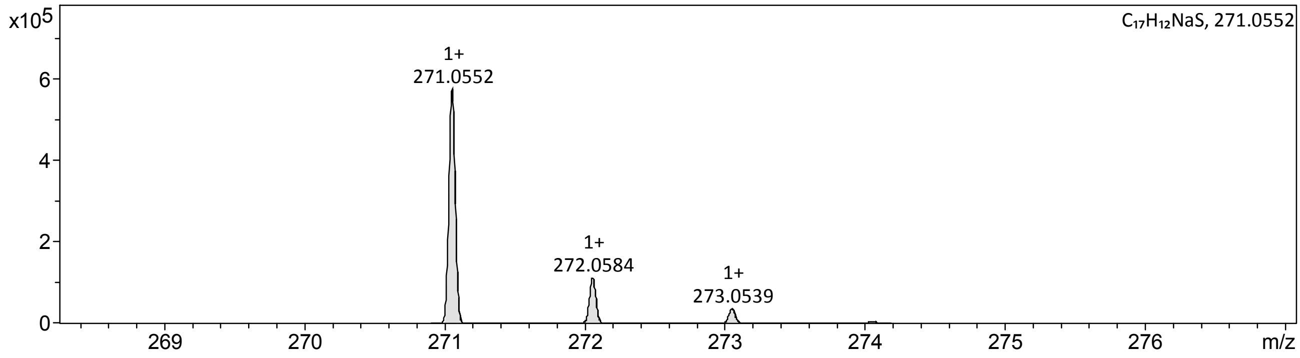

Compound **5**

TDCMAS ESI-TOF

Analysis Info

Analysis Name	D:\Data\ofcbunseki\irai\2025\aliance\250911murase\MRS054-000001.d	Acquisition Date	2025/09/10 11:00:40
Method	esi_posi_low.m	Operator	BDAL@DE
Sample Name	MRS054-	Instrument / Ser#	micrOTOF 213750.10321
Comment			

Acquisition Parameter

Source Type	ESI	Ion Polarity	Positive	Set Nebulizer	0.3 Bar
Focus	Not active			Set Dry Heater	180 ‹C
Scan Begin	50 m/z	Set Capillary	4500 V	Set Dry Gas	4.0 l/min
Scan End	1200 m/z	Set End Plate Offset	-500 V	Set Divert Valve	Waste

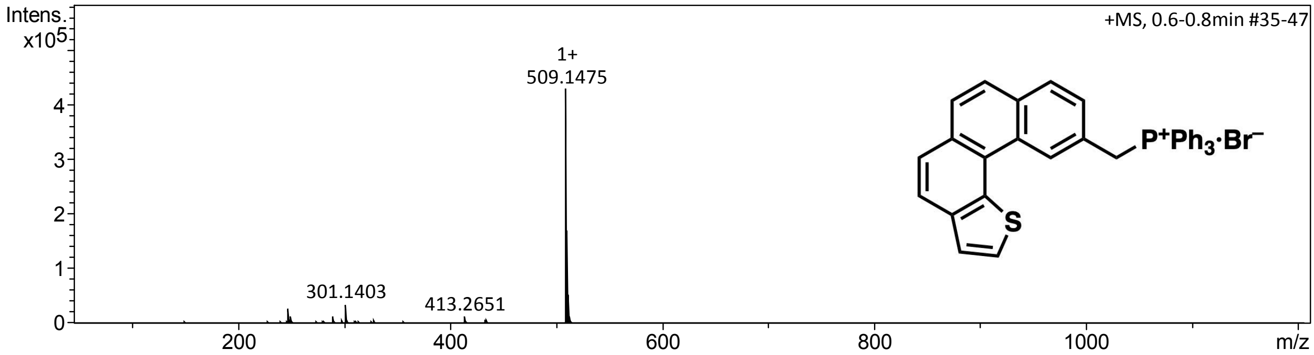

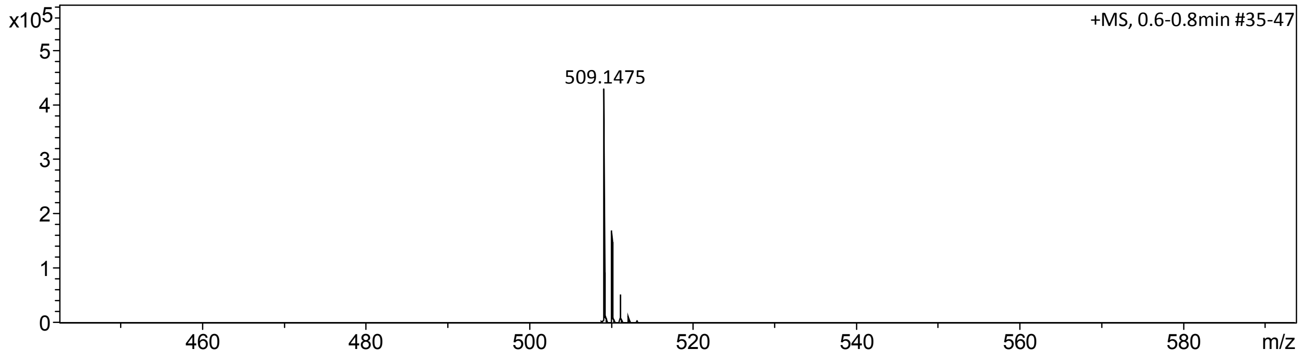

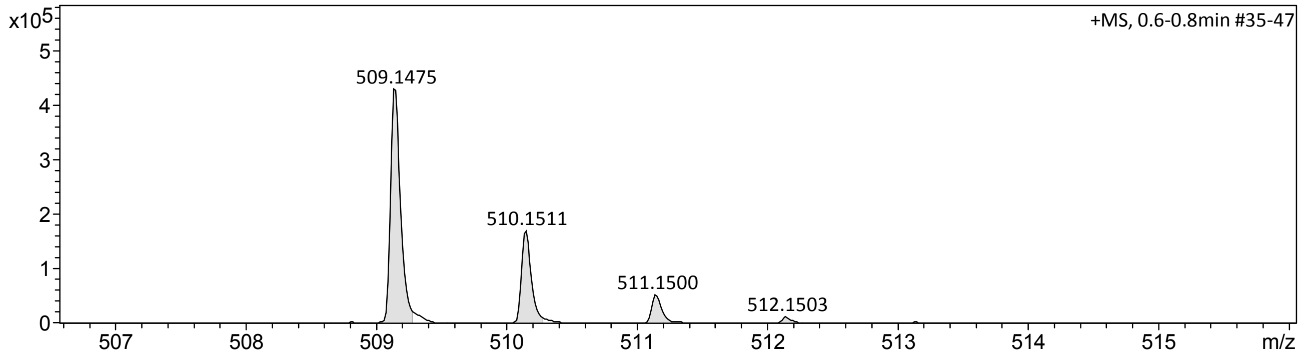

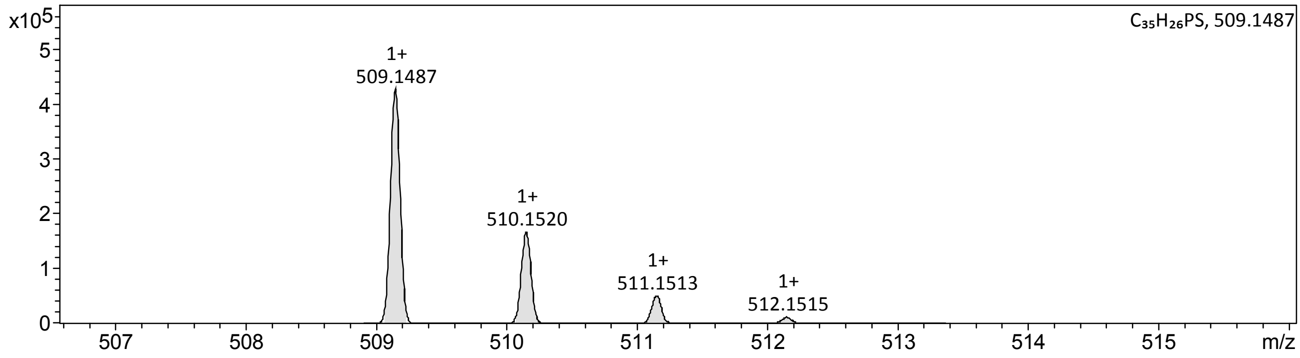

Compound (*Z*)-**6**

TDCMAS ESI-TOF

Analysis Info

Analysis Name	D:\Data\ofcbunseki\irai\2025\aliance\250911murase\MRS055-000001.d	Acquisition Date	2025/09/10 10:22:57
Method	esi_posi_low.m	Operator	BDAL@DE
Sample Name	MRS055-	Instrument / Ser#	micrOTOF 213750.10321
Comment			

Acquisition Parameter

Source Type	ESI	Ion Polarity	Positive	Set Nebulizer	0.3 Bar
Focus	Not active			Set Dry Heater	180 ‹C
Scan Begin	50 m/z	Set Capillary	4500 V	Set Dry Gas	4.0 l/min
Scan End	1000 m/z	Set End Plate Offset	-500 V	Set Divert Valve	Waste

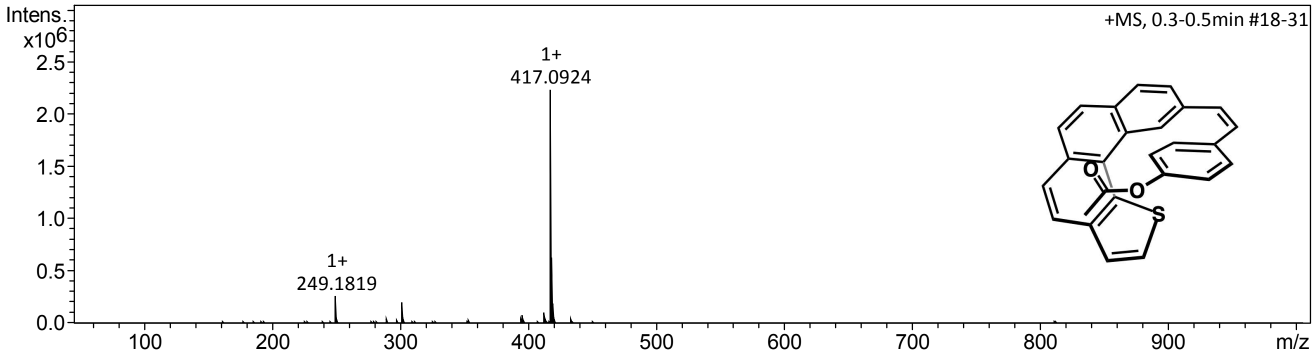

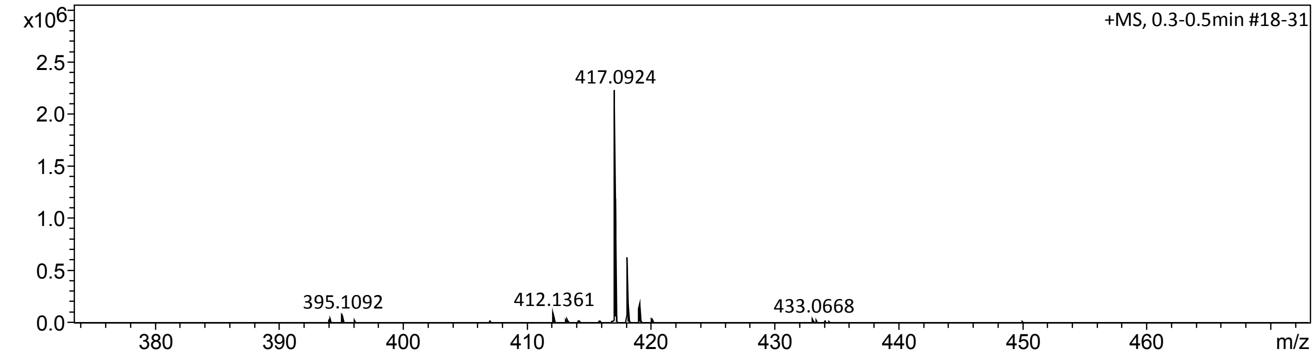

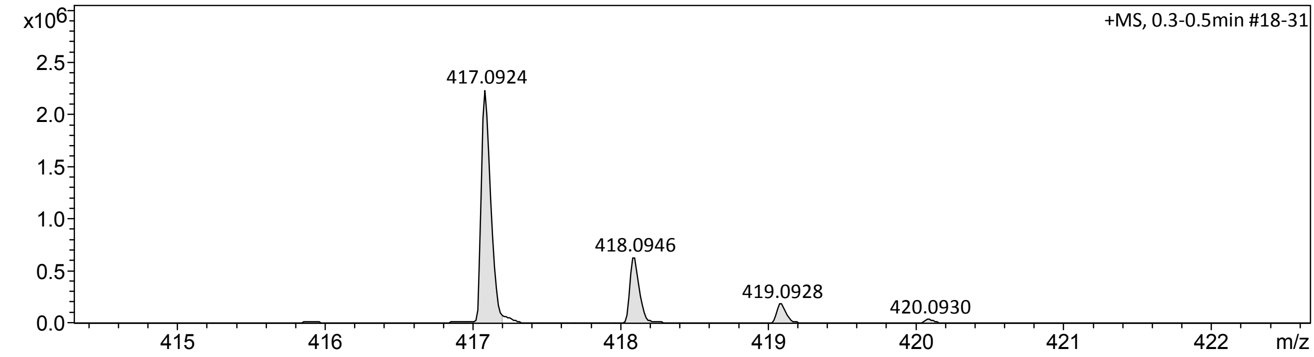

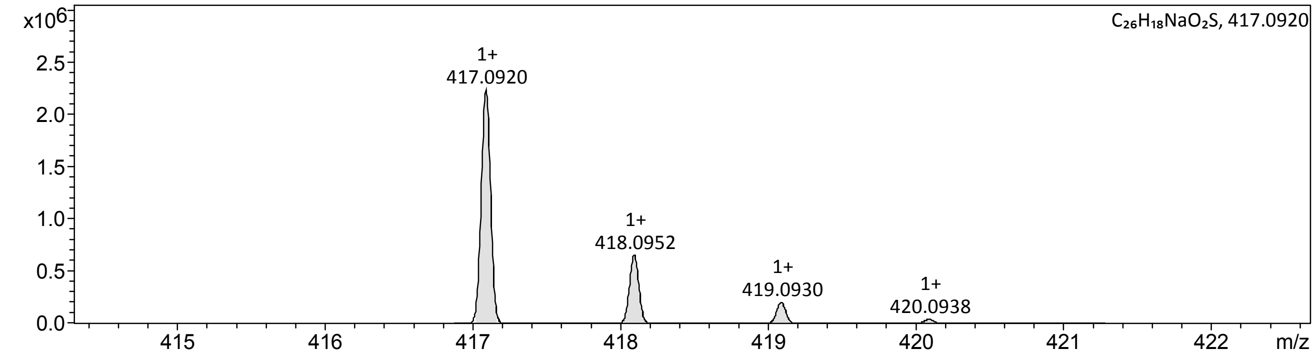

Compound (*E*)-**6**

TDCMAS ESI-TOF

Analysis Info

Analysis Name	D:\Data\ofcbunseki\irai\2025\aliance\250911murase\MRS056-000002.d	Acquisition Date	2025/09/10 10:33:05
Method	esi_posi_low.m	Operator	BDAL@DE
Sample Name	MRS056-	Instrument / Ser#	micrOTOF 213750.10321
Comment			

Acquisition Parameter

Source Type	ESI	Ion Polarity	Positive	Set Nebulizer	0.3 Bar
Focus	Not active			Set Dry Heater	180 ‹C
Scan Begin	50 m/z	Set Capillary	4500 V	Set Dry Gas	4.0 l/min
Scan End	1000 m/z	Set End Plate Offset	-500 V	Set Divert Valve	Waste

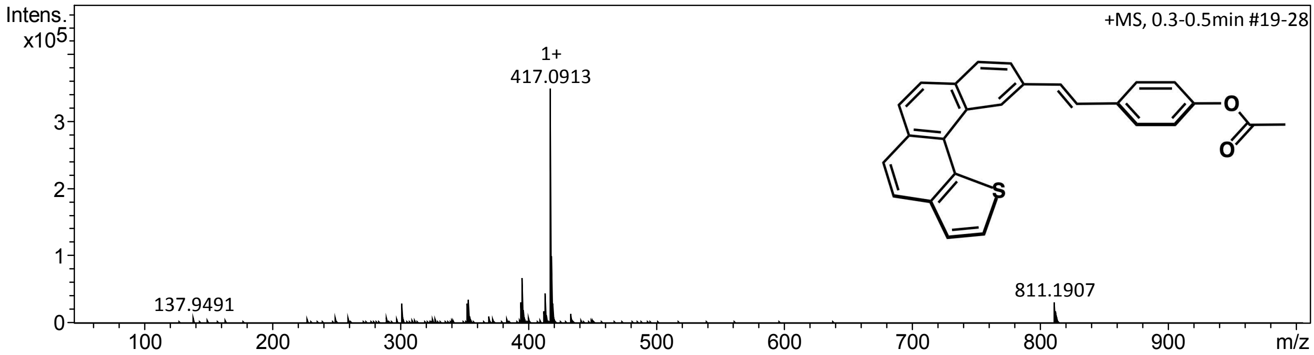

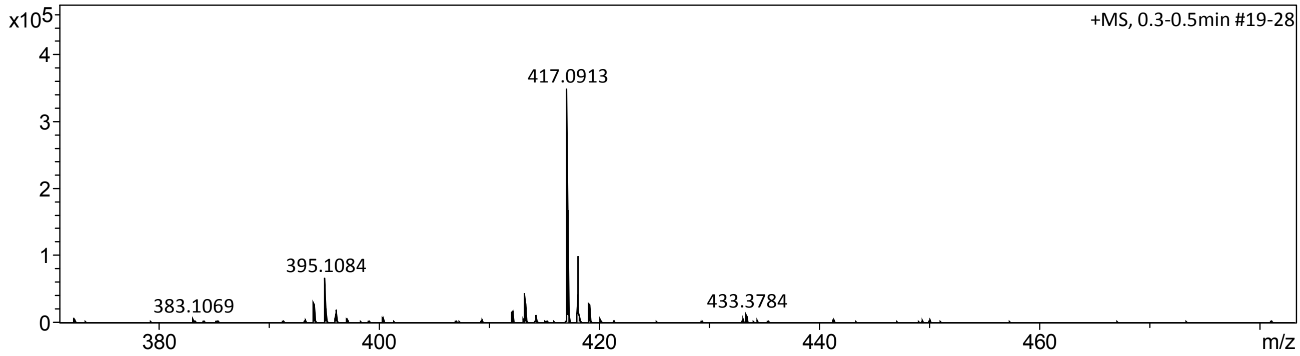

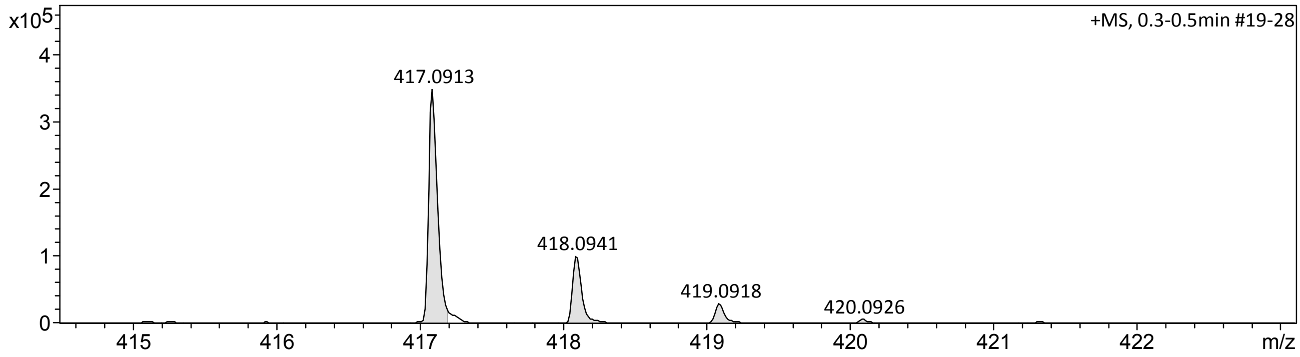

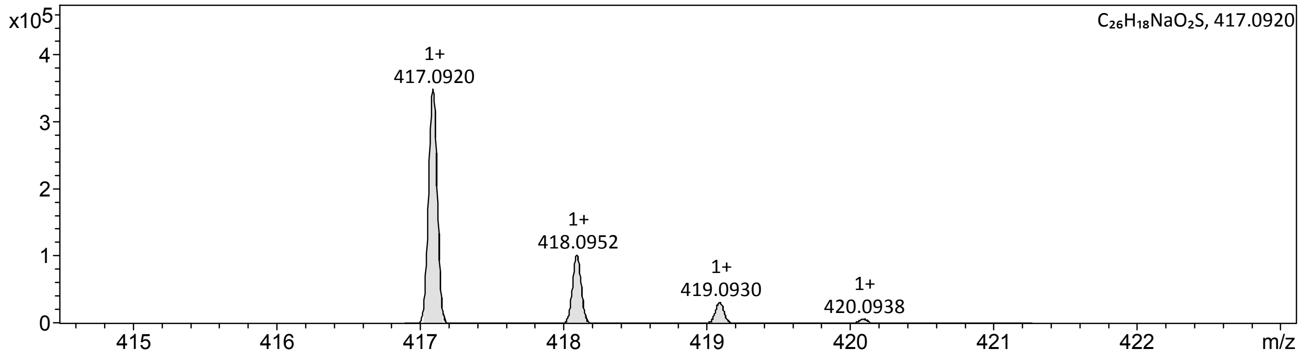

Thia[6]helicene **7**

TDCMAS ESI-TOF

Analysis Info

Analysis Name	D:\Data\ofcbunseki\irai\2025\aliance\250911murase\MRS057-000001.d	Acquisition Date	2025/09/10 10:41:30
Method	esi_posi_low.m	Operator	BDAL@DE
Sample Name	MRS057-	Instrument / Ser#	micrOTOF 213750.10321
Comment			

Acquisition Parameter

Source Type	ESI	Ion Polarity	Positive	Set Nebulizer	0.3 Bar
Focus	Not active			Set Dry Heater	180 ‹C
Scan Begin	50 m/z	Set Capillary	4500 V	Set Dry Gas	4.0 l/min
Scan End	1000 m/z	Set End Plate Offset	-500 V	Set Divert Valve	Waste

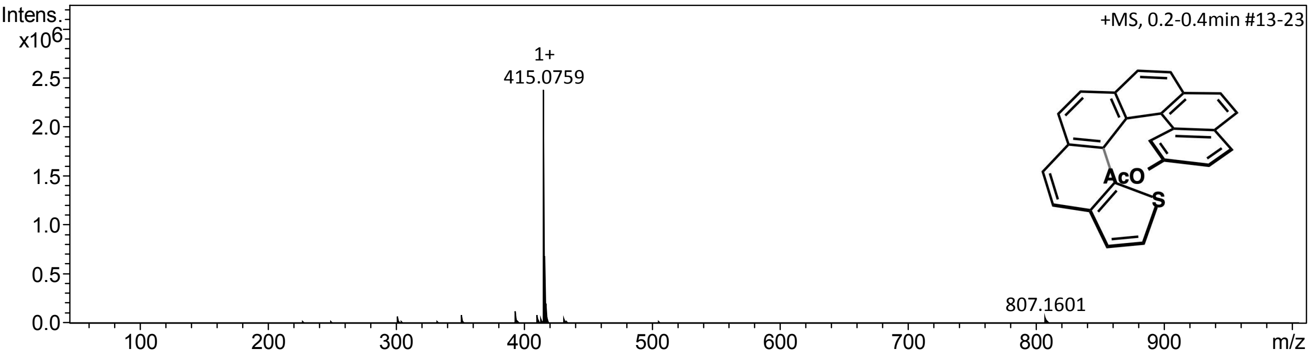

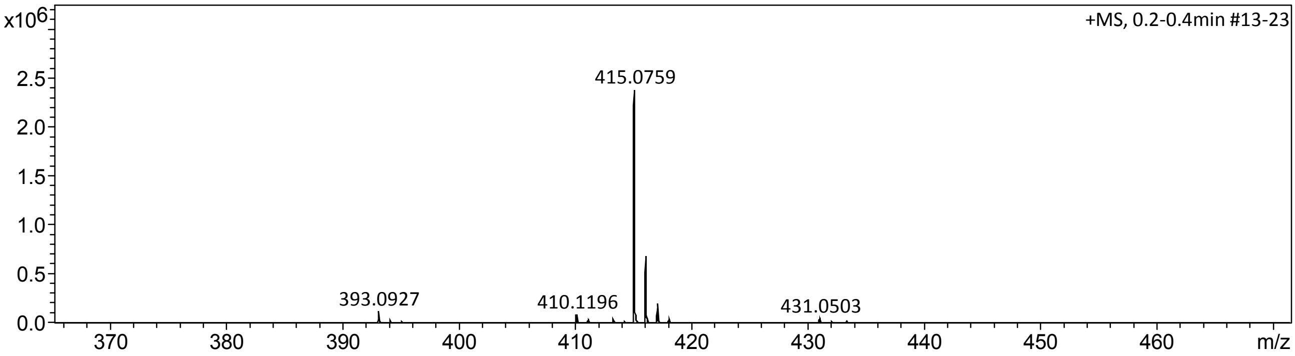

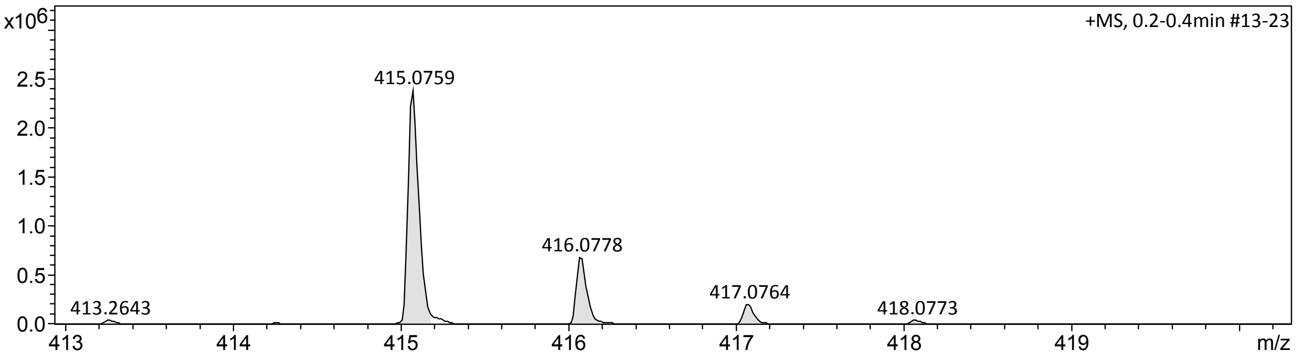

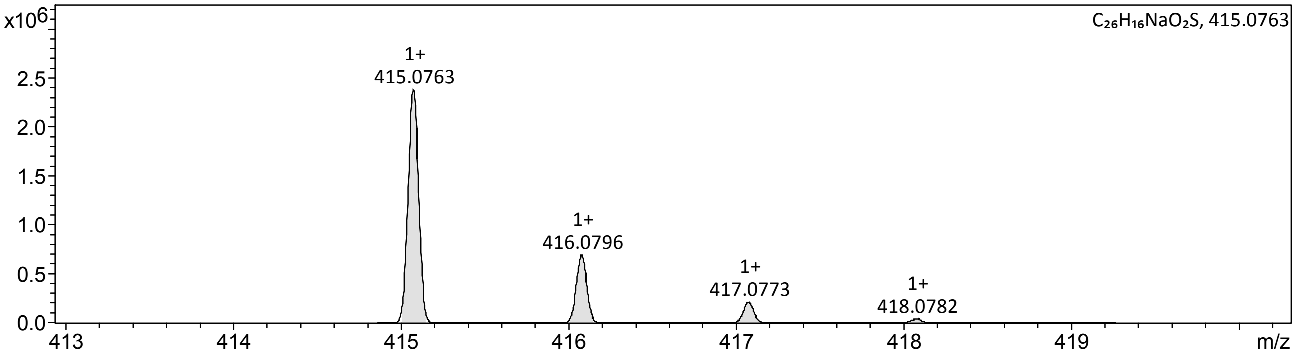

Thia[6]helicene *S*,*S*-dioxide **1d** TDCMAS ESI-TOF

Analysis Info

Analysis Name	D:\Data\ofcbunseki\irai\2025\aliance\250911murase\MRS058-000001.d	Acquisition Date	2025/09/10 10:51:24
Method	esi_posi_low.m	Operator	BDAL@DE
Sample Name	MRS058-	Instrument / Ser#	micrOTOF 213750.10321
Comment			

Acquisition Parameter

Source Type	ESI	Ion Polarity	Positive	Set Nebulizer	0.3 Bar
Focus	Not active			Set Dry Heater	180 ‹C
Scan Begin	50 m/z	Set Capillary	4500 V	Set Dry Gas	4.0 l/min
Scan End	1000 m/z	Set End Plate Offset	-500 V	Set Divert Valve	Waste

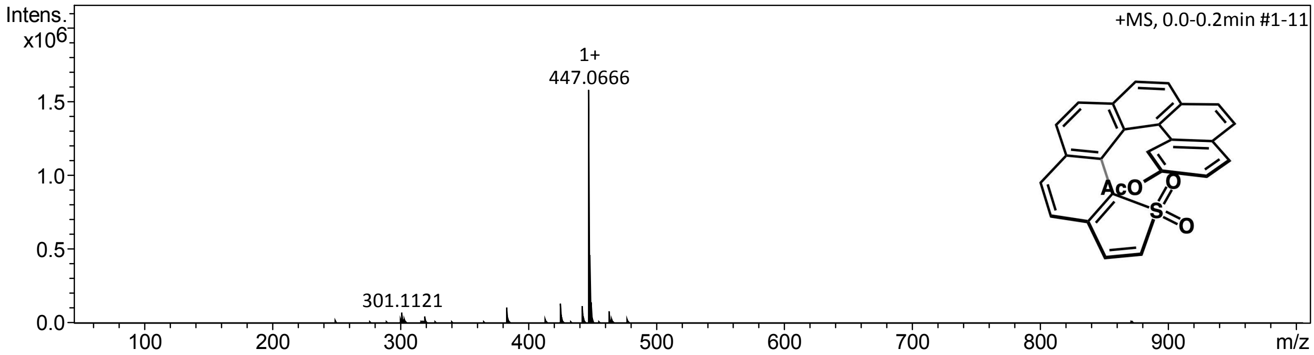

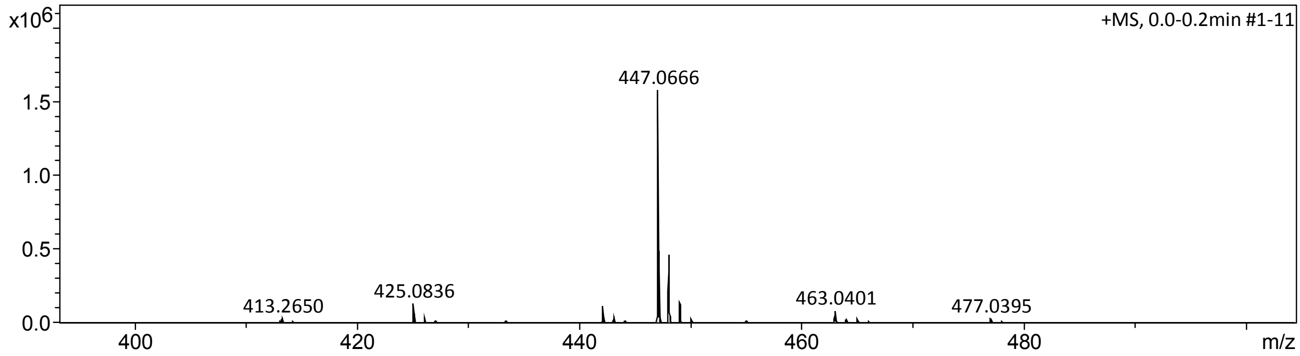

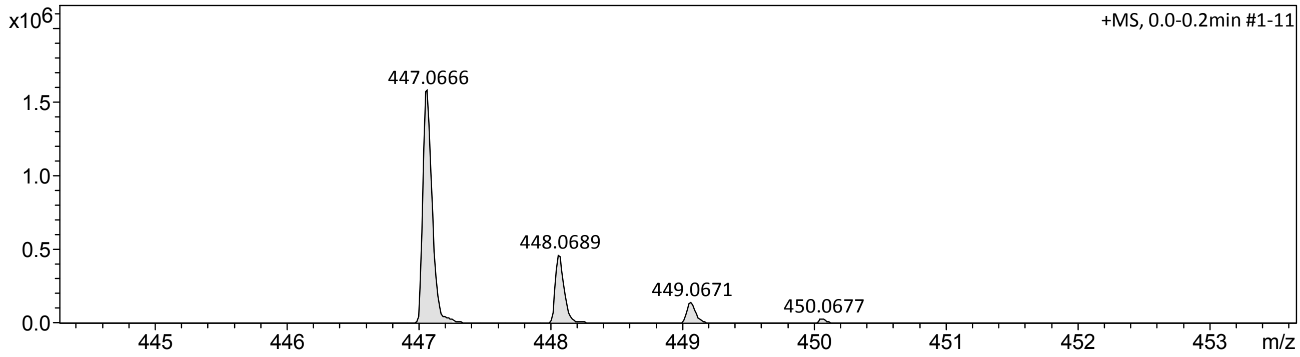

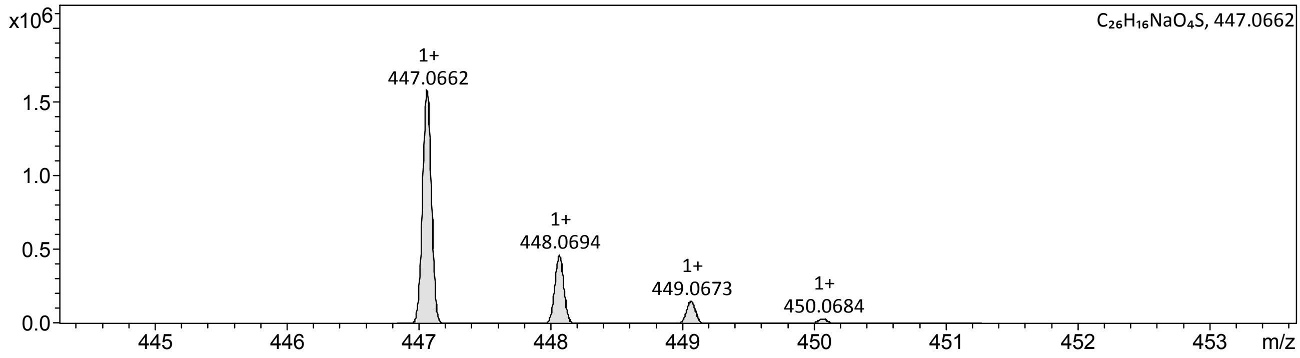

Thia[6]helicene *S*,*S*-dioxide **1c** TDCMAS ESI-TOF

Analysis Info

Analysis Name	D:\Data\ofcbunseki\irai\2025\aliance\251127murase\MRS-066-000001.d	Acquisition Date	2025/11/26 9:56:49
Method	esi_posi_low.m	Operator	BDAL@DE
Sample Name	MRS-066-	Instrument / Ser#	micrOTOF 213750.10321
Comment			

Acquisition Parameter

Source Type	ESI	Ion Polarity	Positive	Set Nebulizer	0.3 Bar
Focus	Not active			Set Dry Heater	180 ‹C
Scan Begin	50 m/z	Set Capillary	4500 V	Set Dry Gas	4.0 l/min
Scan End	1000 m/z	Set End Plate Offset	-500 V	Set Divert Valve	Waste

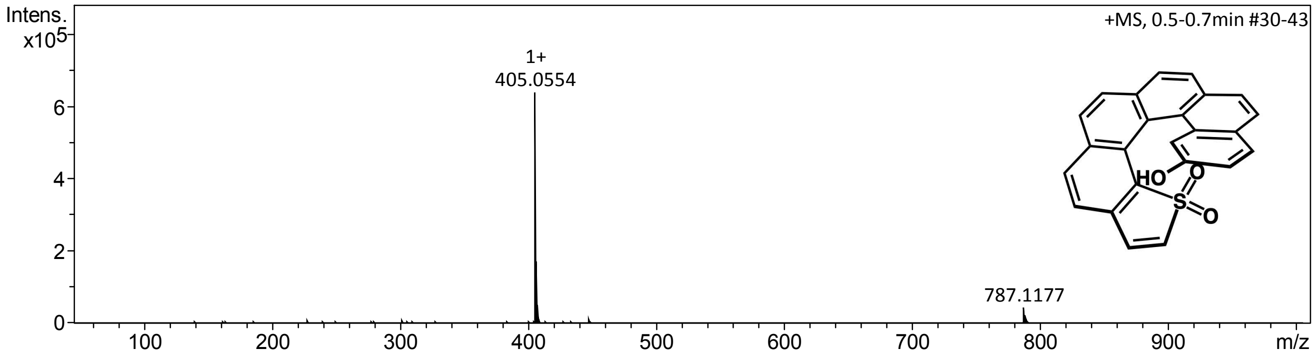

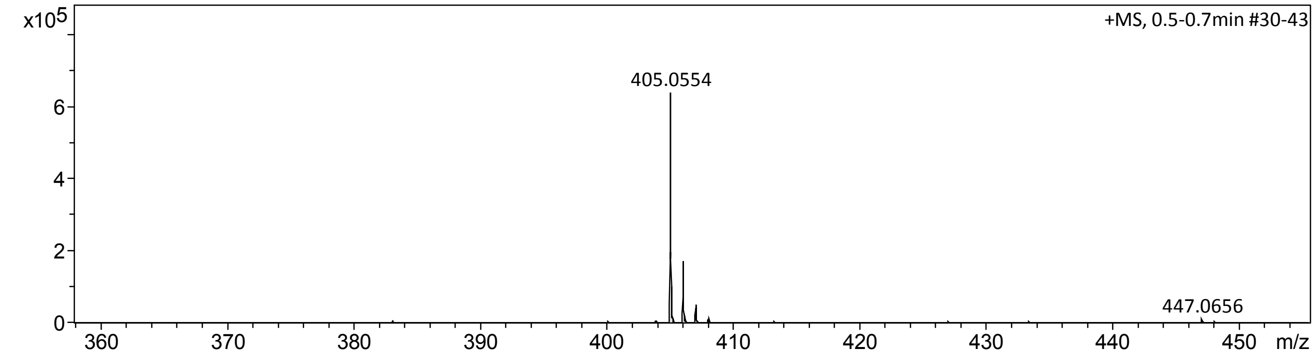

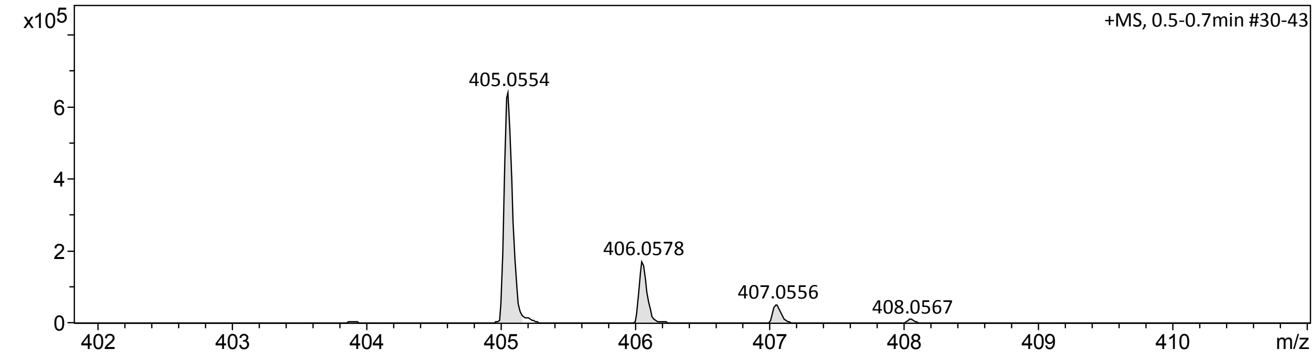

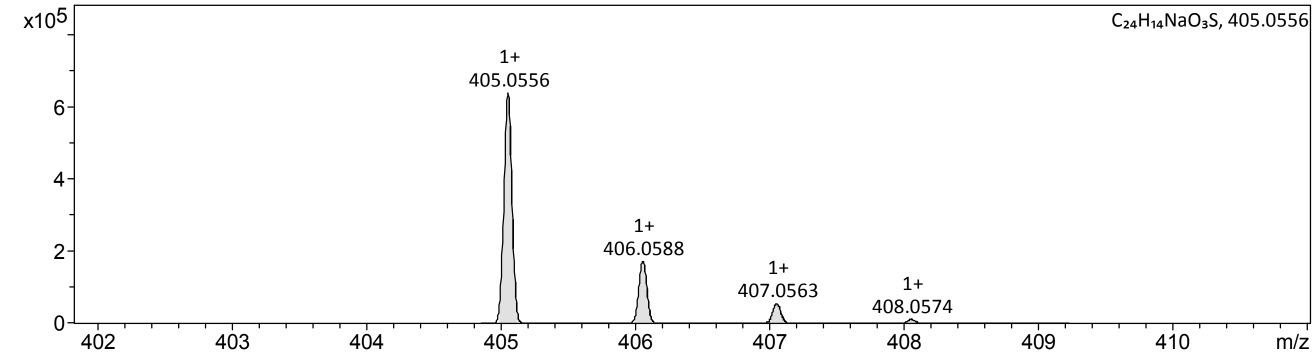

Oxa-Michael adduct **1c'** (α) TDCMAS ESI-TOF

Analysis Info

Analysis Name	D:\Data\ofcbunseki\irai\2025\aliance\260121murase\MRS071-000001.d	Acquisition Date	2026/01/20 9:08:28
Method	esi_posi_low.m	Operator	BDAL@DE
Sample Name	MRS071-	Instrument / Ser#	micrOTOF 213750.10321
Comment			

Acquisition Parameter

Source Type	ESI	Ion Polarity	Positive	Set Nebulizer	0.3 Bar
Focus	Not active			Set Dry Heater	180 ‹C
Scan Begin	50 m/z	Set Capillary	4500 V	Set Dry Gas	4.0 l/min
Scan End	1000 m/z	Set End Plate Offset	-500 V	Set Divert Valve	Waste

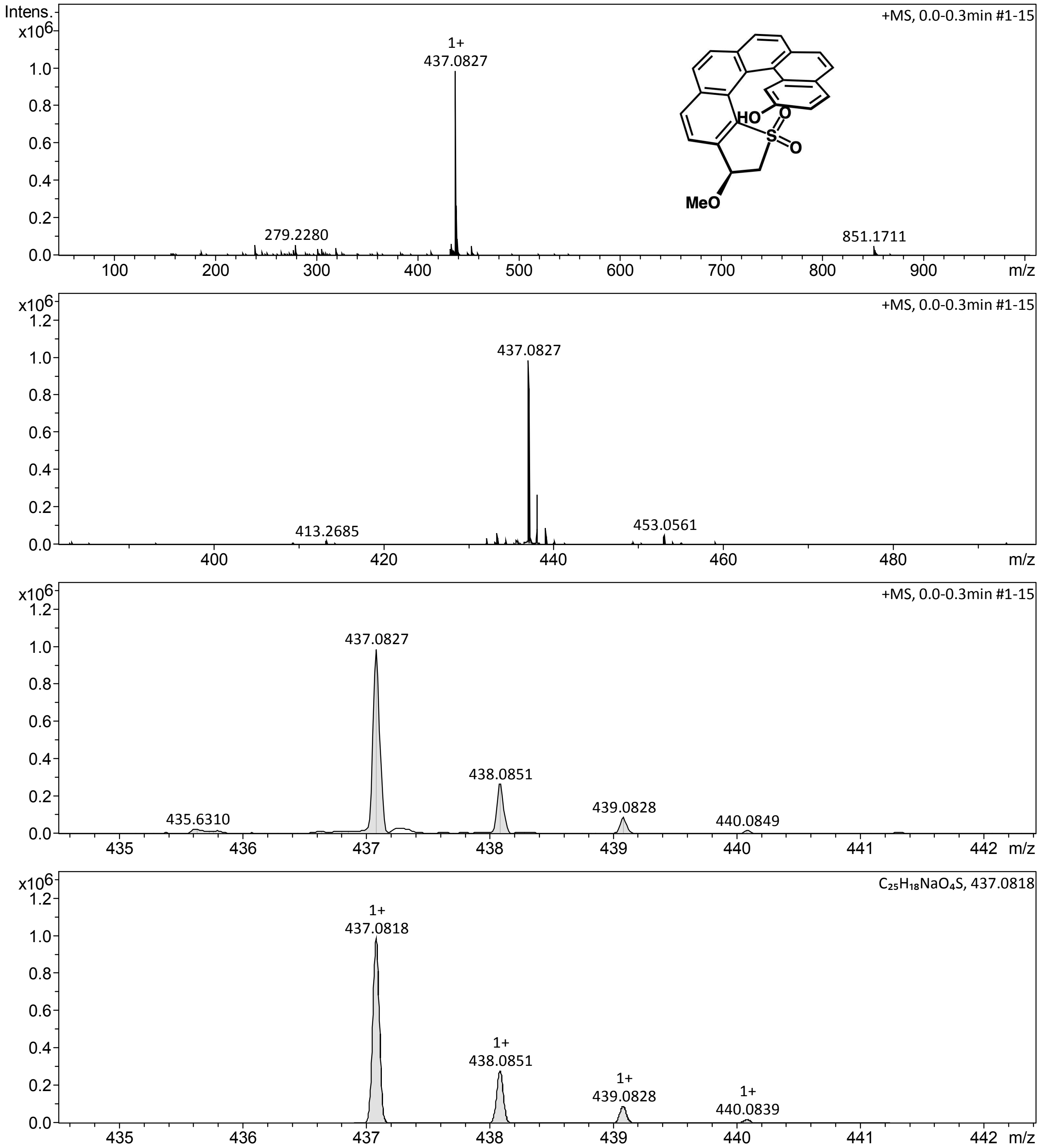

Oxa-Michael adduct **1c'** (β) TDCMAS ESI-TOF

Analysis Info

Analysis Name	D:\Data\ofcbunseki\irai\2025\aliance\260121murase\MRS072-000003.d	Acquisition Date	2026/01/20 9:24:43
Method	esi_posi_low.m	Operator	BDAL@DE
Sample Name	MRS072-	Instrument / Ser#	micrOTOF 213750.10321
Comment			

Acquisition Parameter

Source Type	ESI	Ion Polarity	Positive	Set Nebulizer	0.3 Bar
Focus	Not active			Set Dry Heater	180 ‹C
Scan Begin	50 m/z	Set Capillary	4500 V	Set Dry Gas	4.0 l/min
Scan End	1000 m/z	Set End Plate Offset	-500 V	Set Divert Valve	Waste

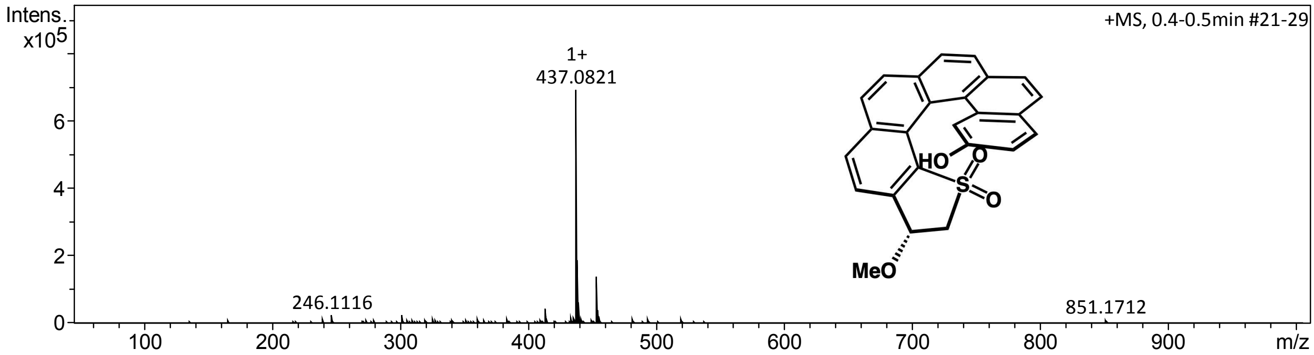

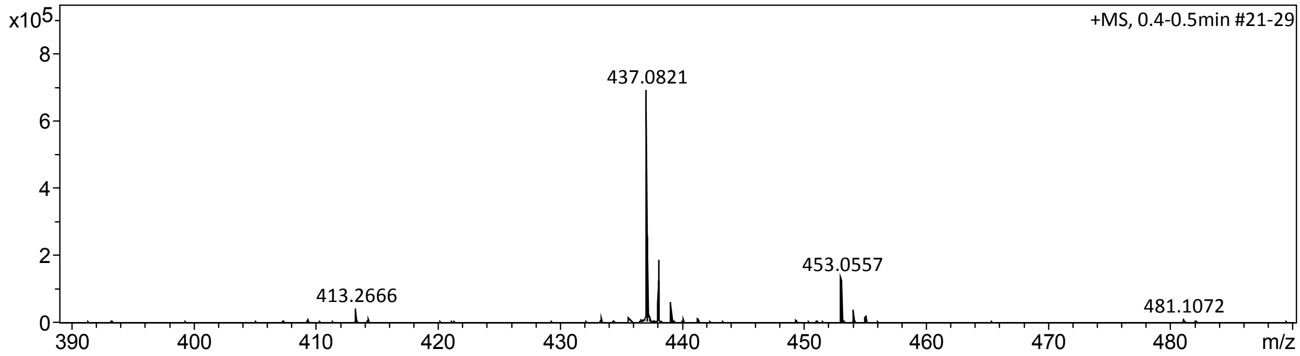

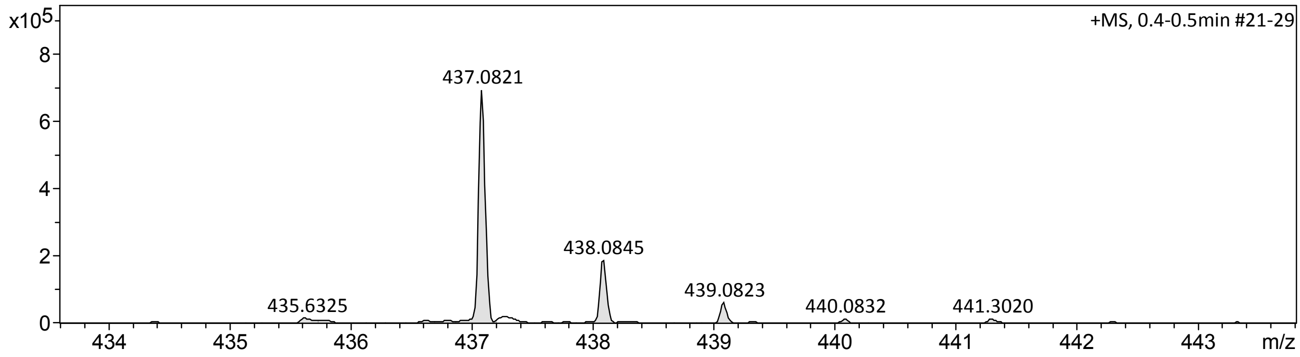

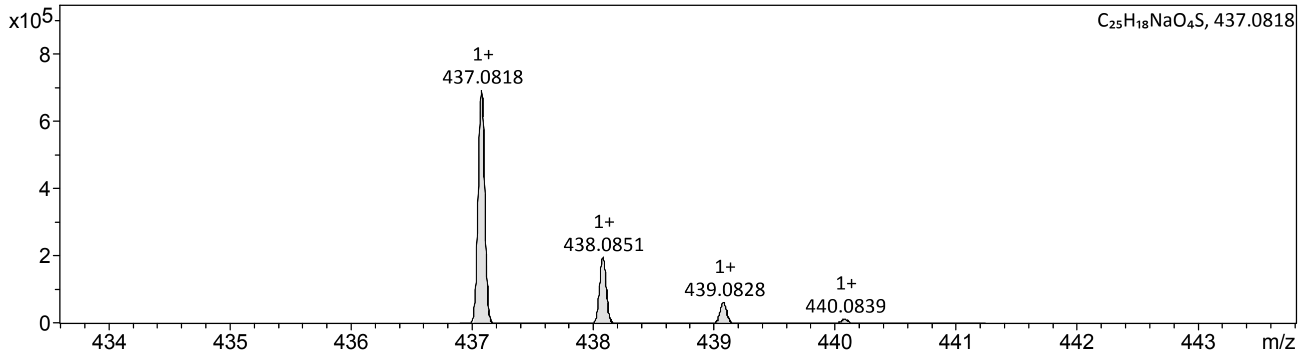

6. References

[S1] S. Wang, N. Lokesh, J. Hioe, R. M. Gschwind, B. König, *Chem. Sci.* **2019**, *10*, 4580–4587.

[S2] Y. Zhao, D. G. Truhlar, *Theor. Chem. Acc.* **2008**, *120*, 215–241.

[S3] R. Sure, S. Grimme, *J. Chem. Theory Comput.* **2015**, *11*, 3785–3801.
